# Supplementary figures and images for: FMRP deficiency leads to multifactorial dysregulation of splicing and mislocalization of MBNL1 to the cytoplasm
Source: PLoS Biol. 2023 Dec 4;21(12):e3002417. doi: 10.1371/journal.pbio.3002417 (PMC10721184; doi:10.1371/journal.pbio.3002417)

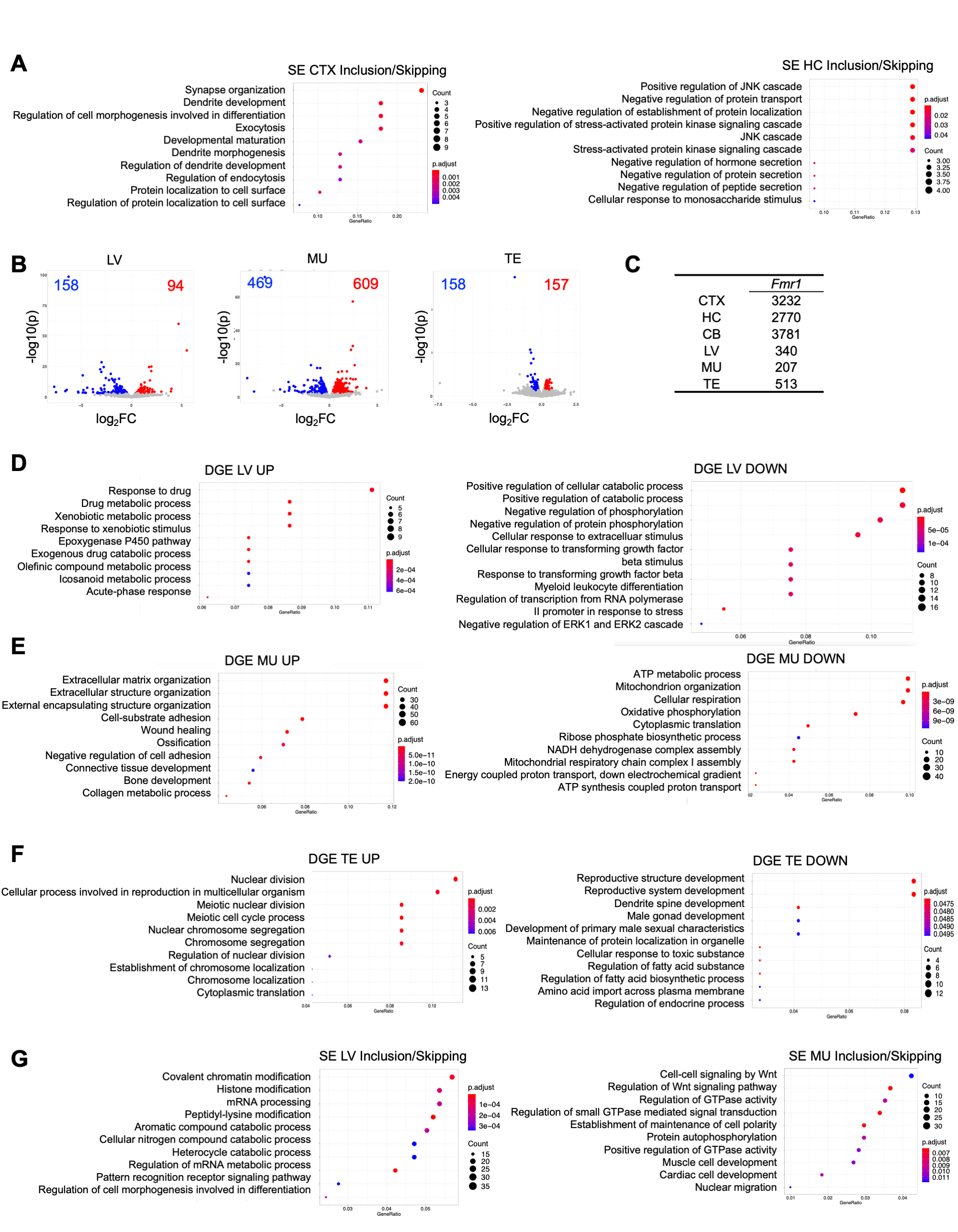

Supplement: S1 Fig — (A) GO terms for skipped/included exons in Fmr1 KO CTX and HC. (B) Volcano plots of RNAs up- or down-regulated (log2FC > 0.2 or < −0.2, FDR<0.05, n = 3) in Fmr1 KO liver (LV), muscle (MU), and testis (TE). (C) Fmr1 RNA levels (TPM) in brain regions and peripheral tissues. (D–F) GO terms of RNAs up- or down-regulated in Fmr1 KO LV, MU, and TE. (G) GO terms for skipped/included exons in Fmr1 KO LV and MU. (TIF) [file pbio.3002417.s001.tif]

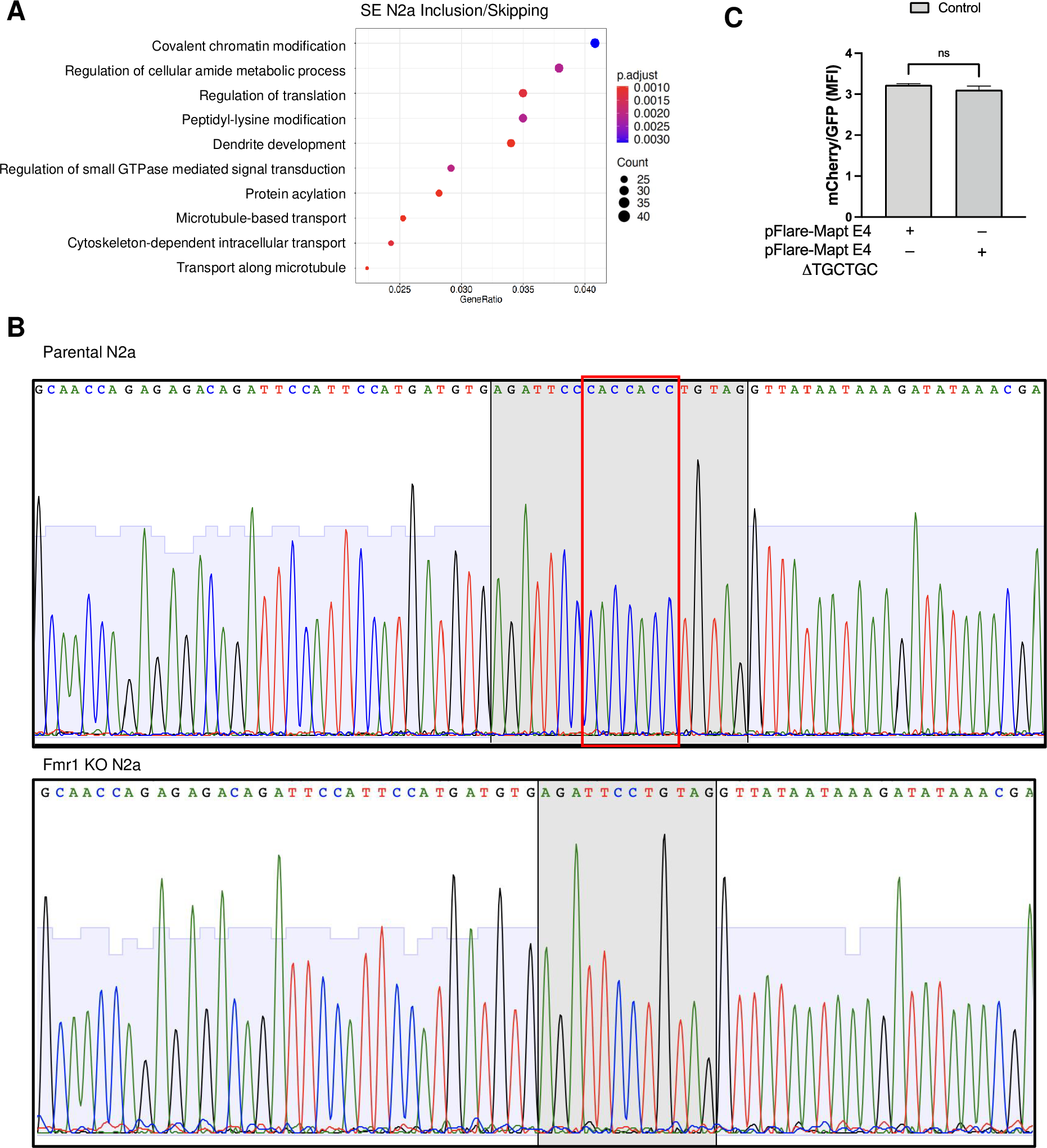

Supplement: S2 Fig — (A) GO terms for RNAs that display exon skipping in Fmr1-depleted N2A cells. (B) DNA sequence analysis of parental N2A cells (top) and Fmr1-depleted cells (bottom). The shaded portion corresponds the amino acid sequences shown in Fig 2. The red box indicates the nucleotides depleted by CRISPR/Cas9 editing. Following editing, the TAG at the right of the shaded box becomes a premature stop codon, leading to nonsense mediated mRNA decay and loss of FMRP expression. (C) Mean fluorescence intensity (MFI) of mCherry/GFP evaluated by flow cytometry in control cells transfected with a splicing reporter with an MBNL1-binding site deletion (UGCUGC). The underlying data can be found in S3 Data. (TIF) [file pbio.3002417.s002.tif]

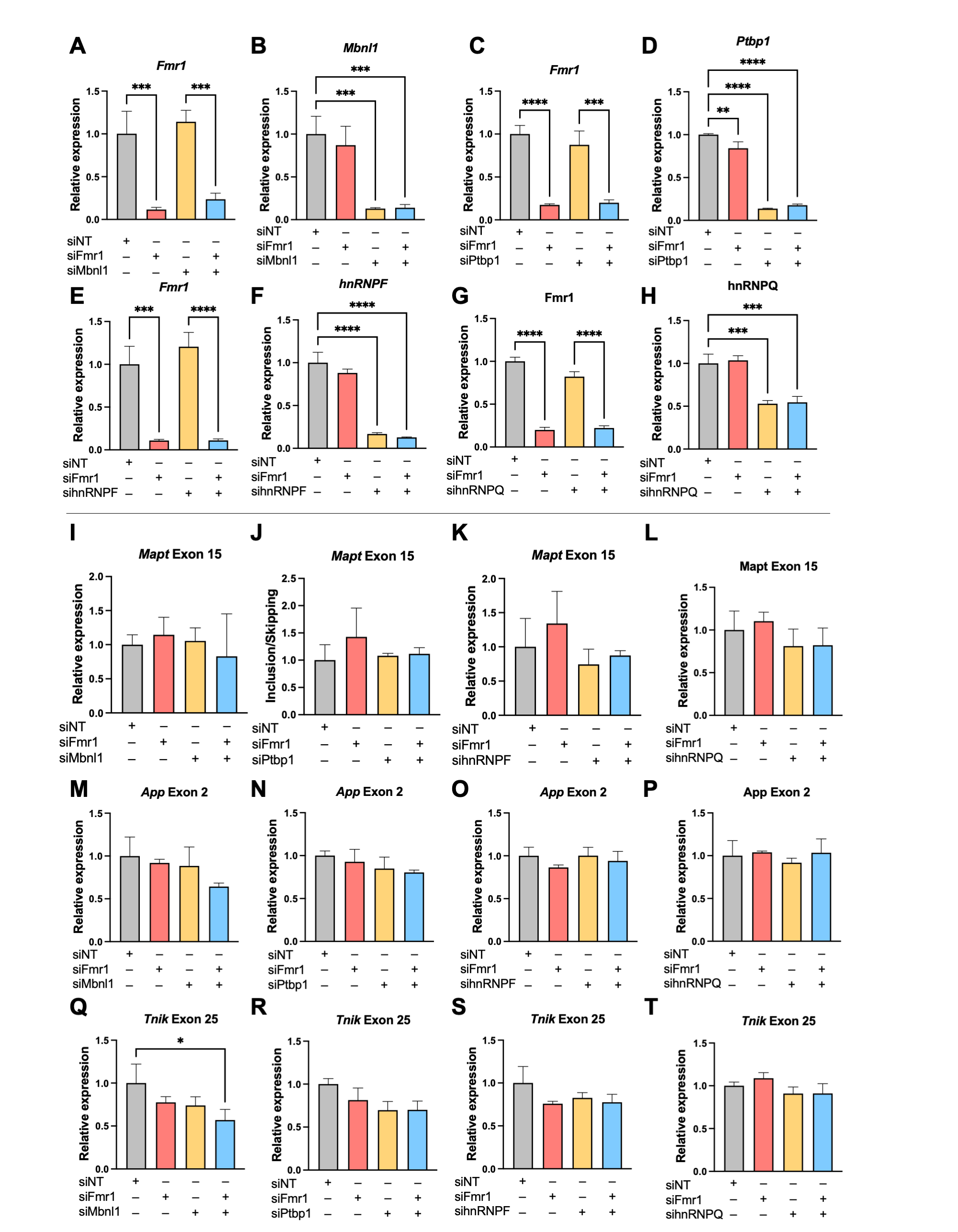

Supplement: S3 Fig — (A–H) Efficacy of Fmr1 and splicing factor depletion by siRNAs. **p < 0.01; ***p < 0.001; ****p < 0.0001 (n = 3). (I–T) Determination of skipping of constitutive exons Mapt exon 15, App exon 2, and Tnik exon 25 following Fmr1 and splicing factor depletion. *p < 0.05 (n = 3). The underlying data can be found in S3 Data. (TIF) [file pbio.3002417.s003.tif]

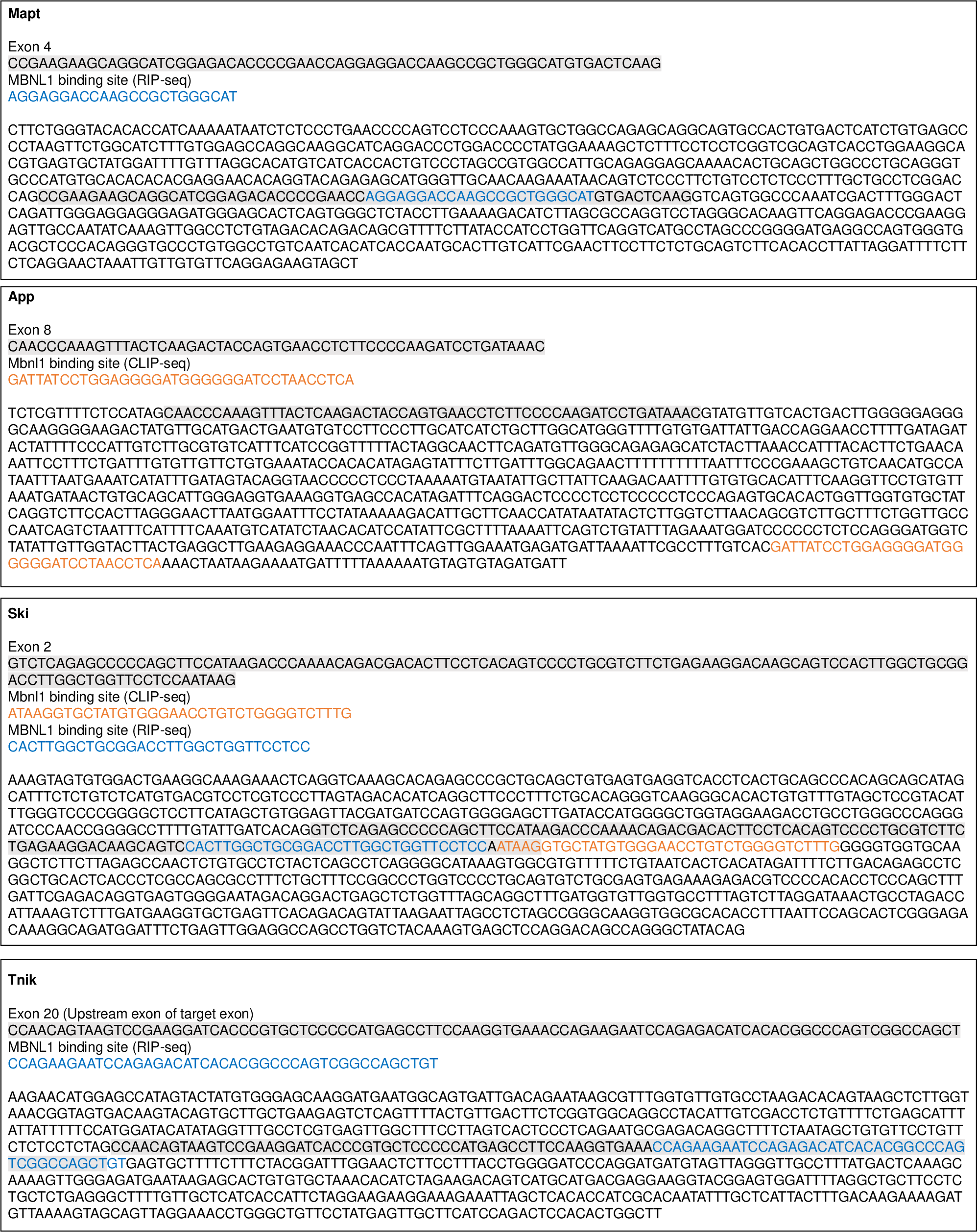

Supplement: S4 Fig — Analysis of MBNL1-binding sites was conducted using data from MBNL1 RIP-seq, with binding sites highlighted in blue, and CLIP-seq, with binding sites indicated in orange [28,29]. These binding sites were analyzed in exons located near Fmr1-regulated splicing events that display alterations which are shaded in gray. (TIF) [file pbio.3002417.s004.tif]

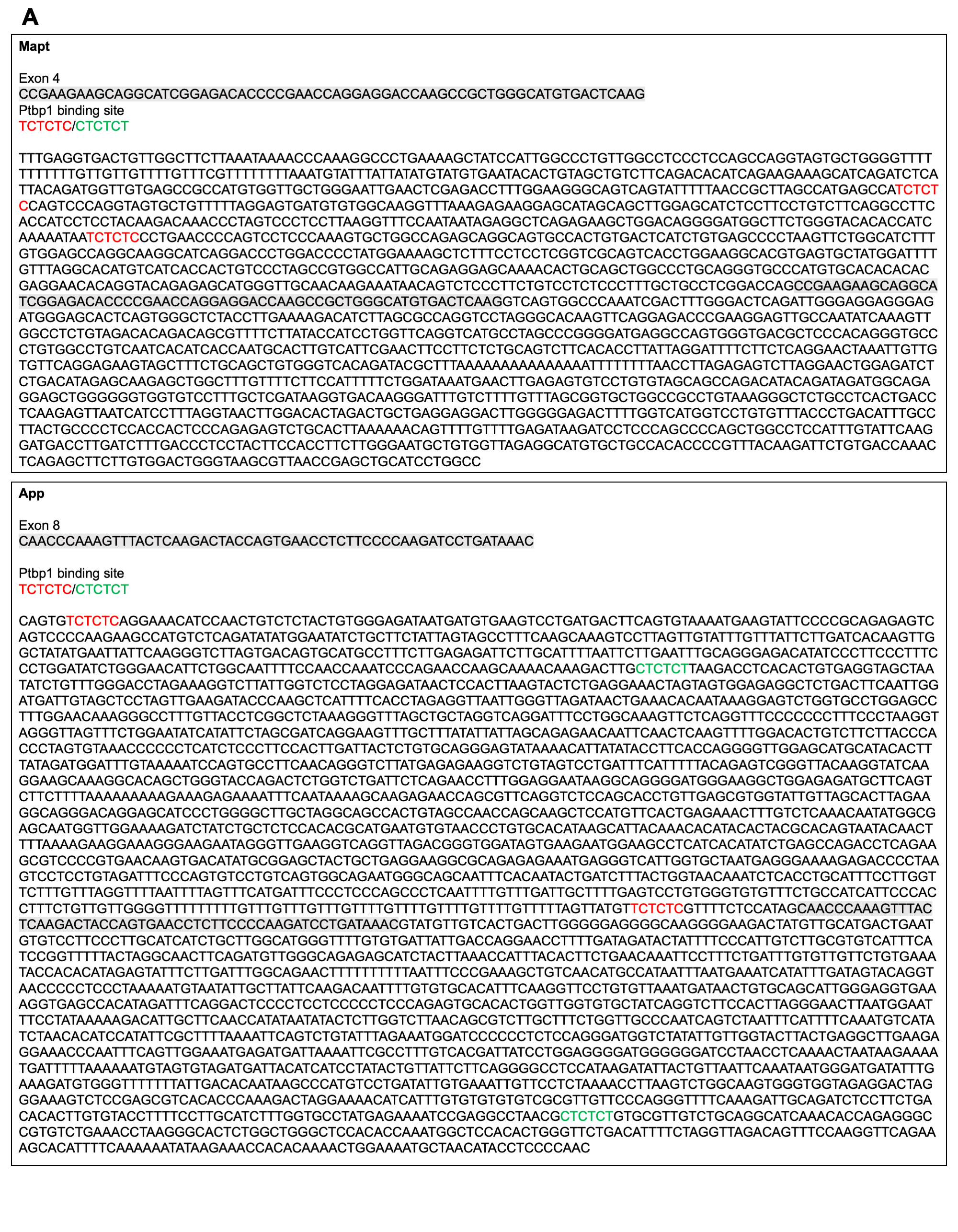

Supplement: S5 Fig — Analysis of PTBP1-binding sites using 6-mer binding motifs TCTCTC and CTCTCT. The binding sites are highlighted in red and green, respectively. These binding sites were analyzed in exons located near Fmr1-regulated splicing events that display alterations which are shaded in gray. (TIFF) [file pbio.3002417.s005.tiff]

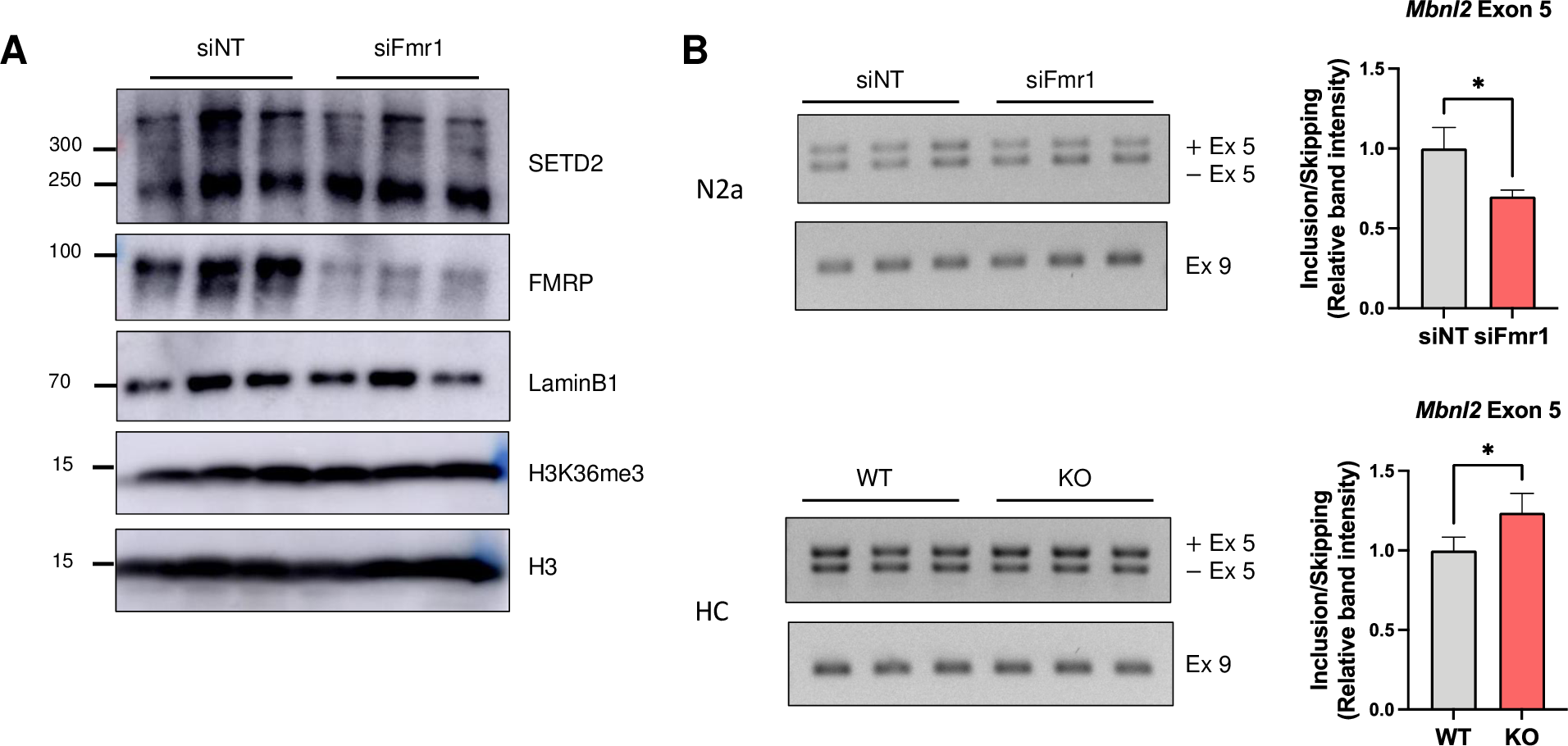

Supplement: S6 Fig — (A) Western blot for SETD2, H3K36me3, H3, and FMRP in nuclear extracts of control and Fmr1-deficient N2A cells. LaminB1 is used as a loading control. (B) RT-PCR for Mbnl2 exon 5 splicing in control and Fmr1-deficient N2A cells and WT and Fmr1 KO HC. Band intensity of exon 5 is quantified and mean ± SD is shown (Student’s t test, *p < 0.05). The constitutive exon 9 was amplified to compare total Mbnl2 RNA expression. The underlying data can be found in S3 Data. (TIF) [file pbio.3002417.s006.tif]

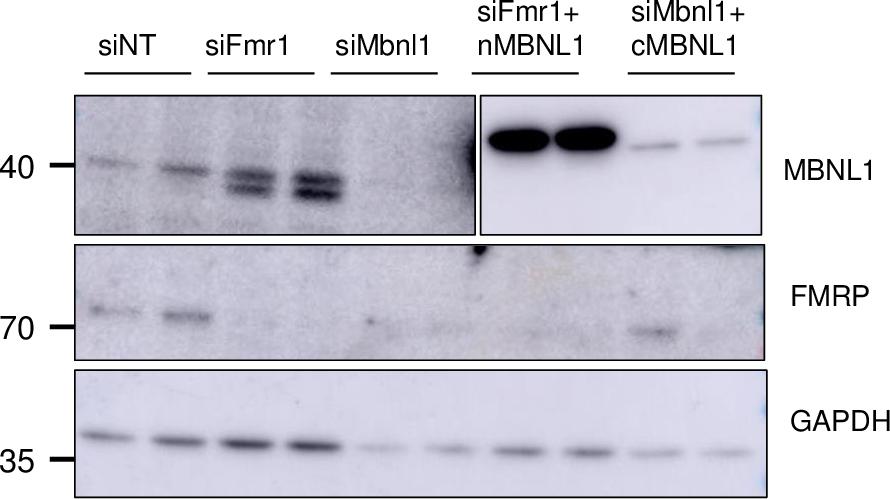

Supplement: S7 Fig — Western blot for FMRP and MBNL1. GAPDH is used as a loading control. (TIF) [file pbio.3002417.s007.tif]

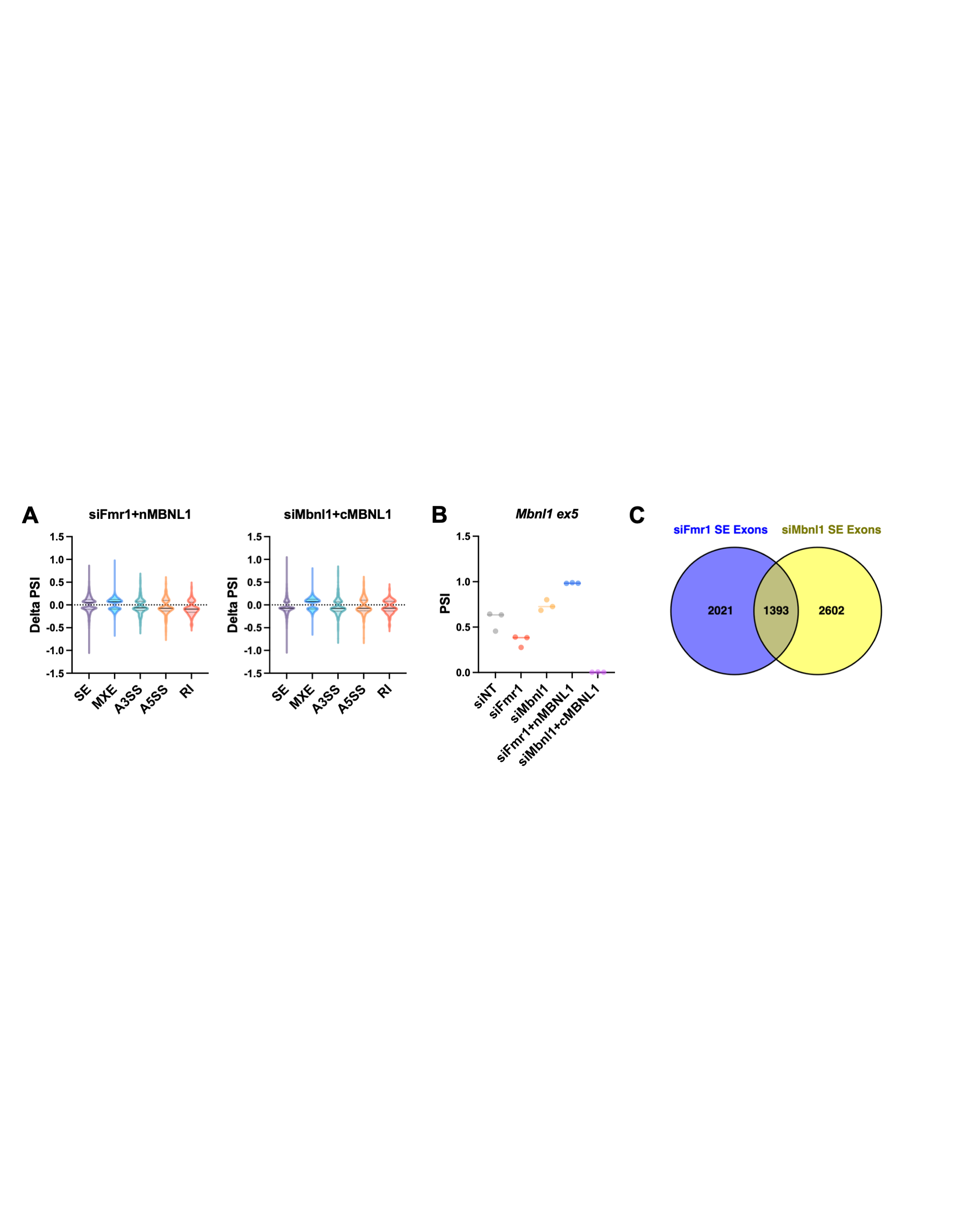

Supplement: S8 Fig — (A) Violin plots showing the distribution of delta PSI in siFmr1+nMBNL1 and siMbnl1+cMBNL1. The solid line is the median and the dashed lines are quartiles. P-value < 0.05, |delta PSI| > 0.05. (B) PSI of Mbnl1 exon 5 in all experimental groups. (C) Venn diagram comparing skipped exon events in siFmr1 and siMbnl1 cells. The underlying data can be found in S3 Data. (TIF) [file pbio.3002417.s008.tif]

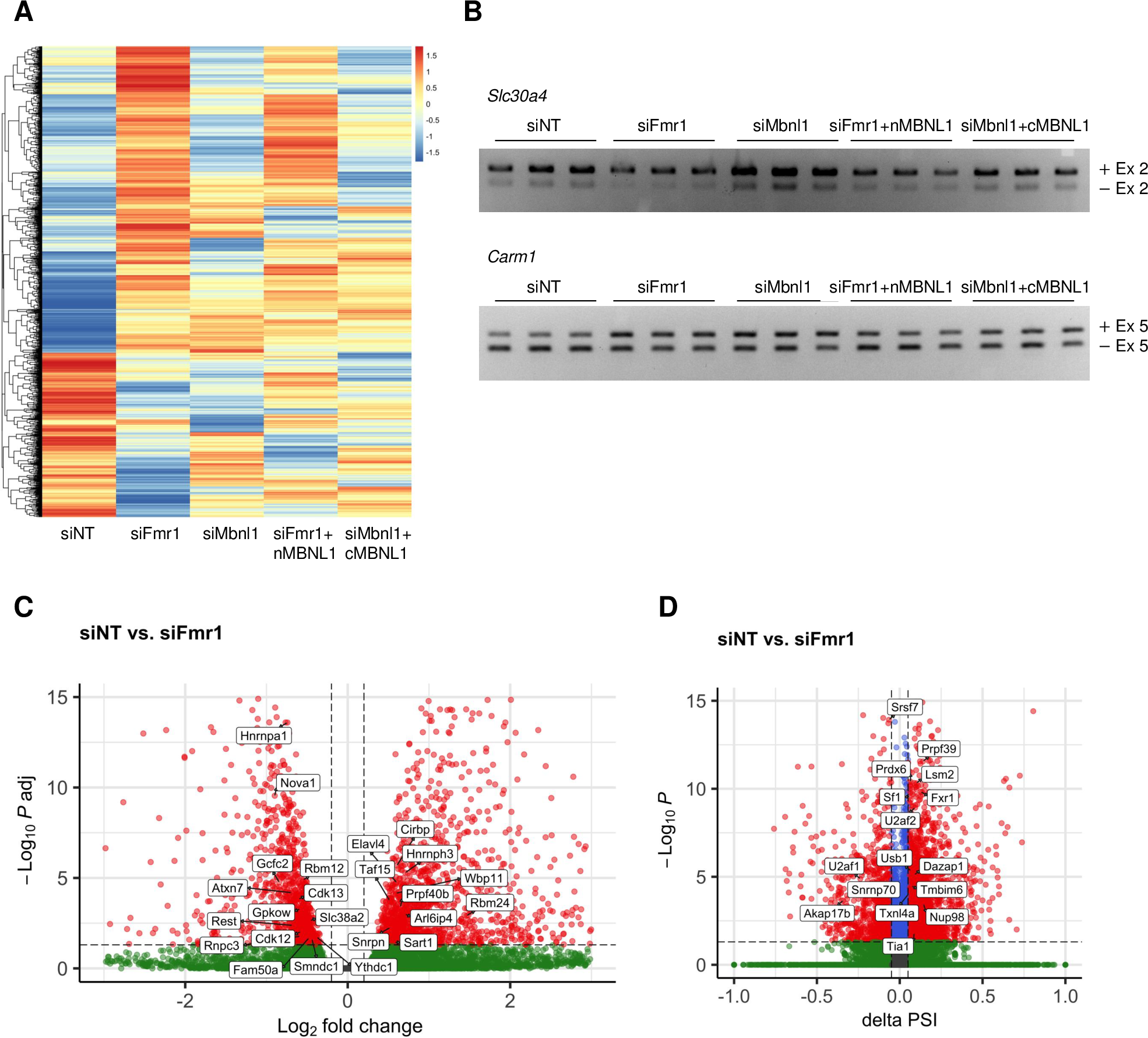

Supplement: S9 Fig — (A) Heatmap visualizing the PSI values of SEs in all groups that, when compared to controls, pass a threshold for significant change upon Fmr1 depletion (P-value < 0.05, |delta PSI| > 0.05). PSI values were adjusted by z-score. (B) RT-PCR for Slc30a4 exon 2 and Carm1 exon 5 splicing. (C) Volcano plots showing the differential expression of RNAs associated with RNA splicing upon Fmr1 depletion. Log2FC > 0.2 or < −0.2, FDR < 0.05, n = 3. (D) Volcano plots showing the AS events involved in RNA splicing upon Fmr1 depletion. P-value < 0.05, |delta PSI| > 0.05. (TIF) [file pbio.3002417.s009.tif]
