# Supplementary material for: FMRP deficiency leads to multifactorial dysregulation of splicing and mislocalization of MBNL1 to the cytoplasm
Source: PLoS Biol. 2023 Dec 4;21(12):e3002417. doi: 10.1371/journal.pbio.3002417 (PMC10721184; doi:10.1371/journal.pbio.3002417)
Supplement: S1 Raw Images — (PDF) [file pbio.3002417.s013.pdf]

Figure 1G

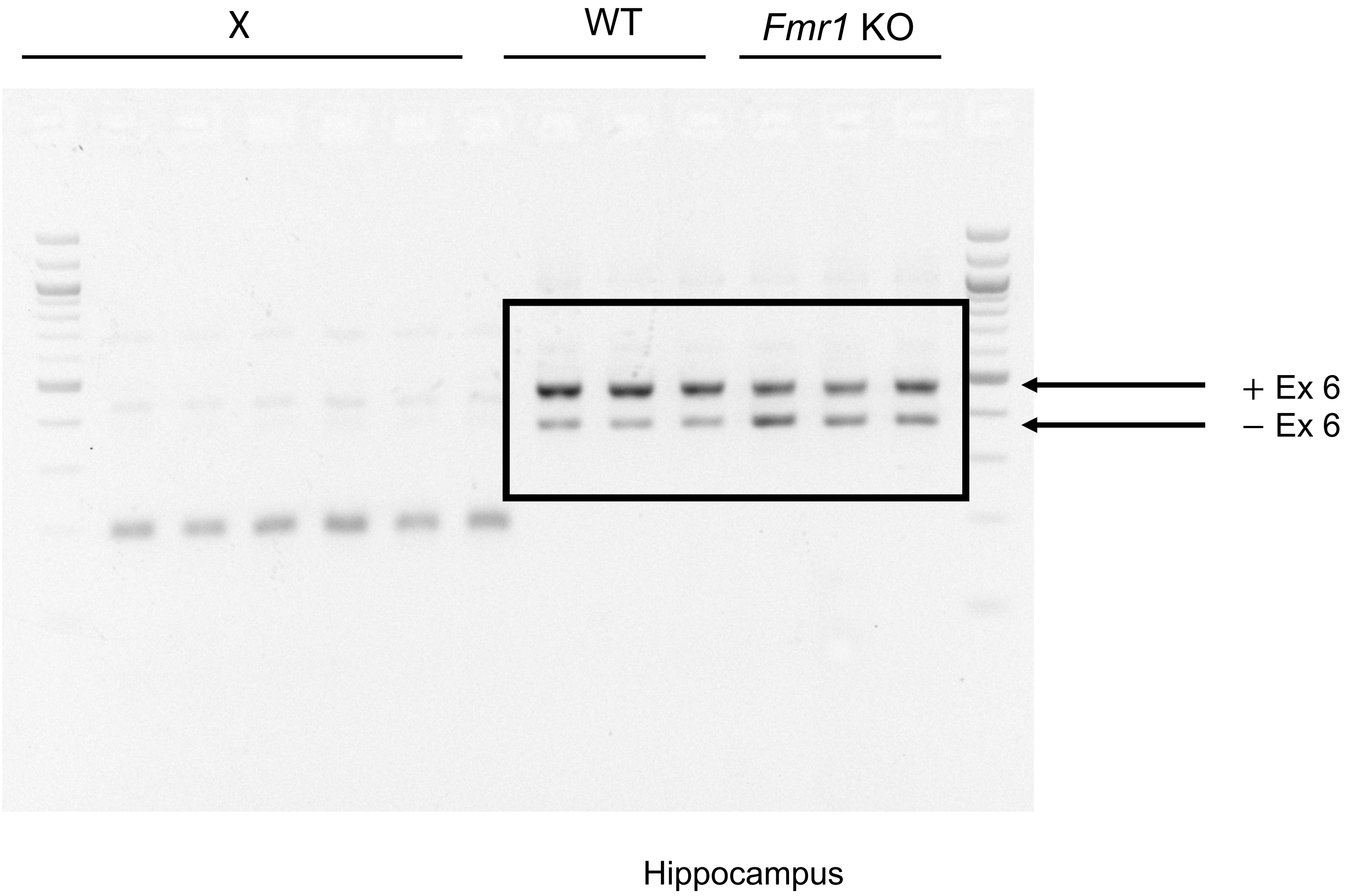

Figure 1G

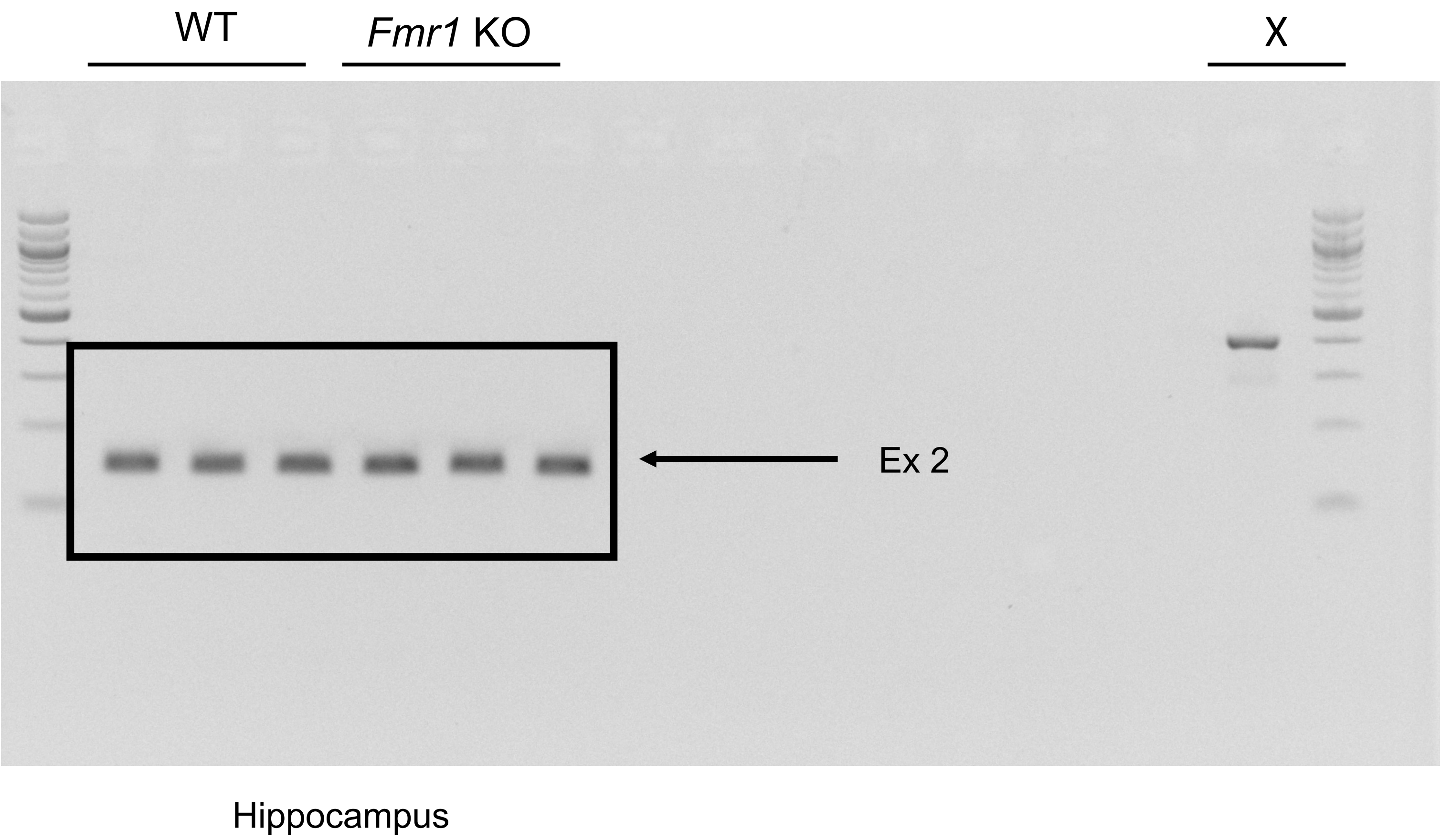

Figure 1G

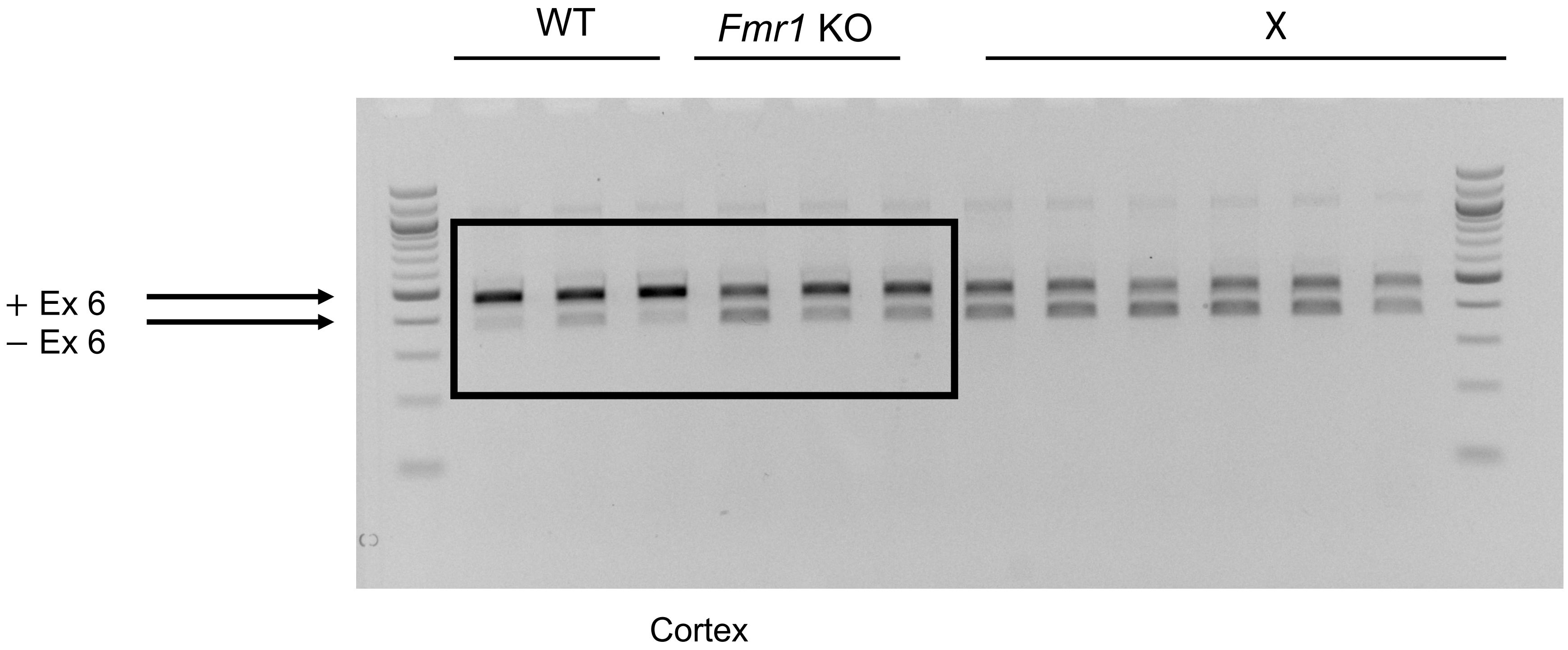

Figure 1G

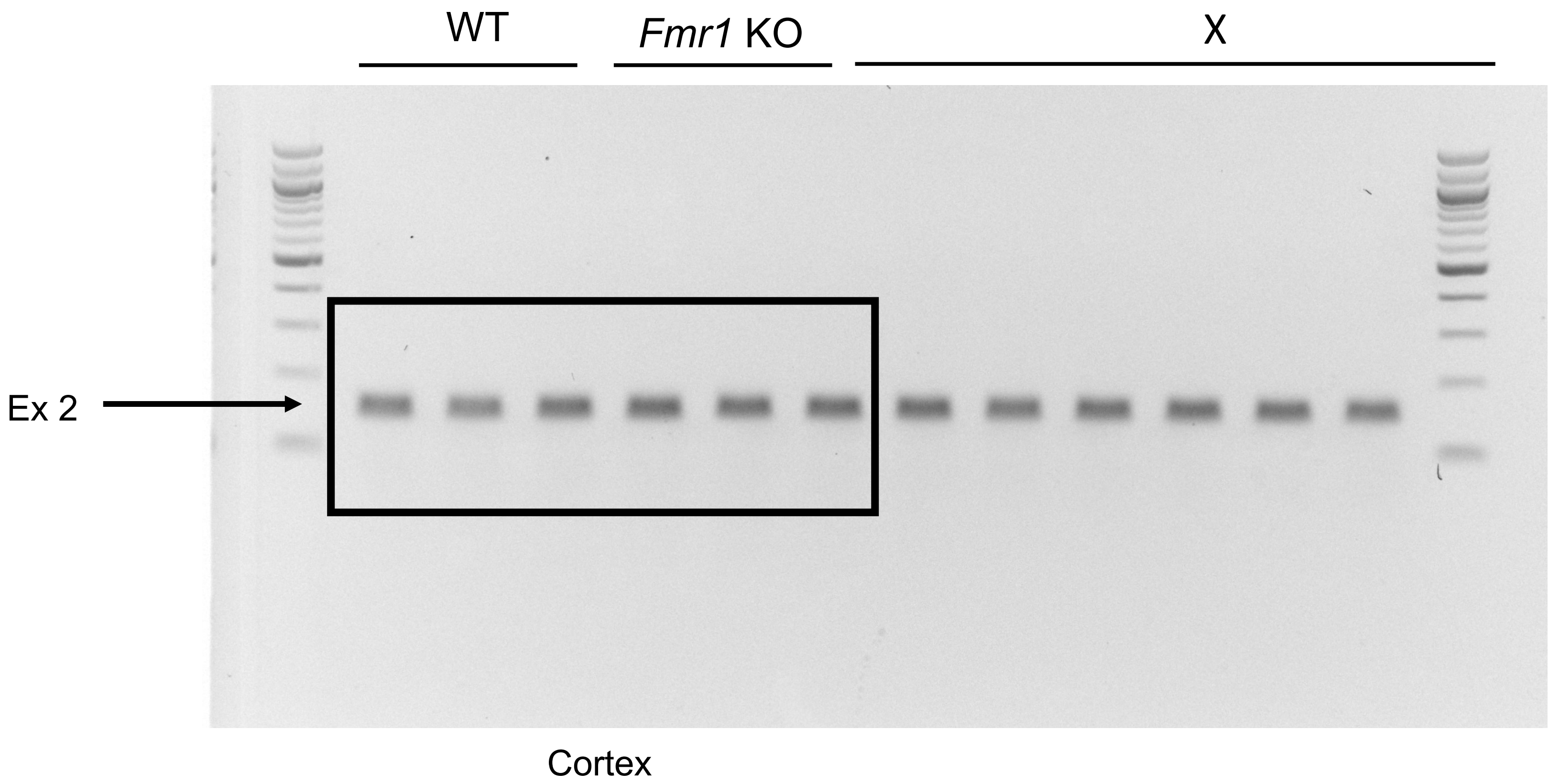

Figure 2A

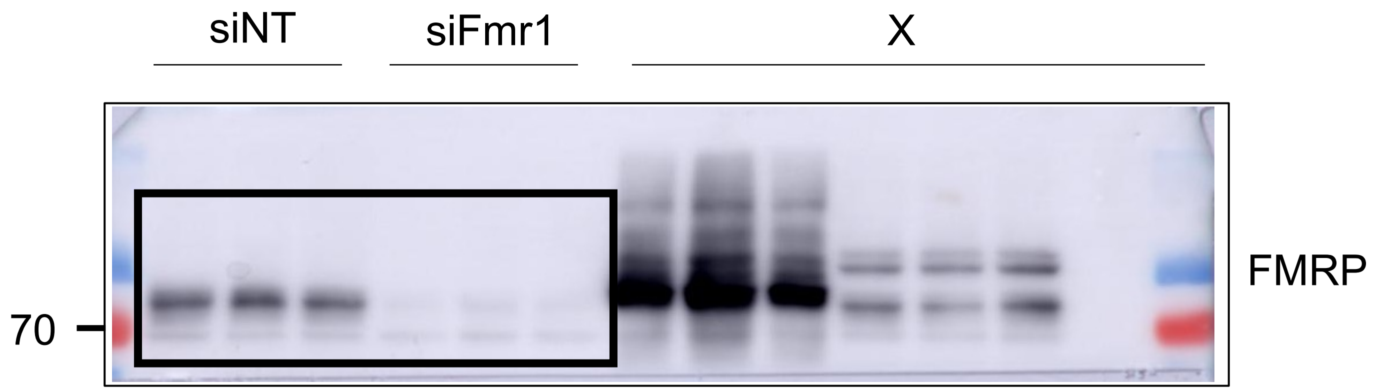

Figure 2A

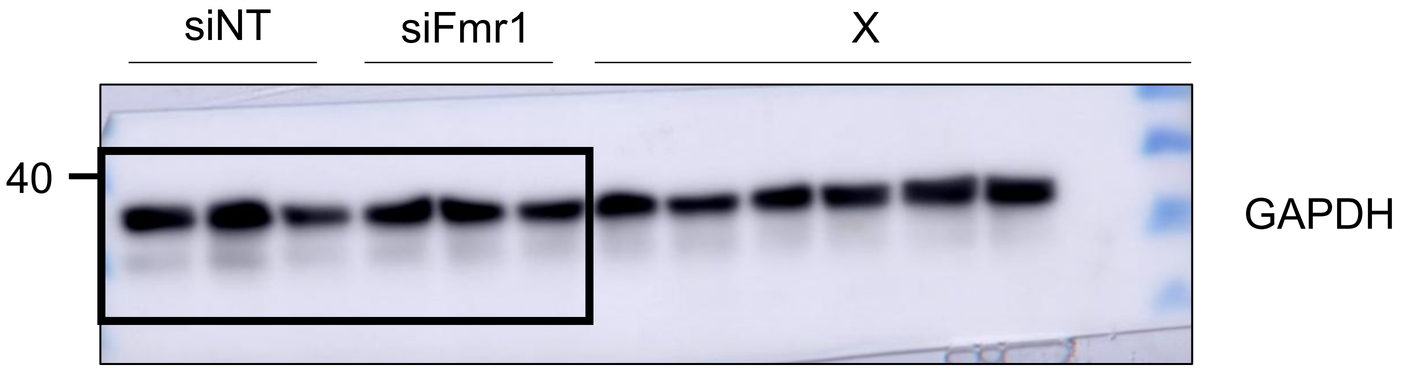

Figure 2E

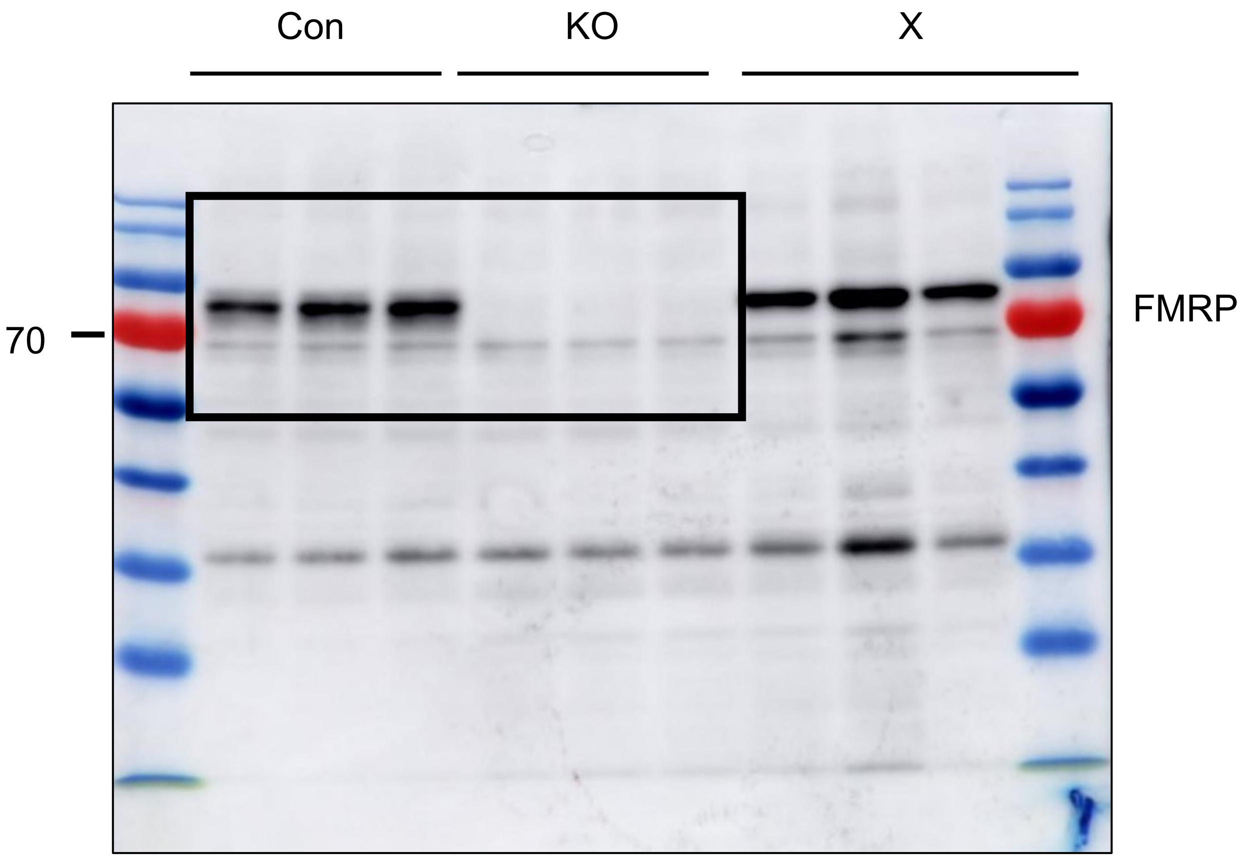

Figure 2E

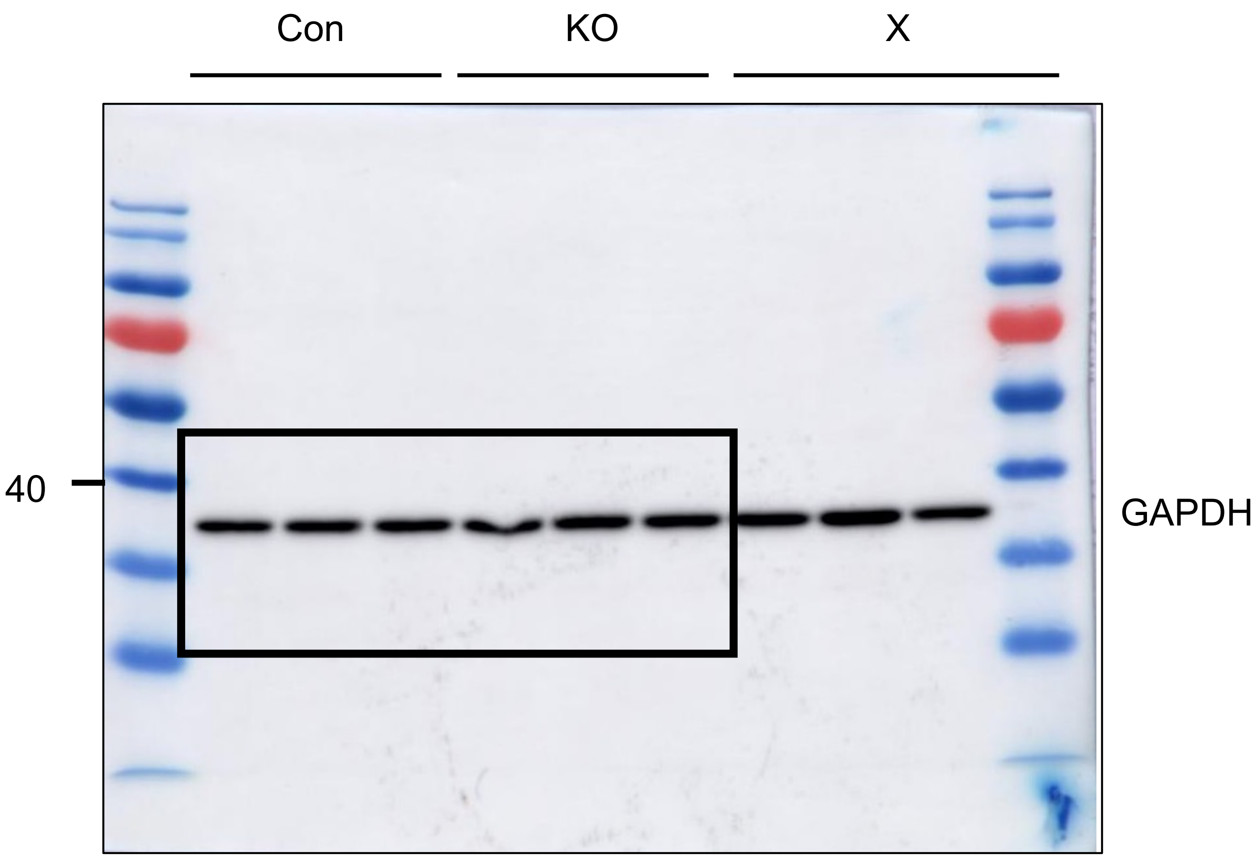

Figure 2H

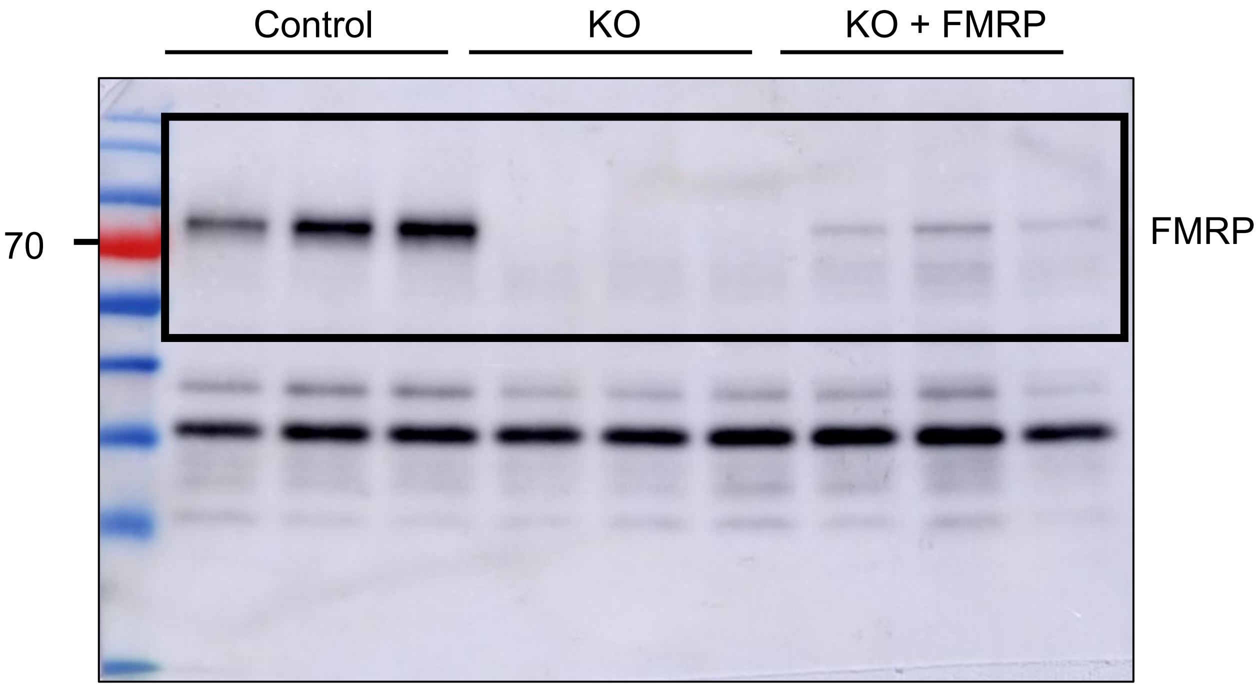

Figure 2H

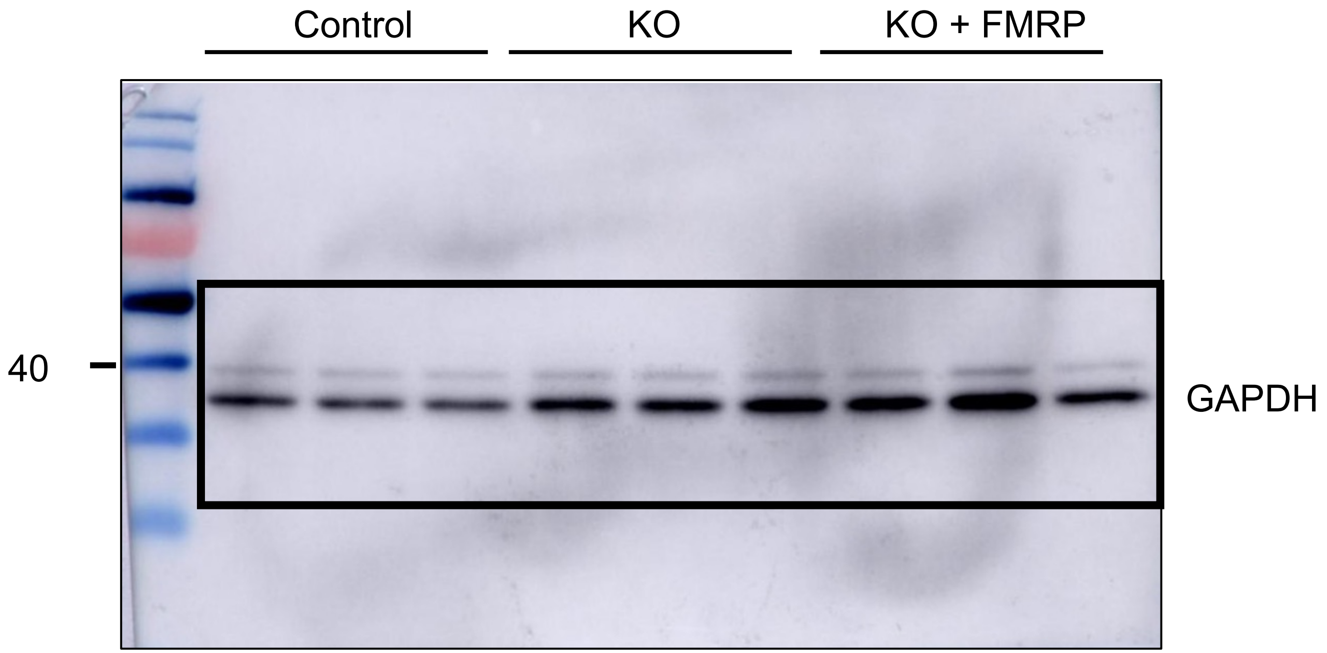

Figure 4B

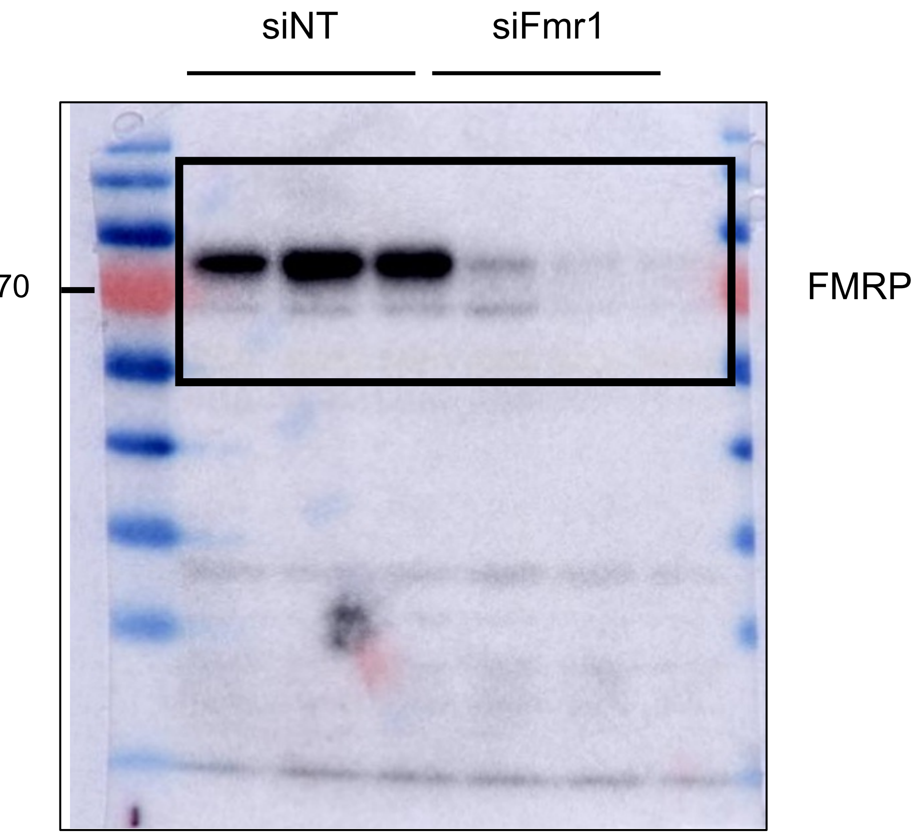

Figure 4B

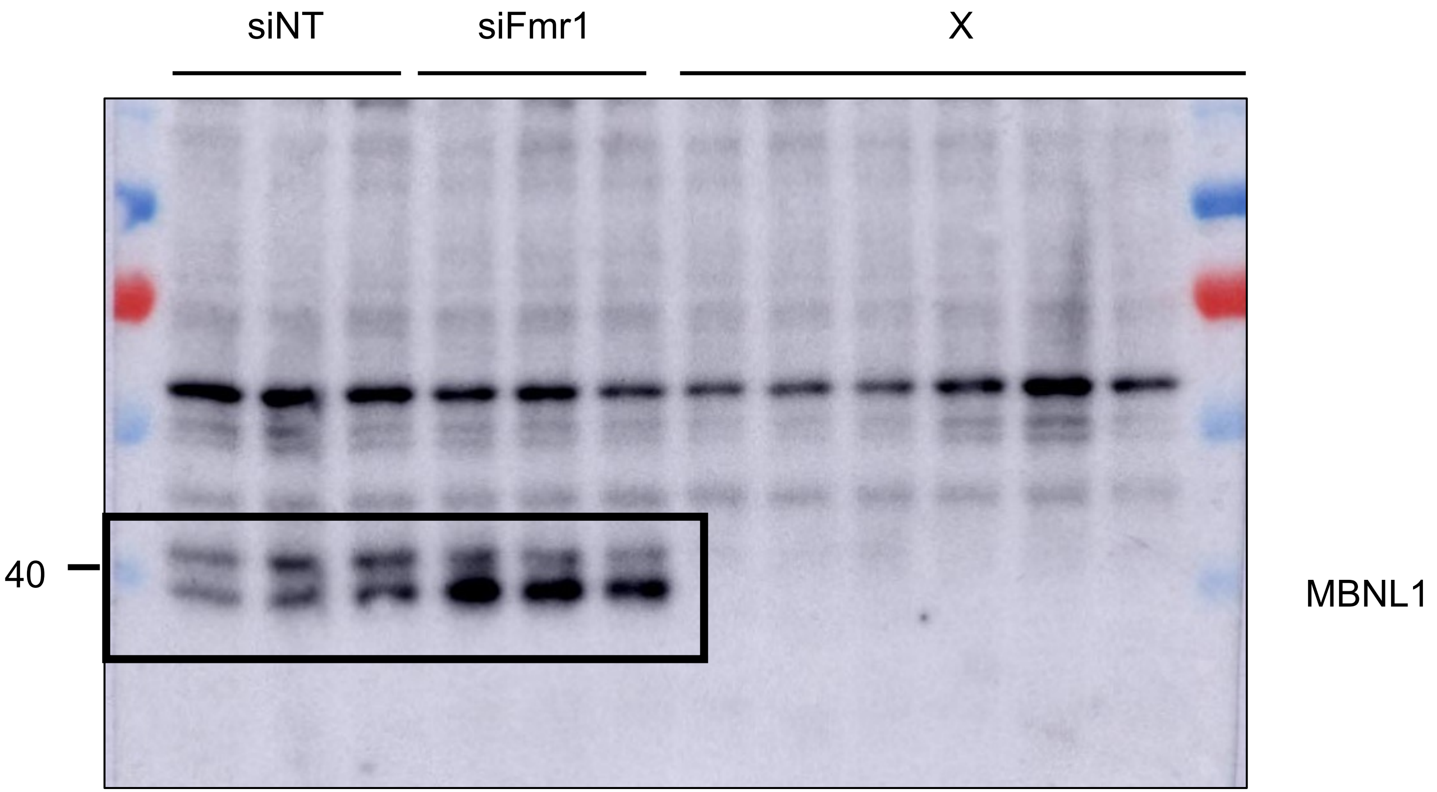

Figure 4B

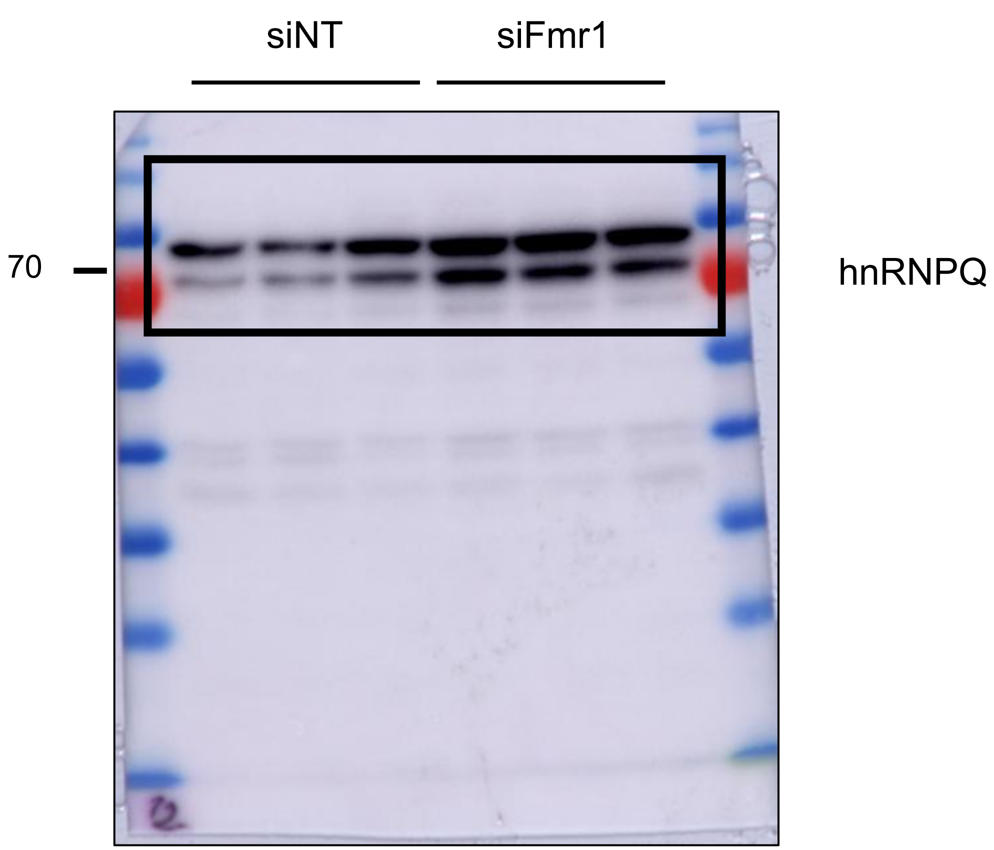

Figure 4B

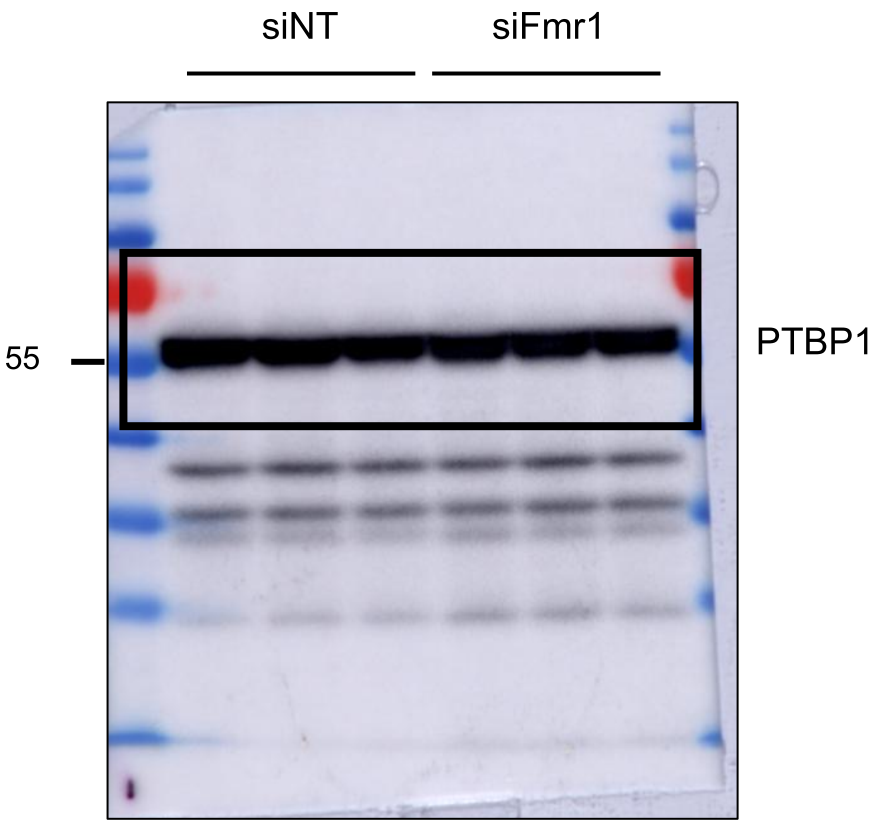

Figure 4B

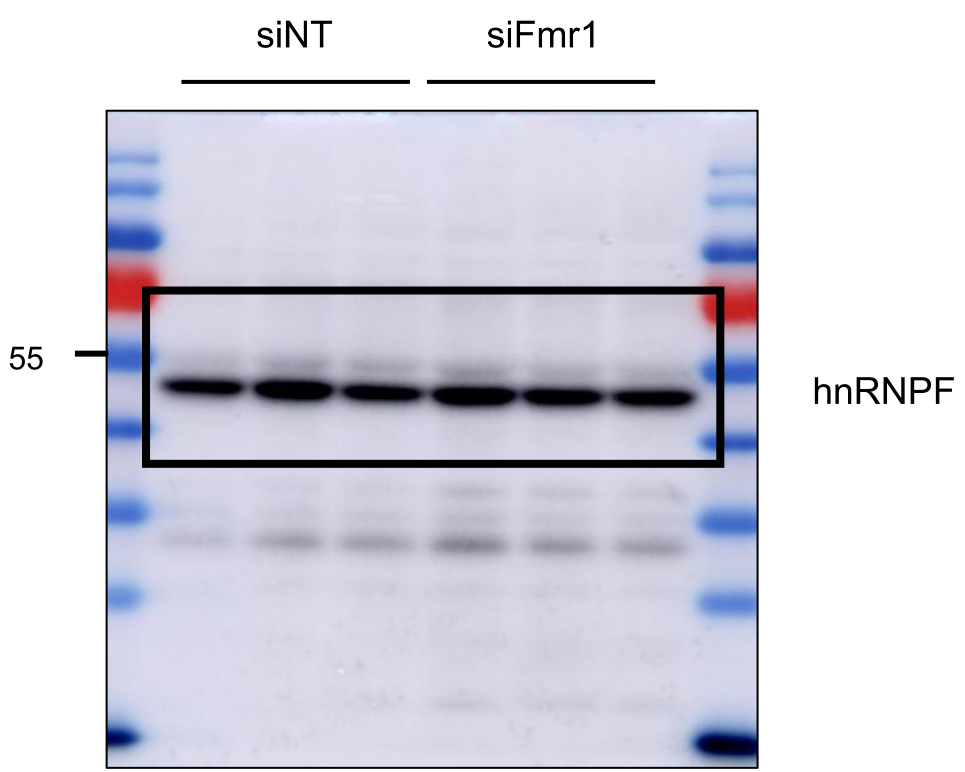

Figure 4B

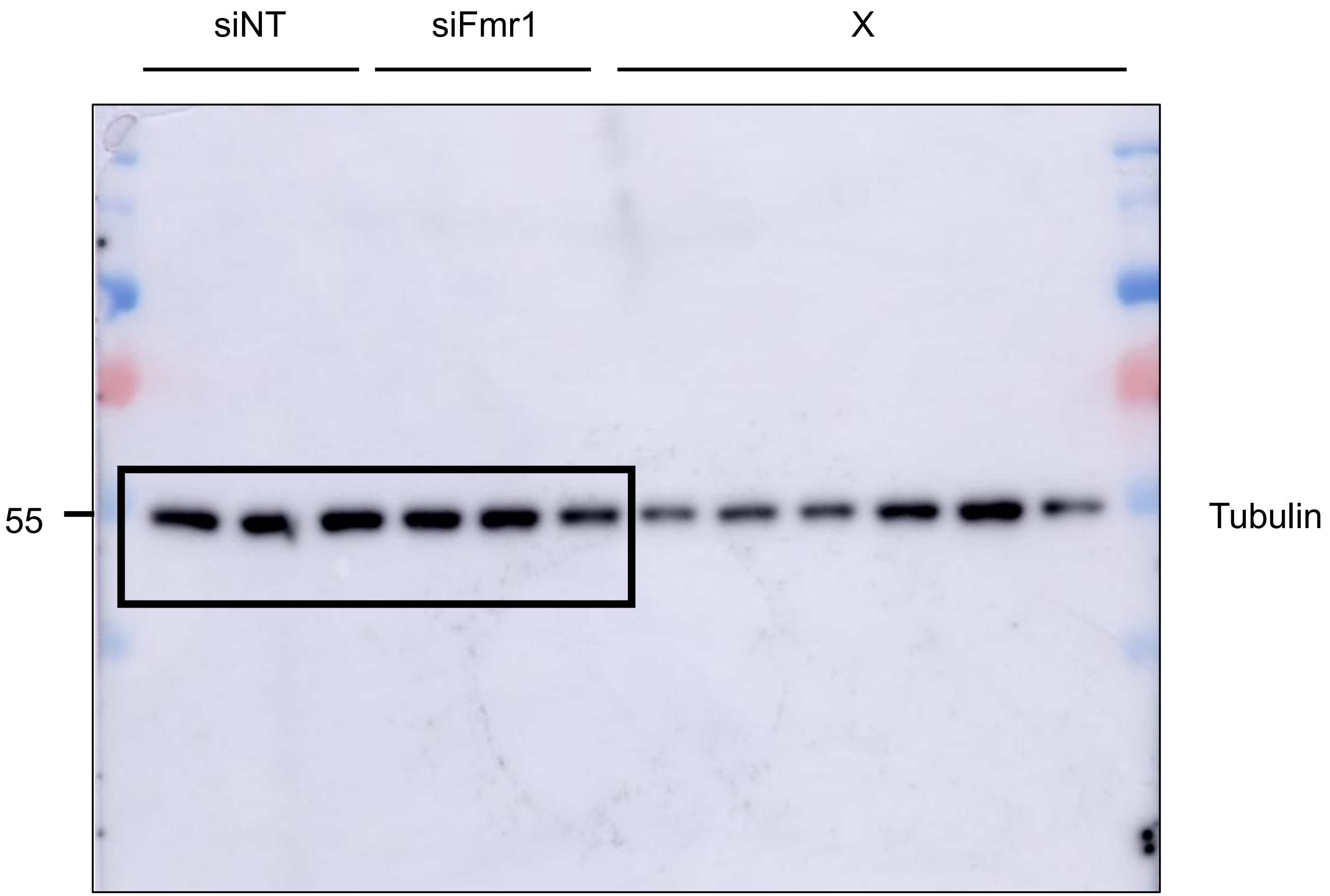

Figure 4B

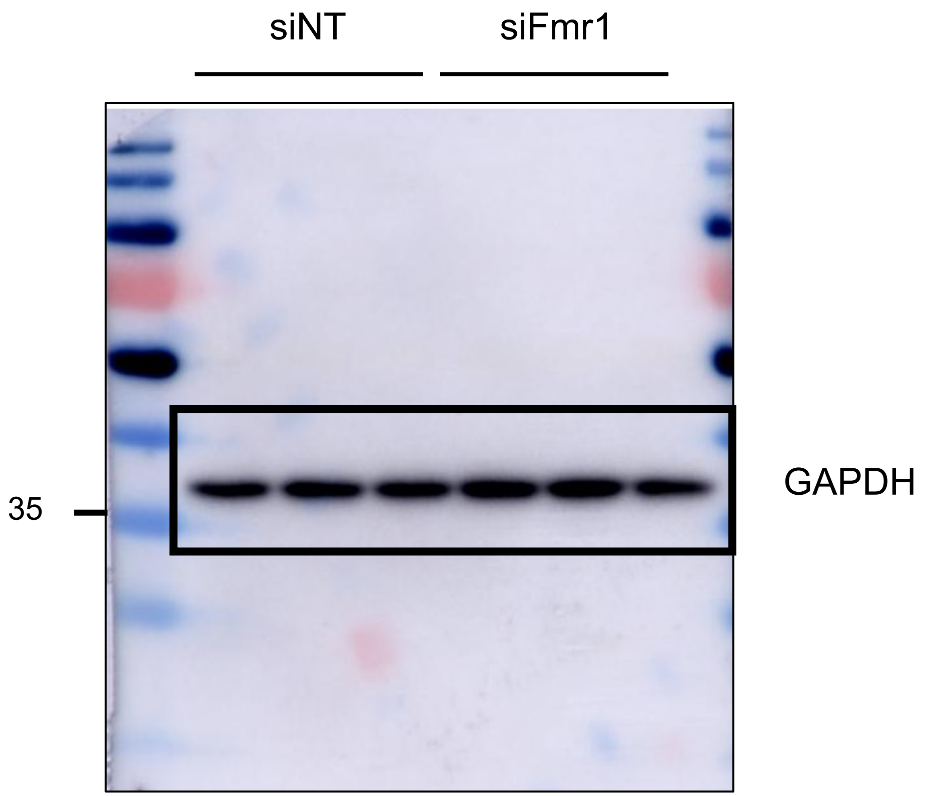

Figure 4B

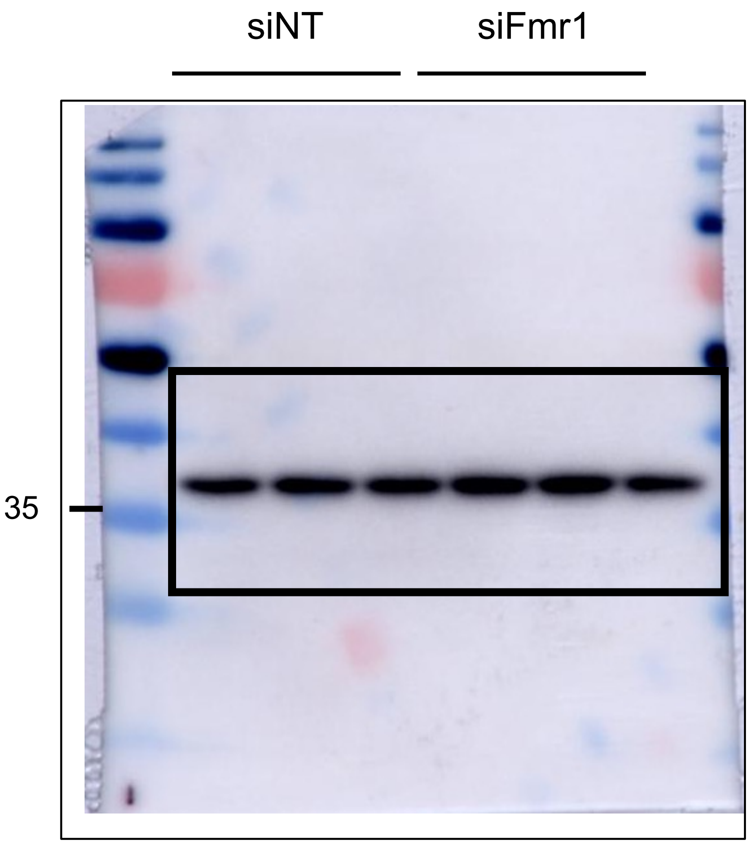

Relevant control GAPDH
for FMRP and PTBP1

Figure 4B

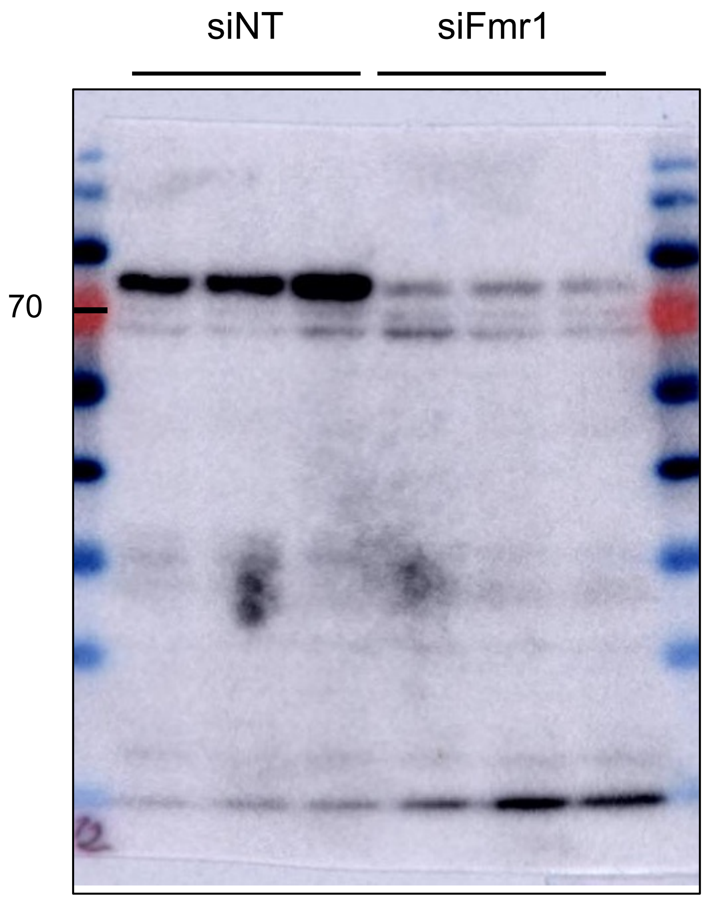

Relevant FMRP for hnRNPQ

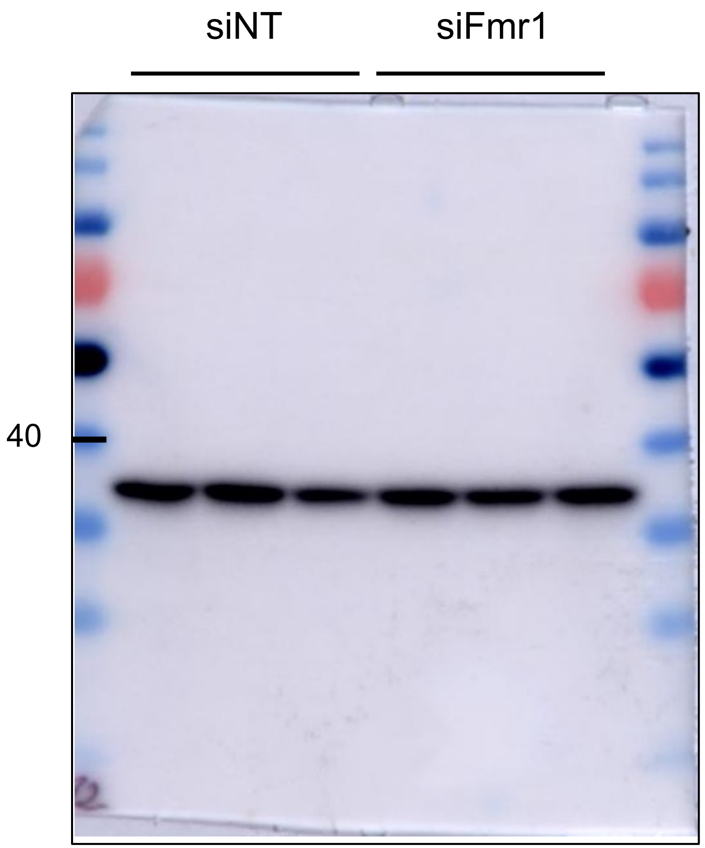

Relevant control GAPDH for hnRNPQ

Figure 4B

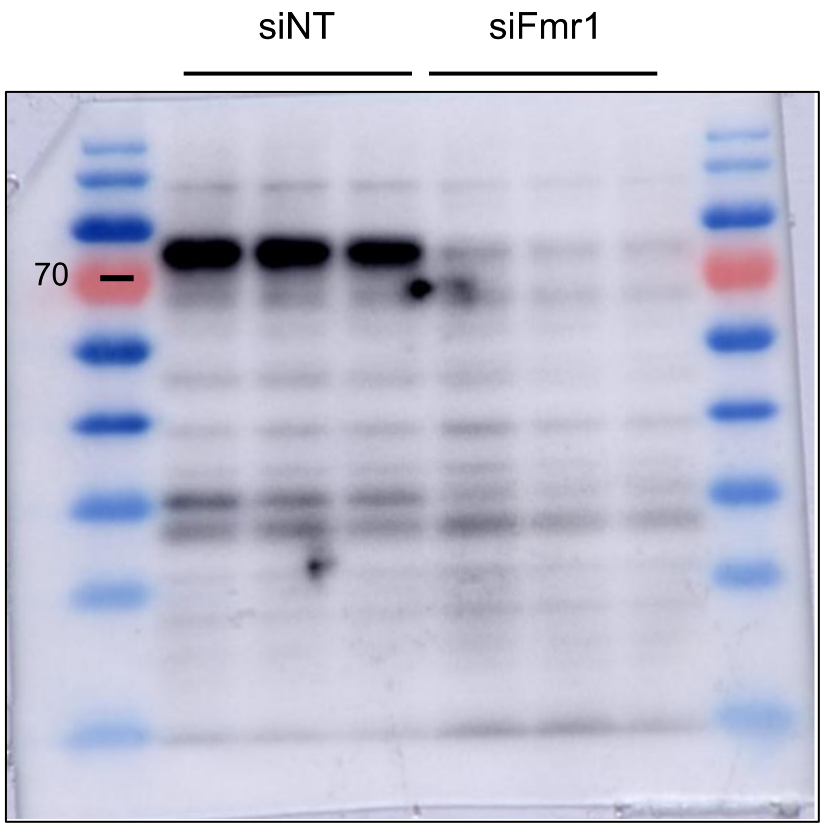

Relevant FMRP for hnRNPF

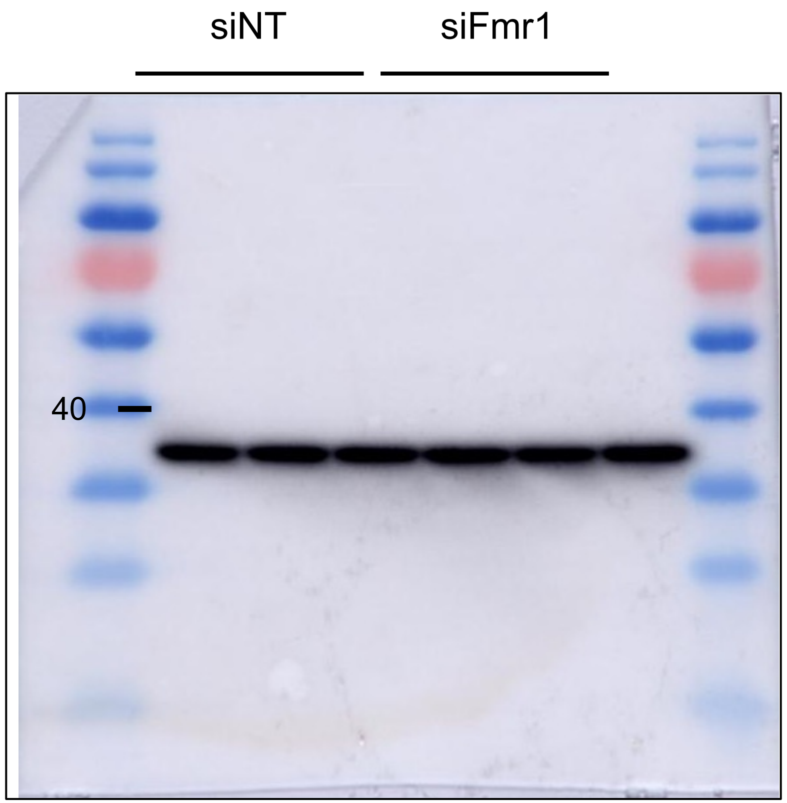

Relevant control GAPDH for hnRNPF

Figure 4C

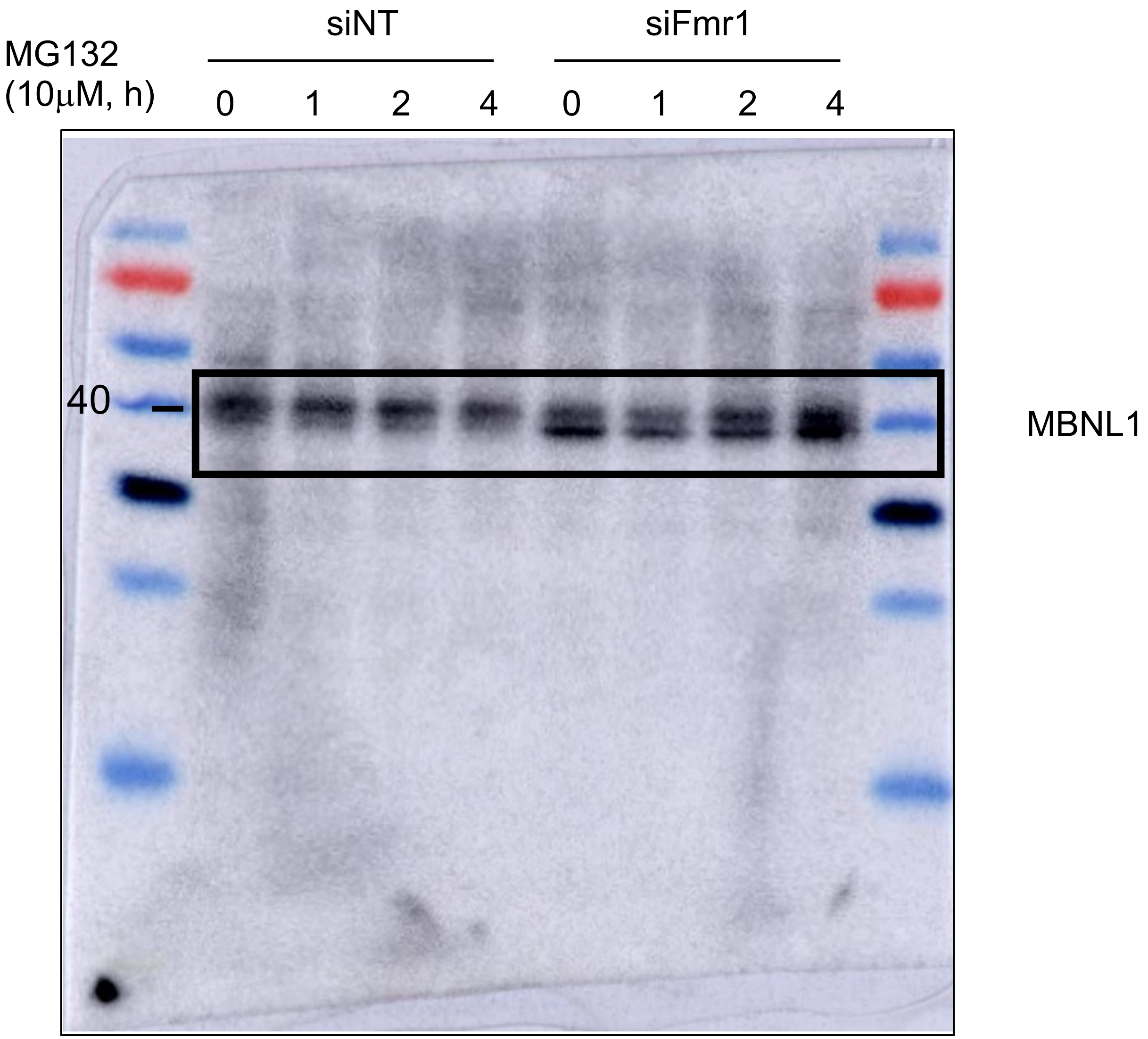

Figure 4C

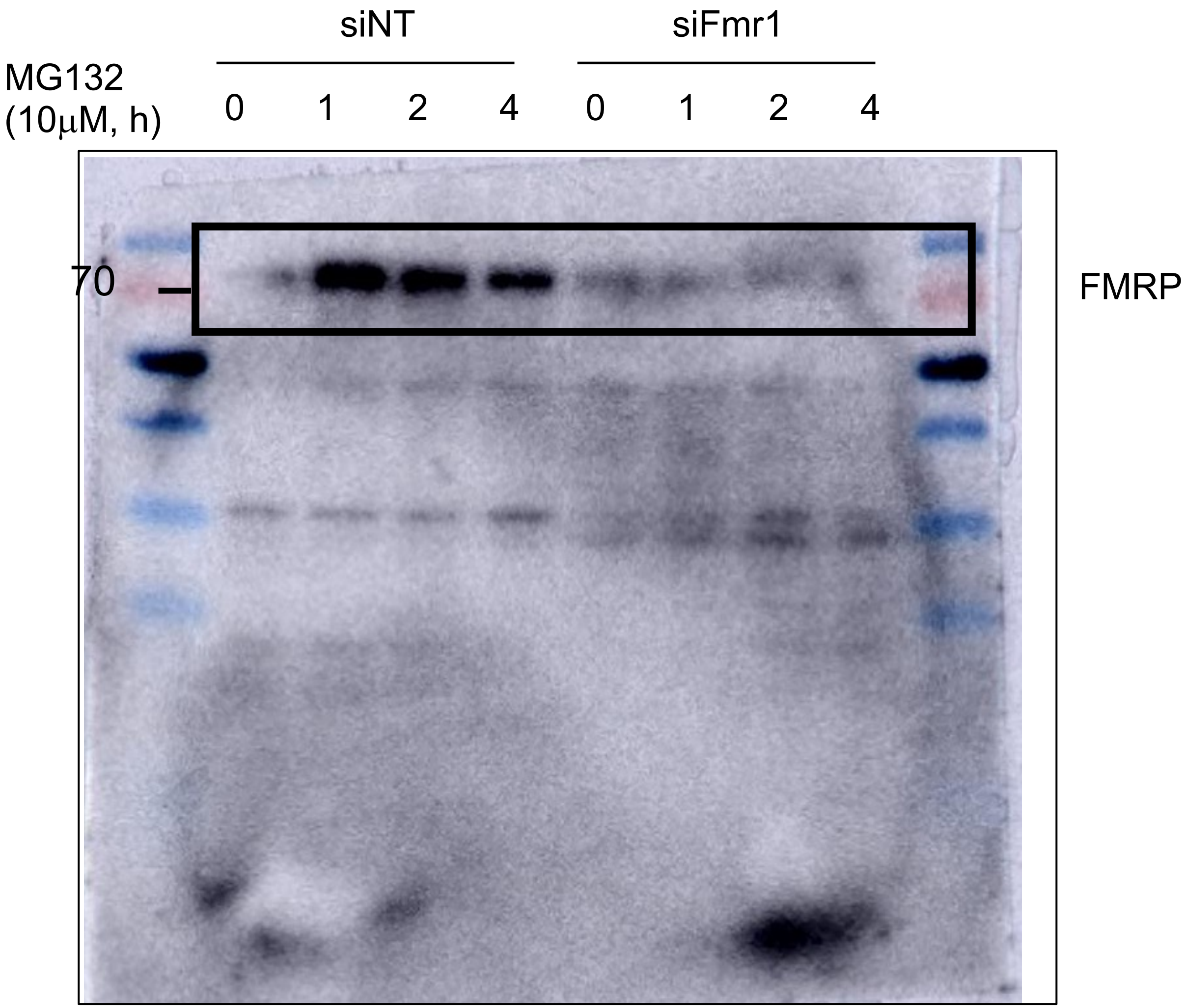

Figure 4C

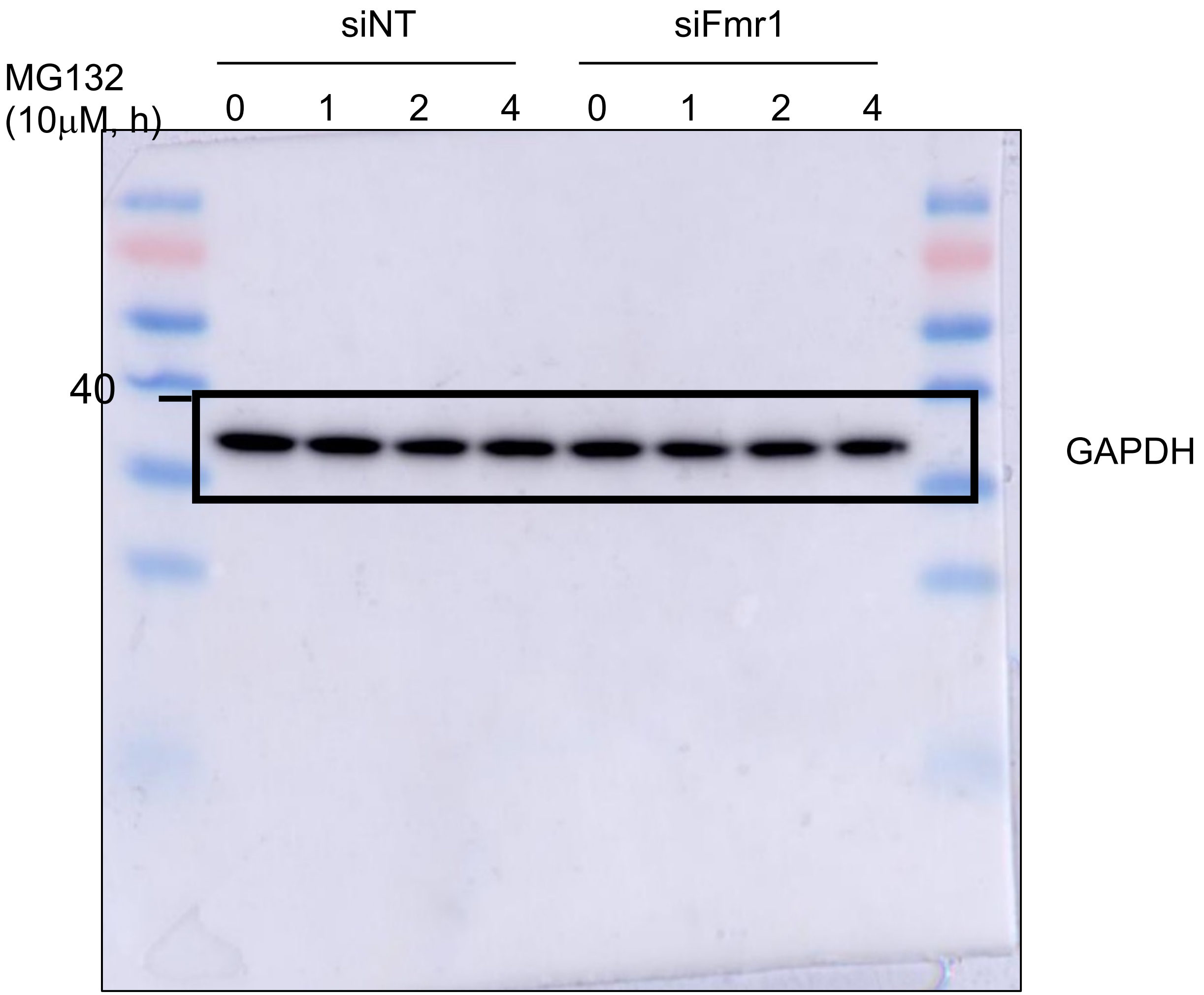

Figure 4C

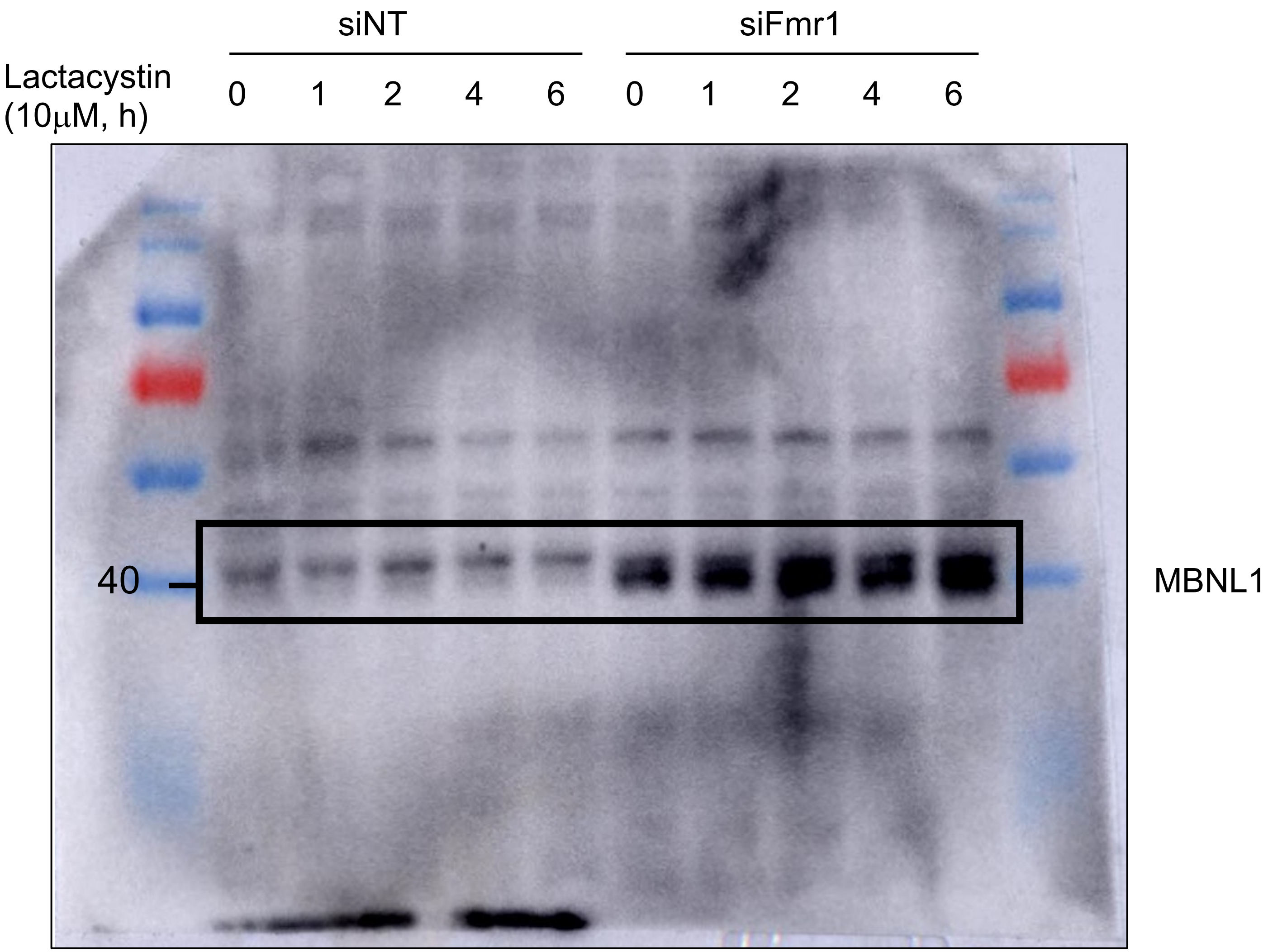

Figure 4C

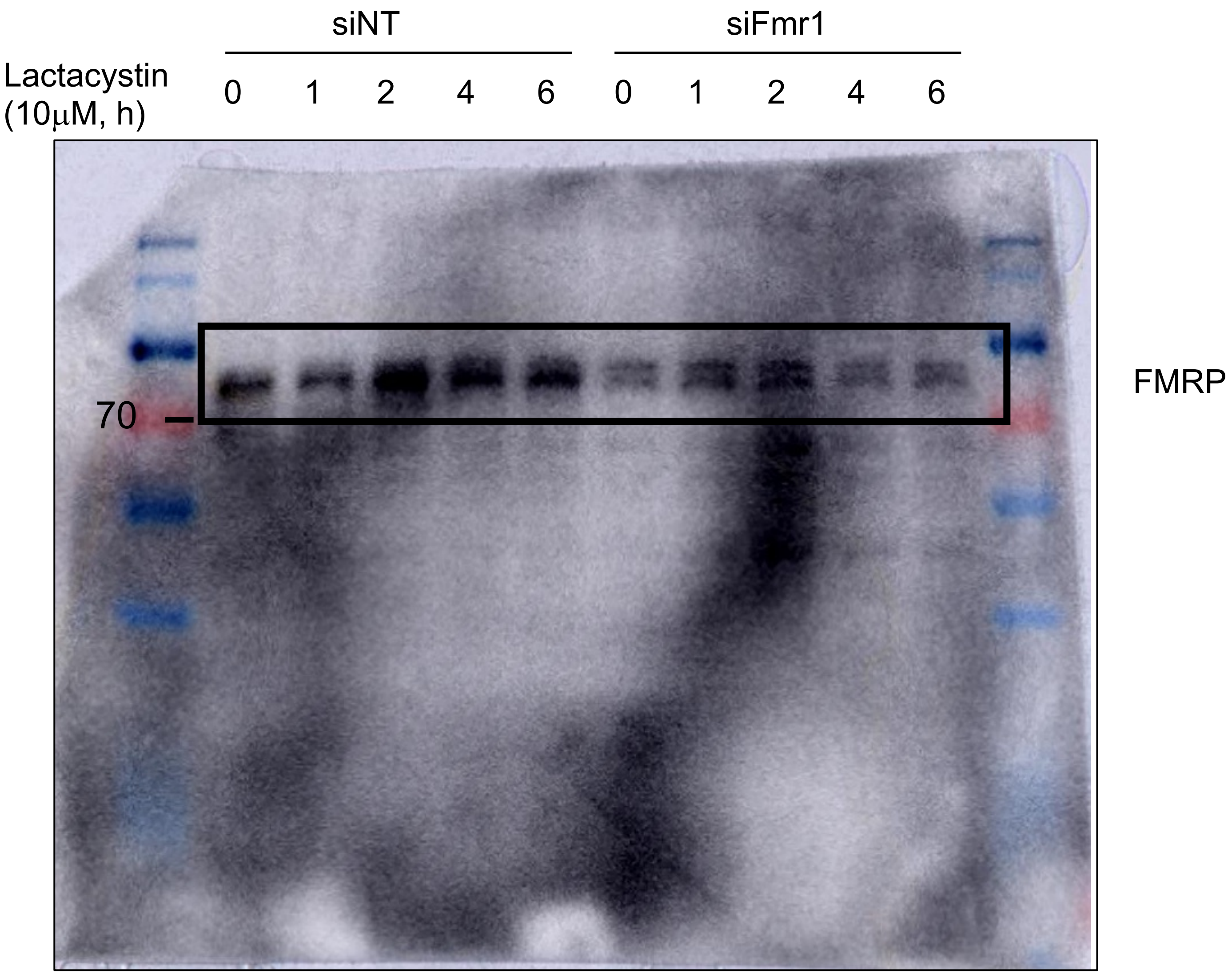

Figure 4C

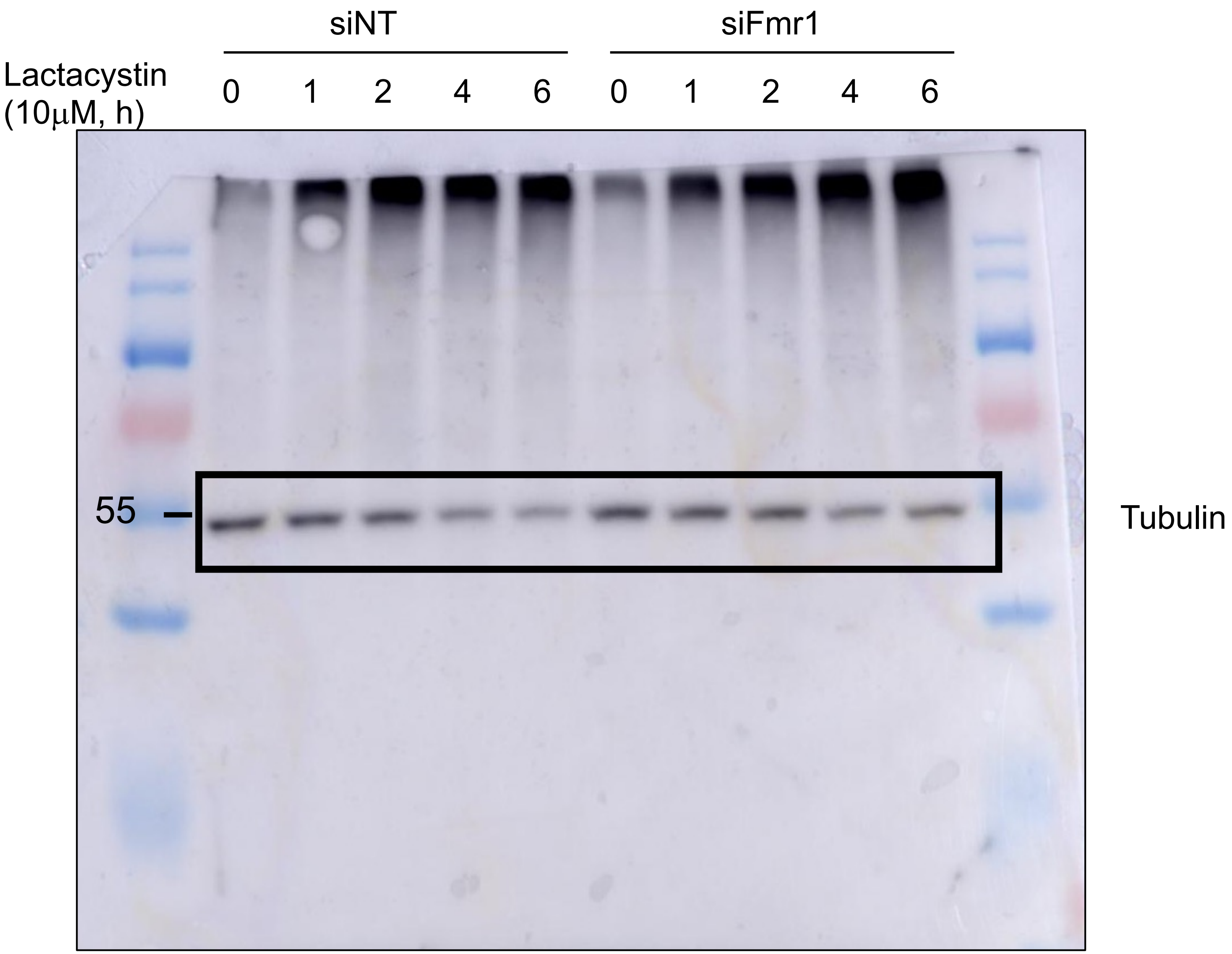

Figure 5B

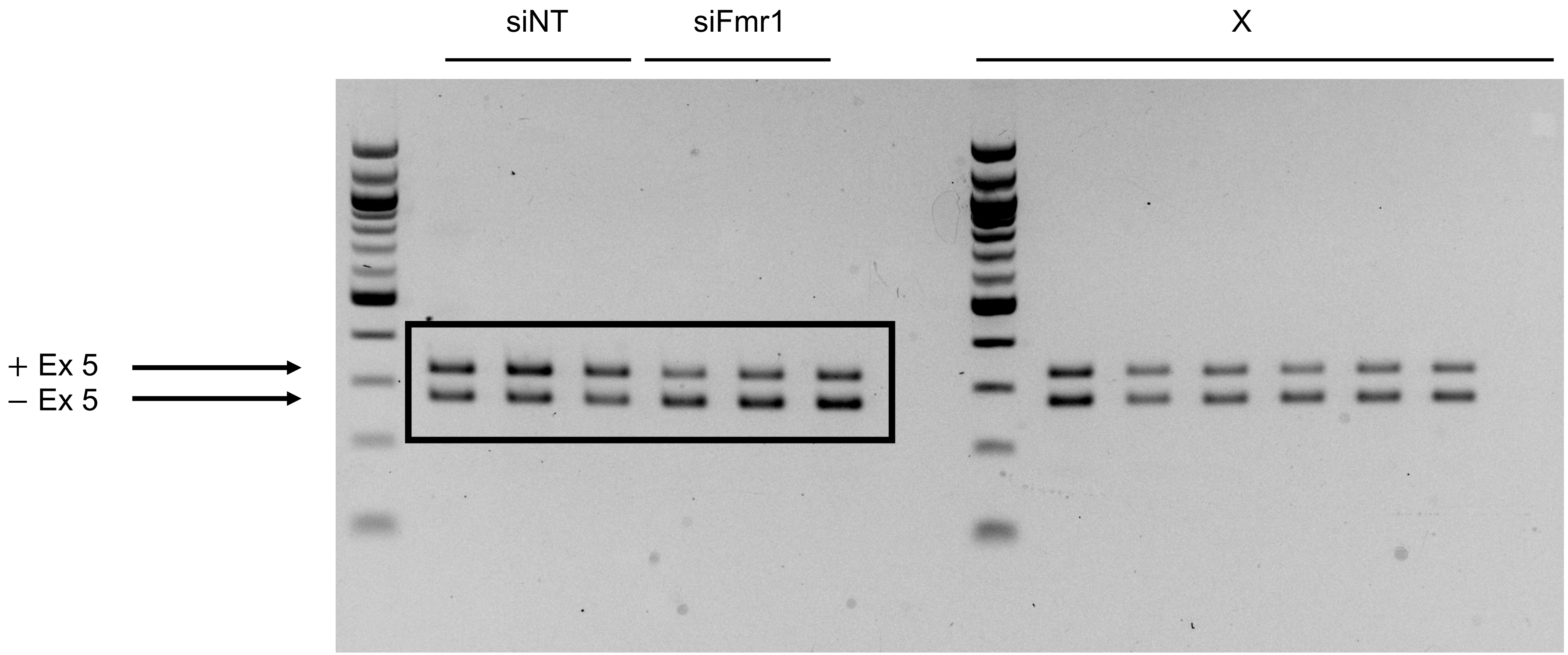

Figure 5B

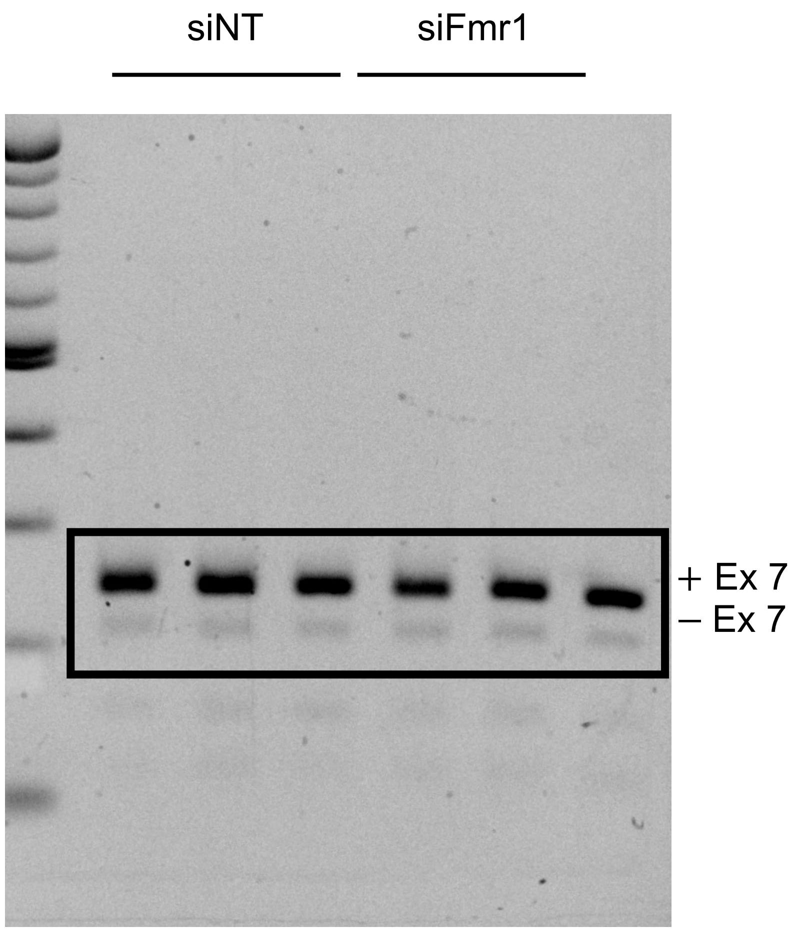

Figure 5B

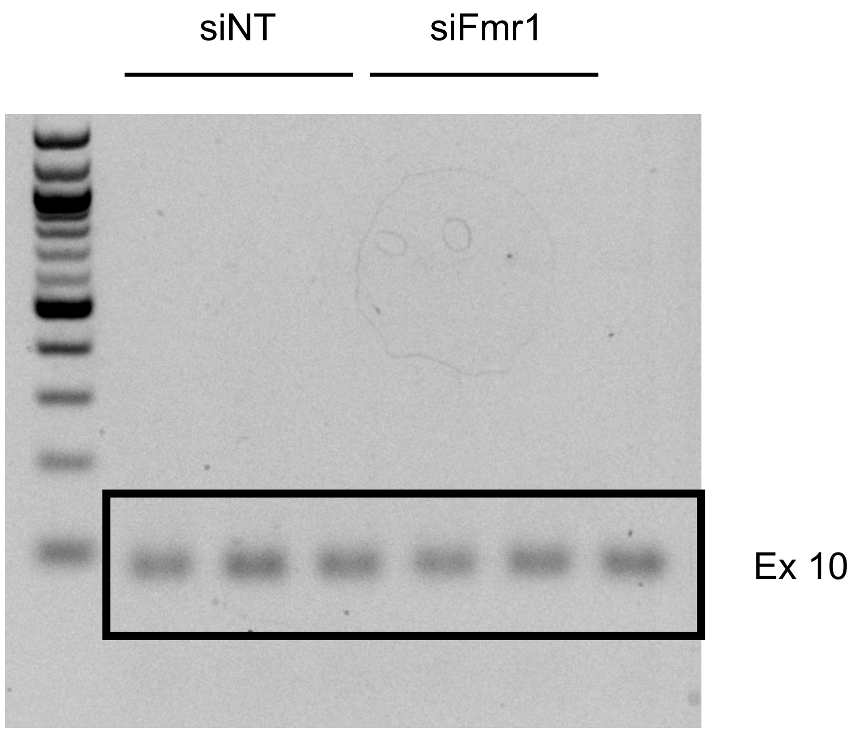

Figure 5C

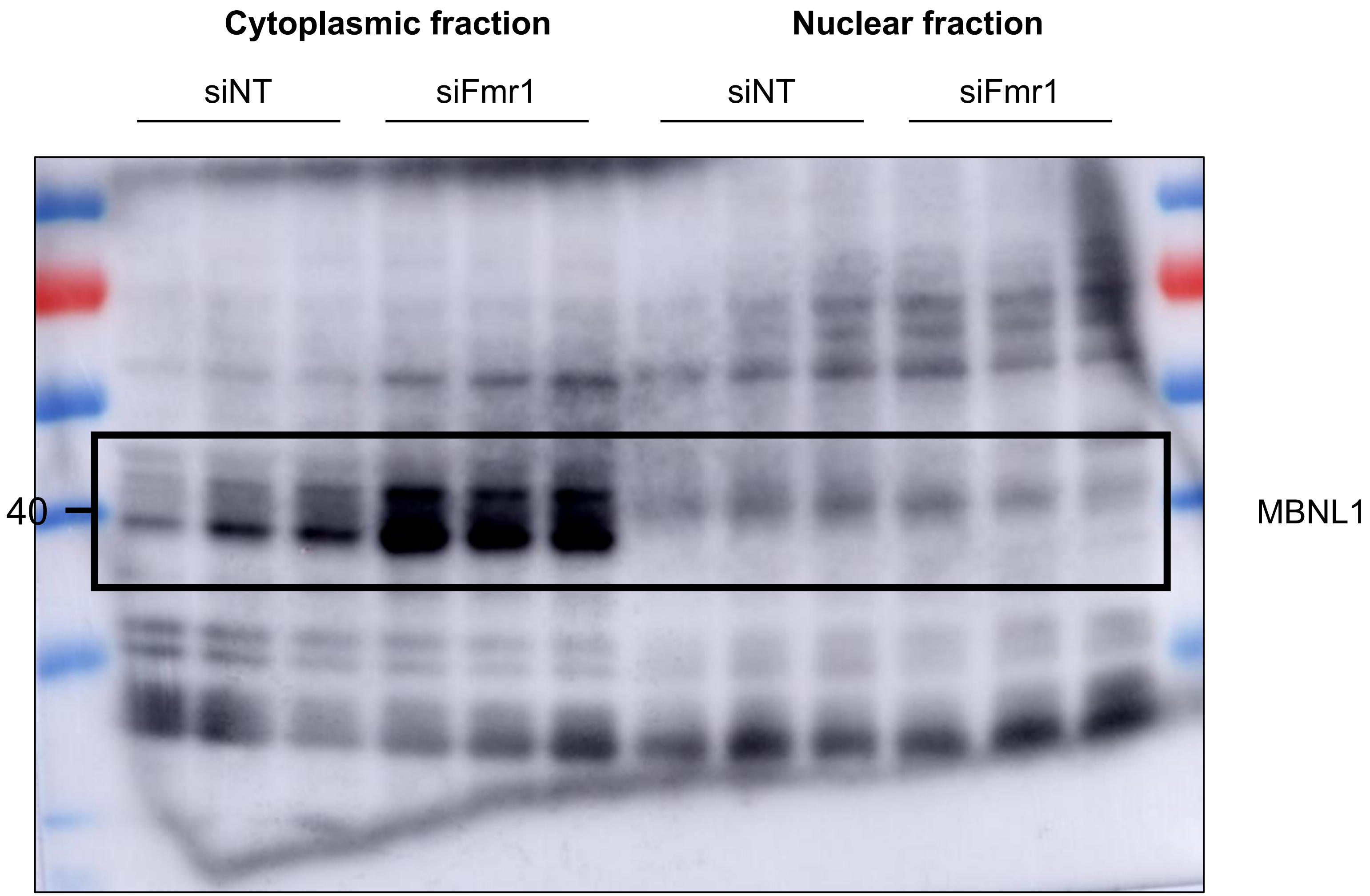

Cytoplasmic fraction
Nuclear fraction
siNT
siFmr1
siNT
siFmr1
40
MBNL1

Figure 5C

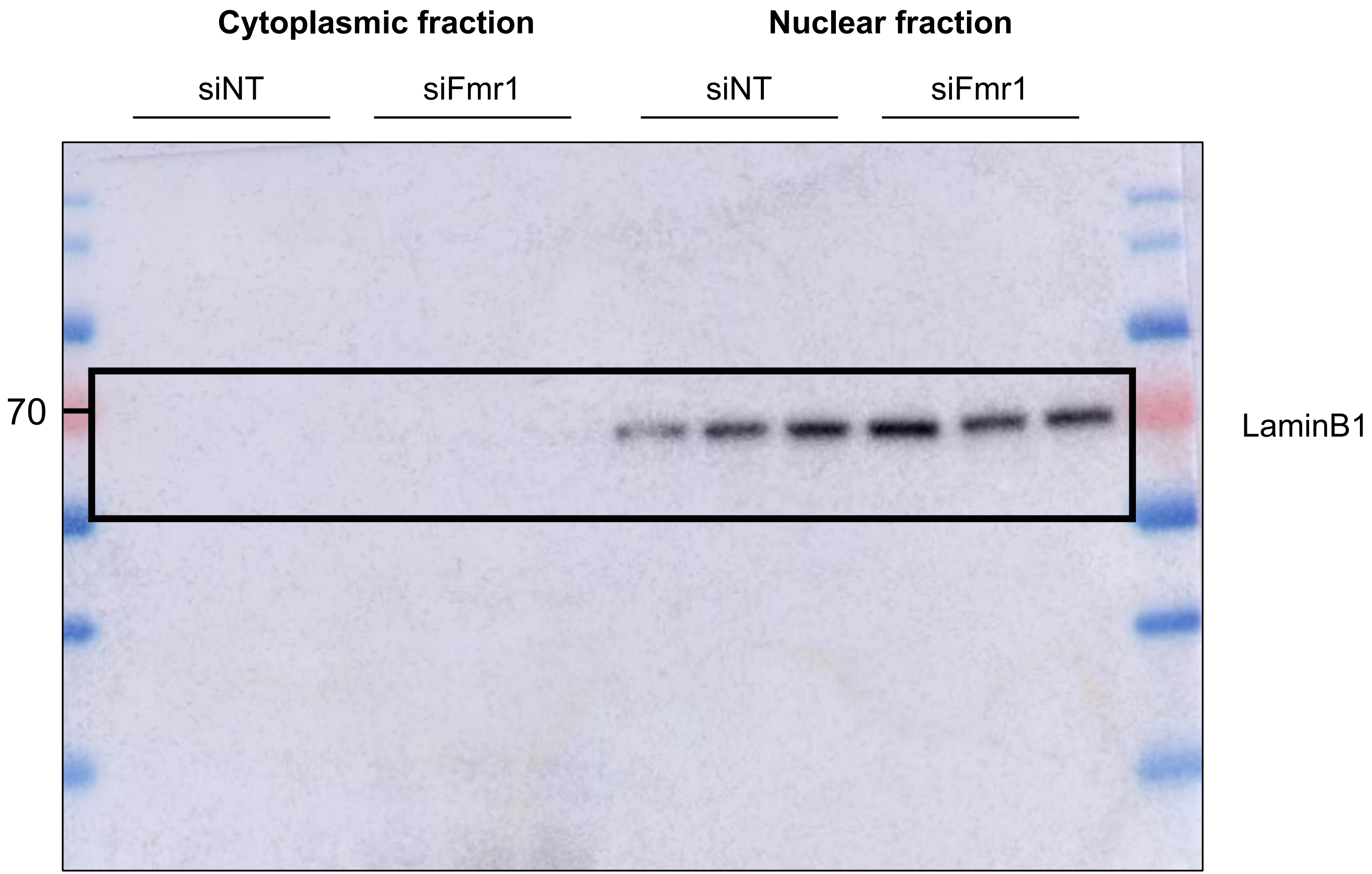

Cytoplasmic fraction
Nuclear fraction
siNT
siFmr1
siNT
siFmr1
70
LaminB1

Figure 5C

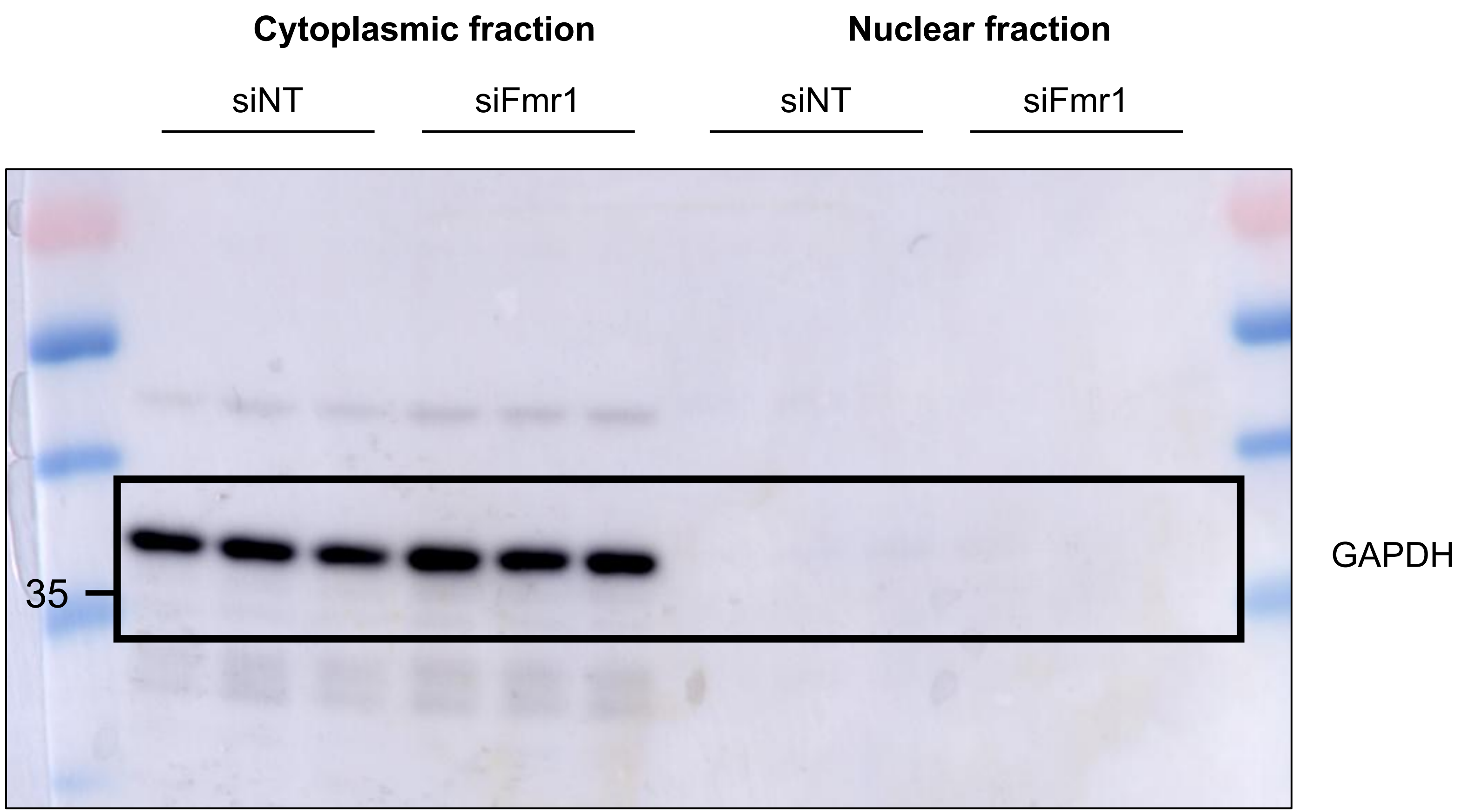
Cytoplasmic fraction
Nuclear fraction
siNT
siFmr1
siNT
siFmr1
35
GAPDH

Figure 7B

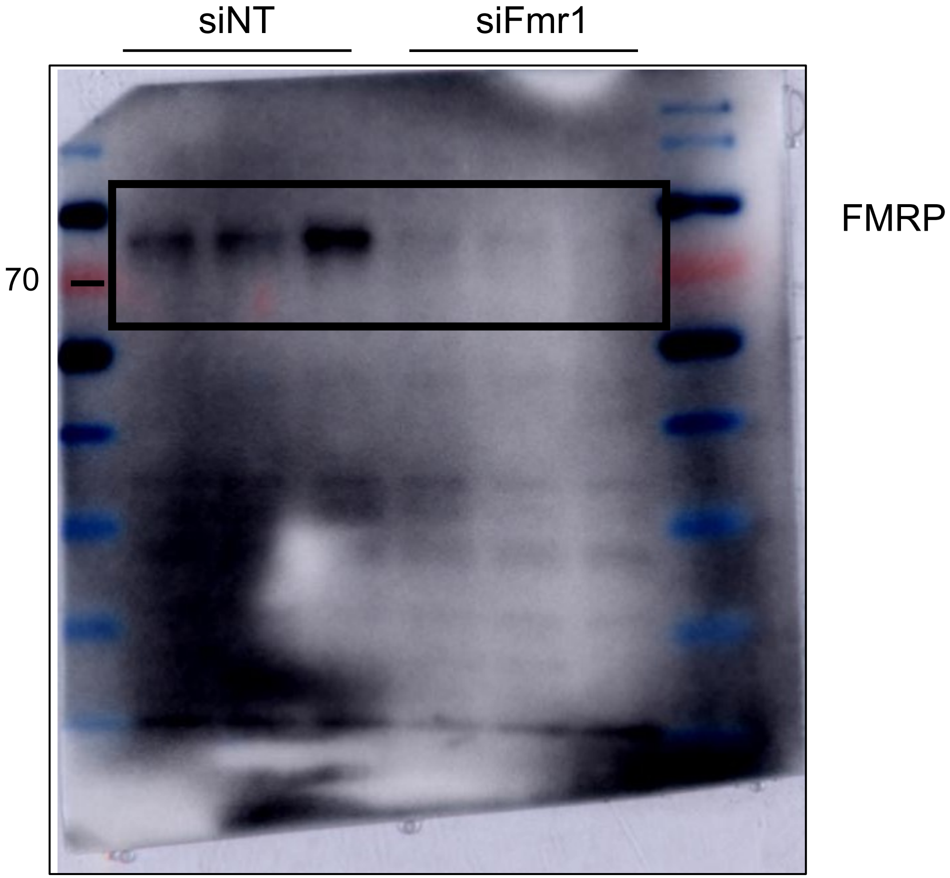

Figure 7B

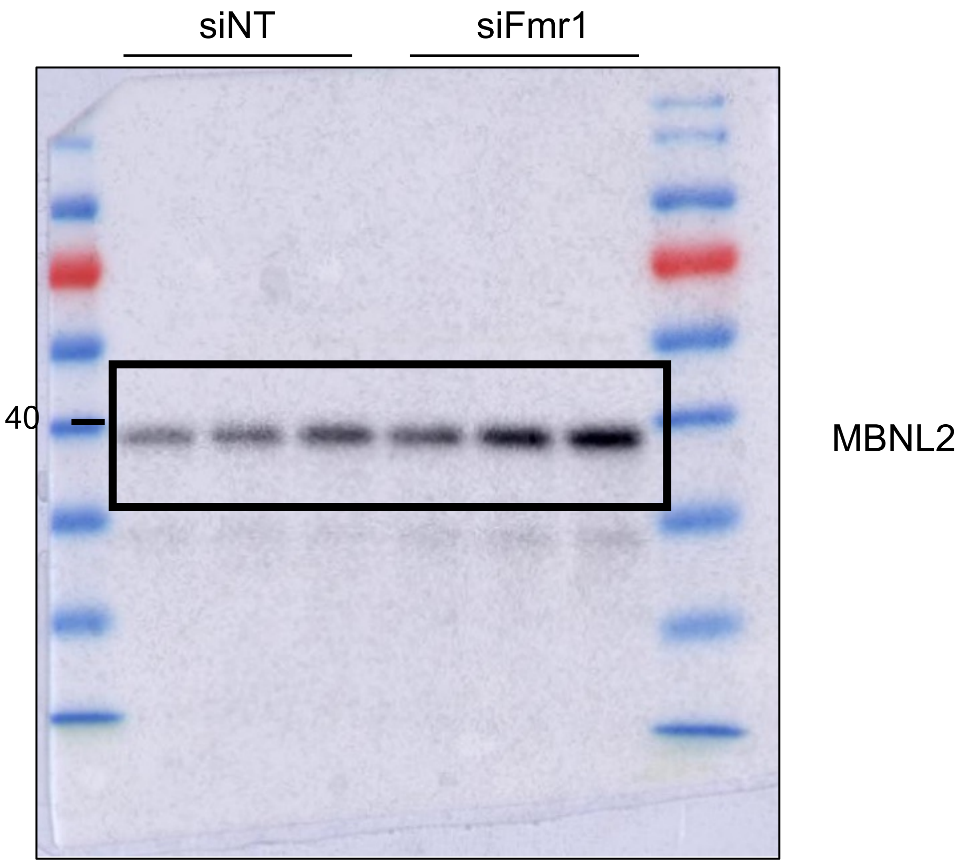

Figure 7B

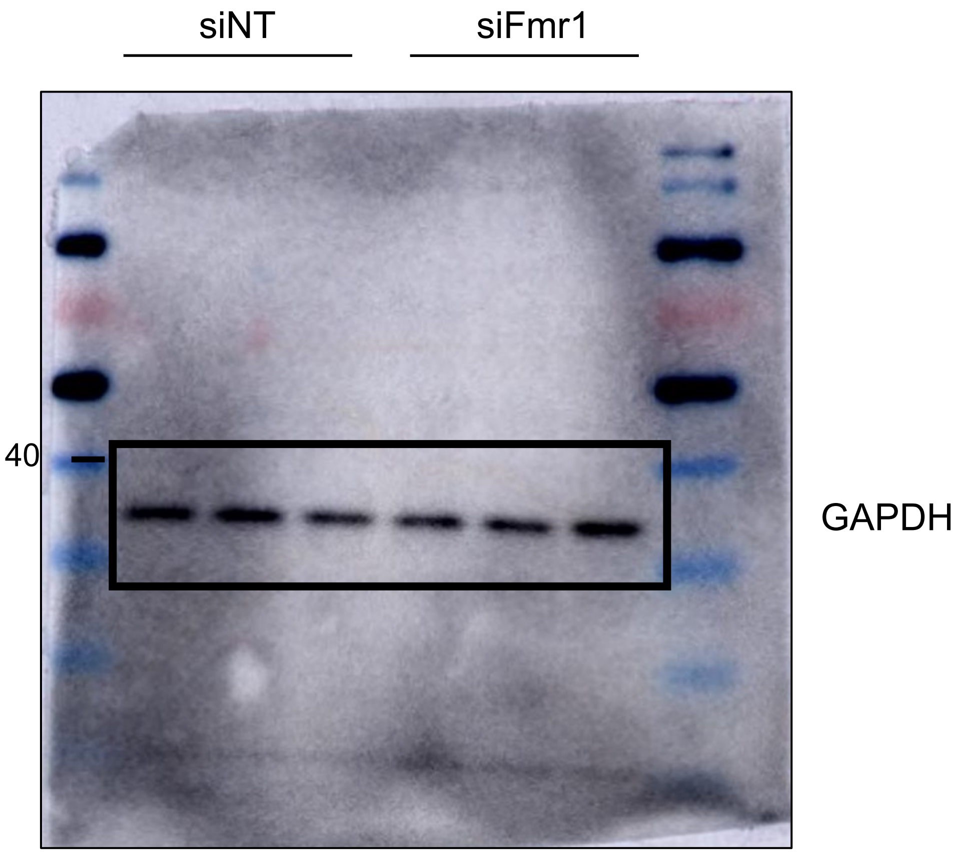

Figure 7B Relevant blots for MBNL2 quantification

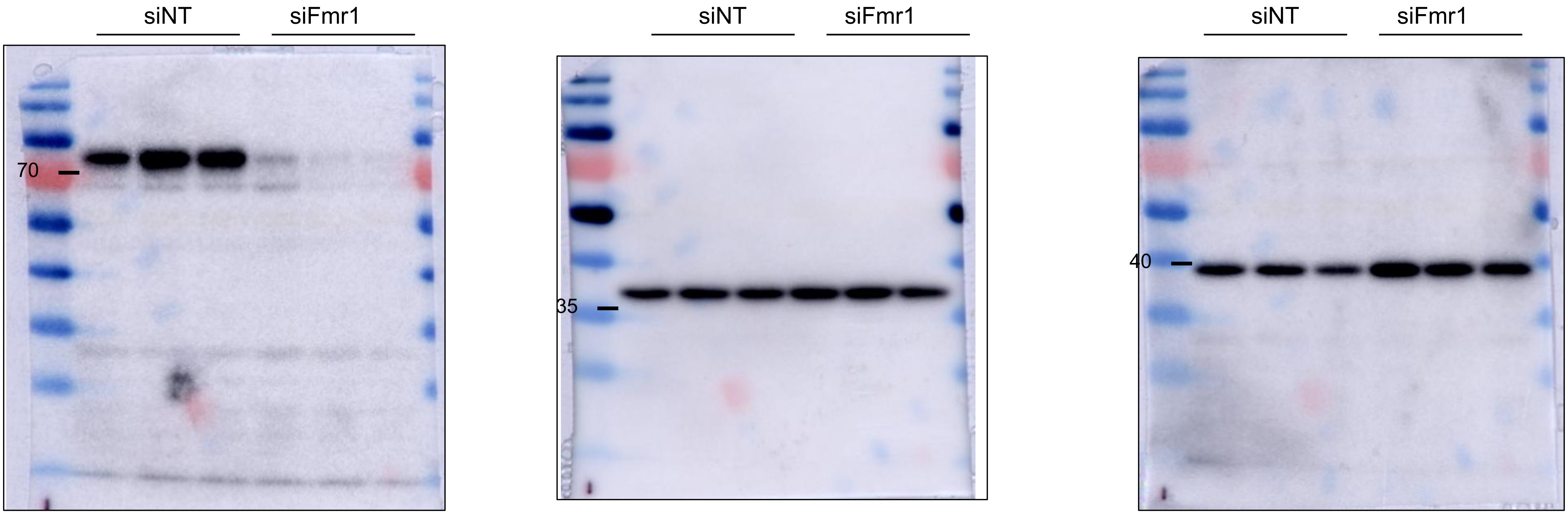

FMRP

GAPDH

MBNL2

Figure 7B Relevant blots for MBNL2 quantification

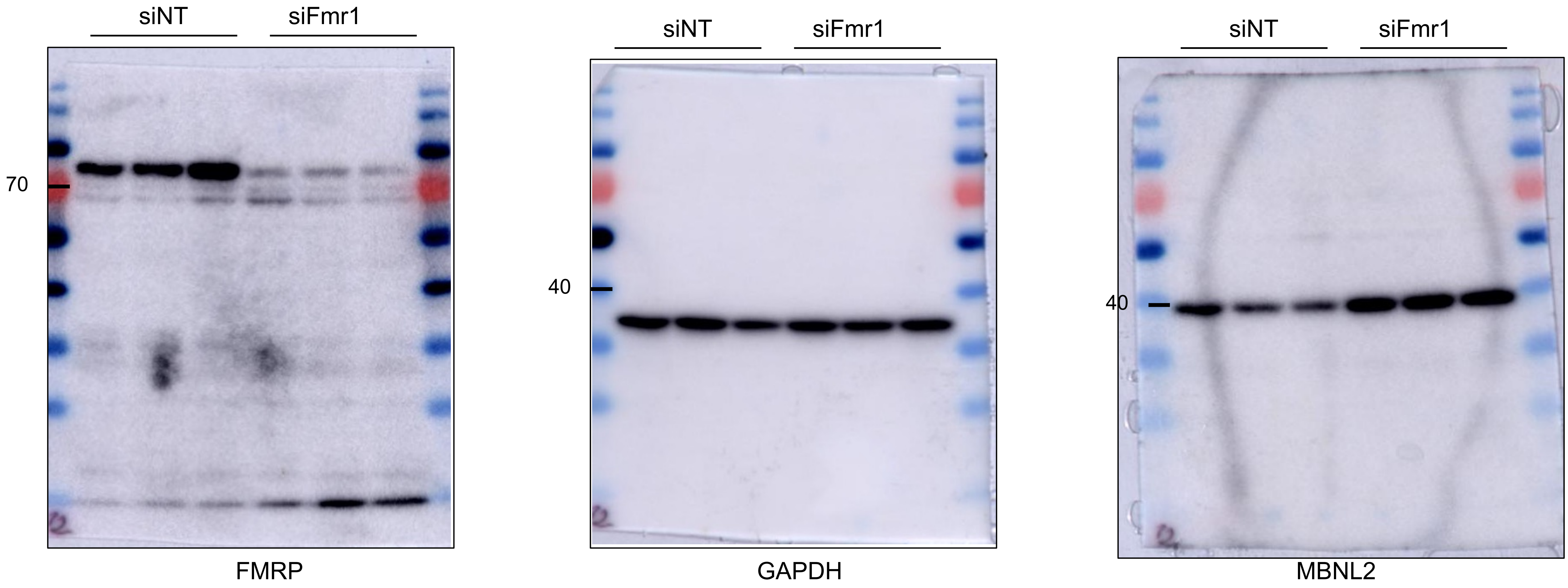

Figure 7B Relevant blots for MBNL2 quantification

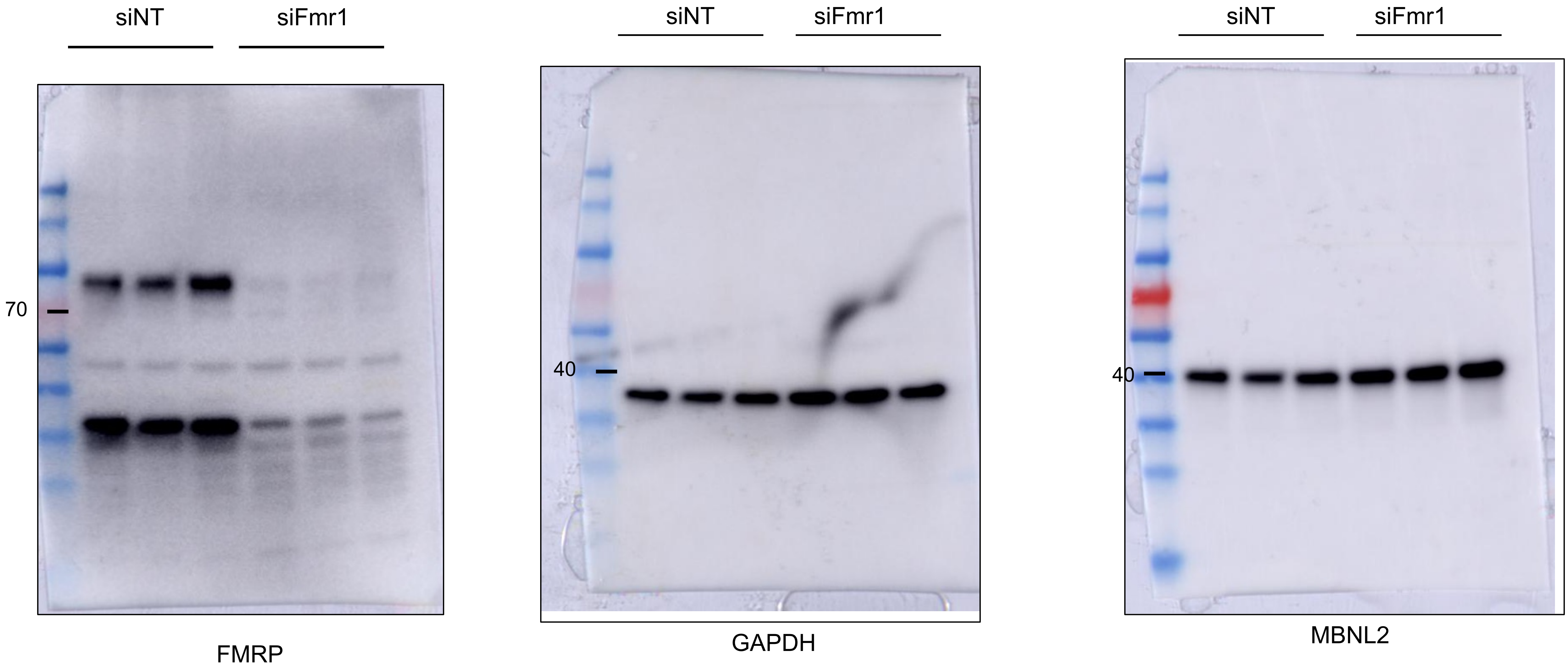

Figure 7B Relevant blots for MBNL2 quantification

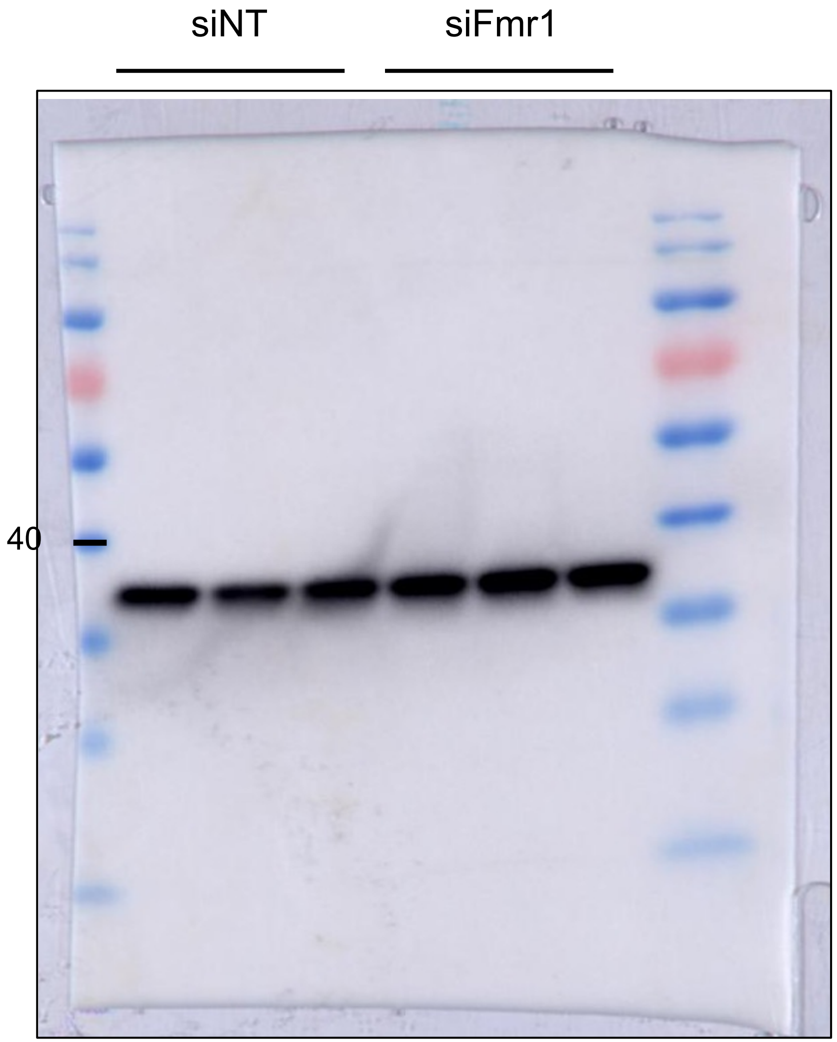

GAPDH

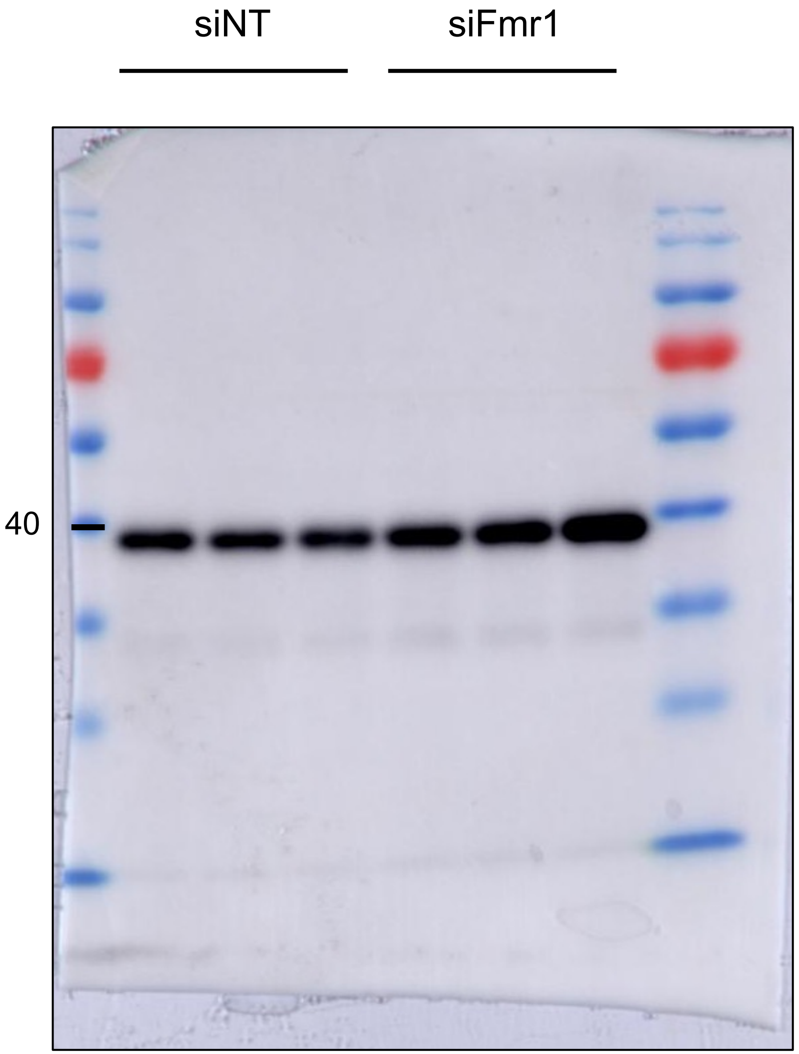

MBNL2

Figure 7C

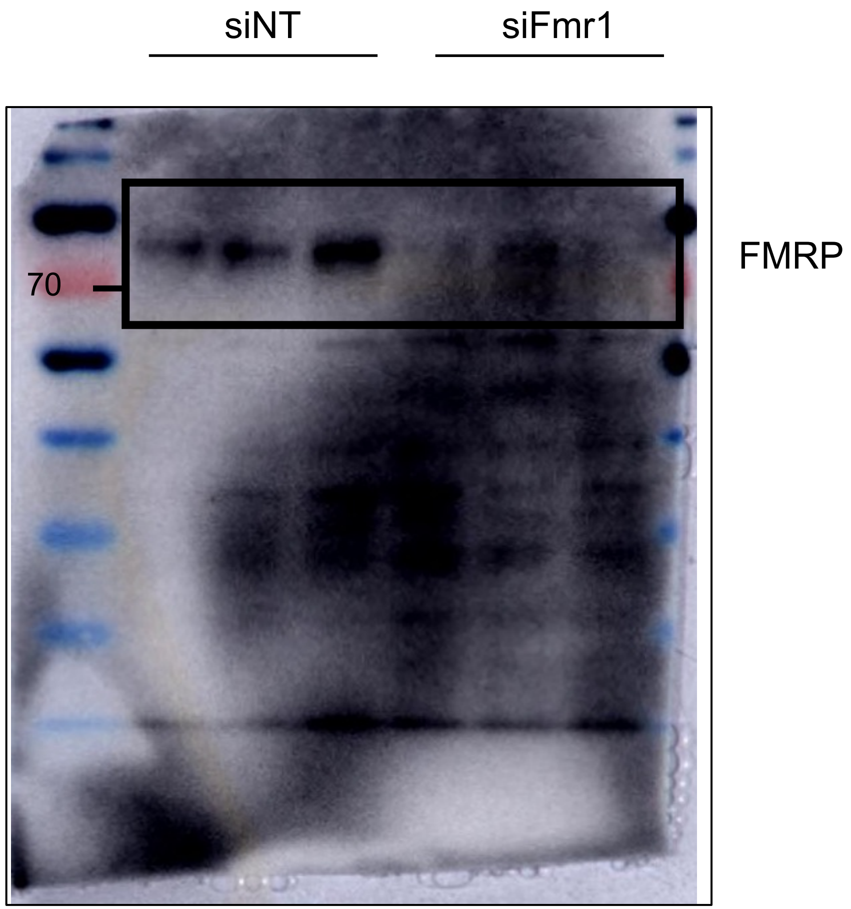

Figure 7C

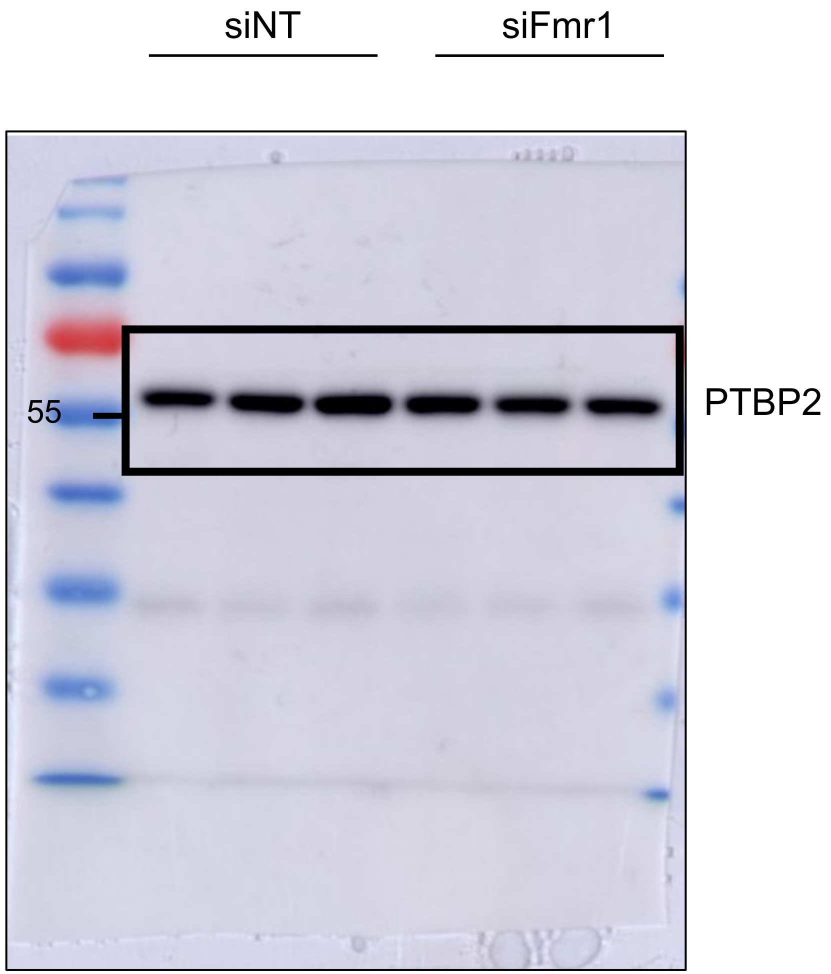

Figure 7C

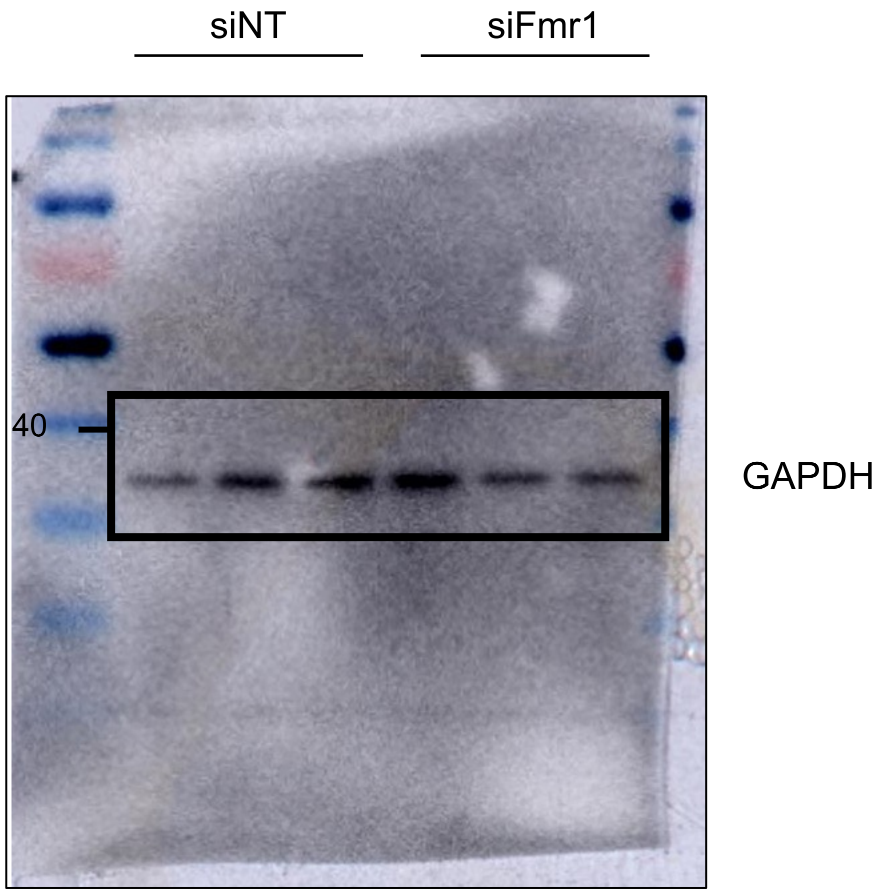

Figure S6A

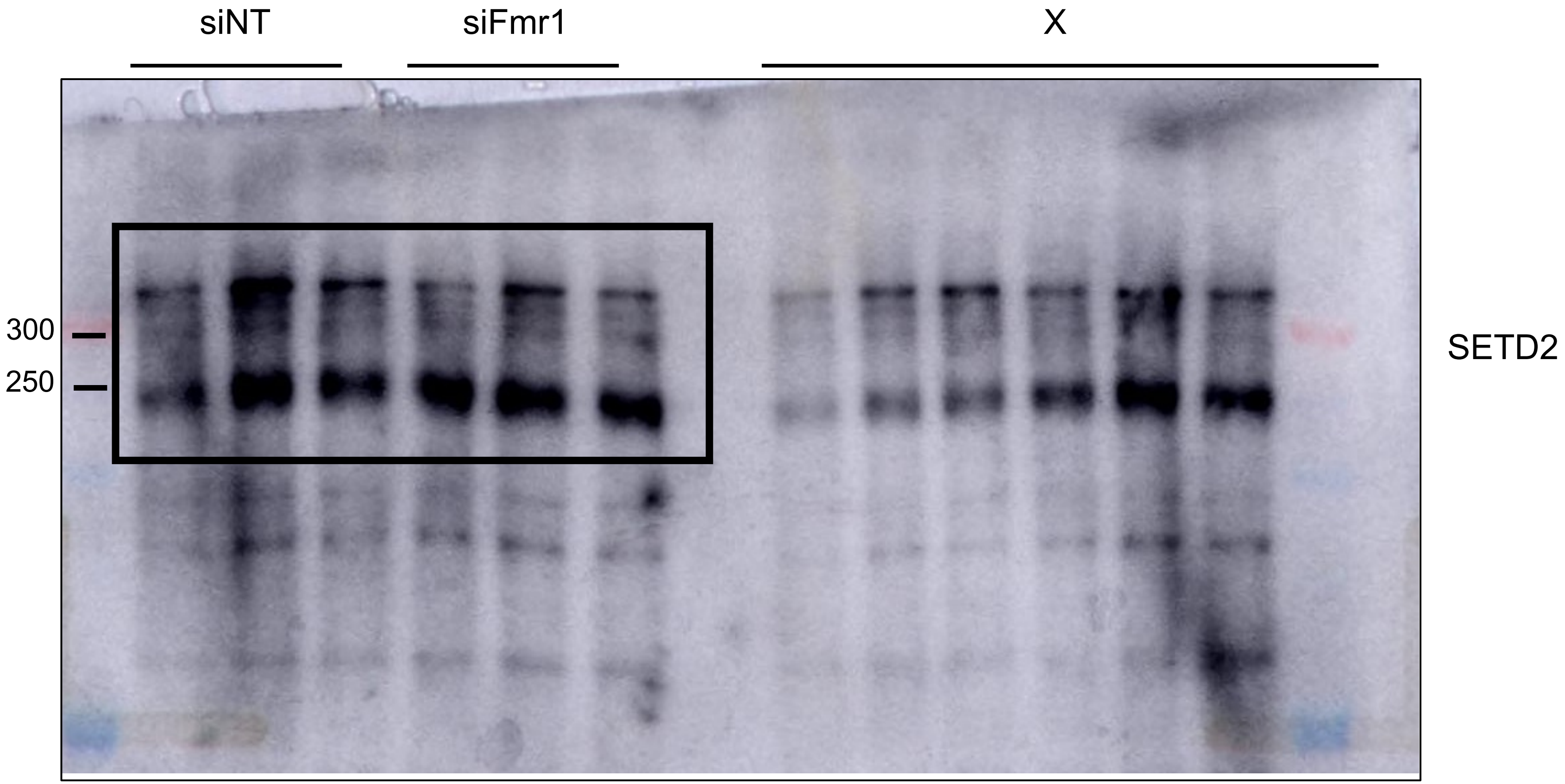

Figure S6A

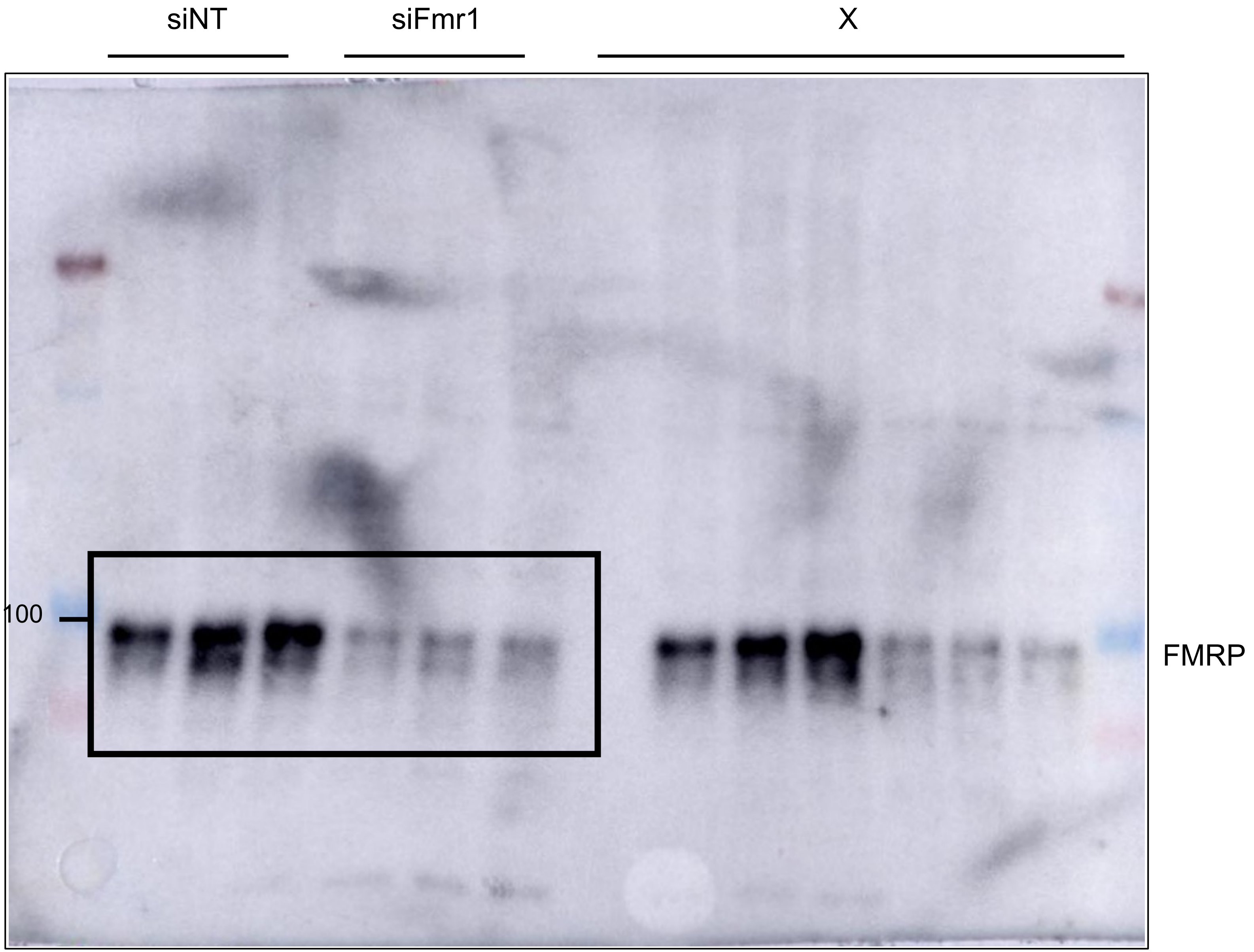

Figure S6A

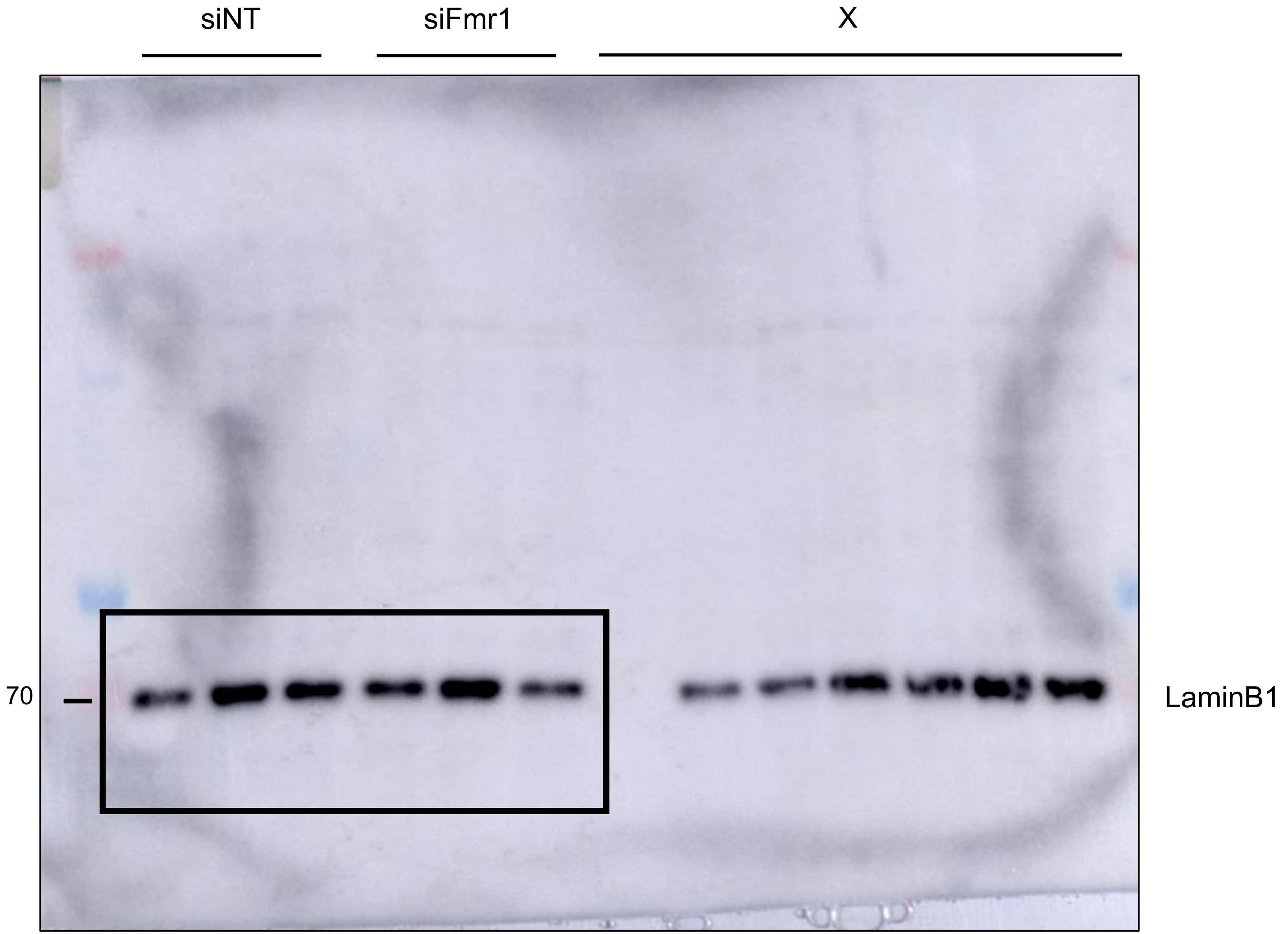

Figure S6A

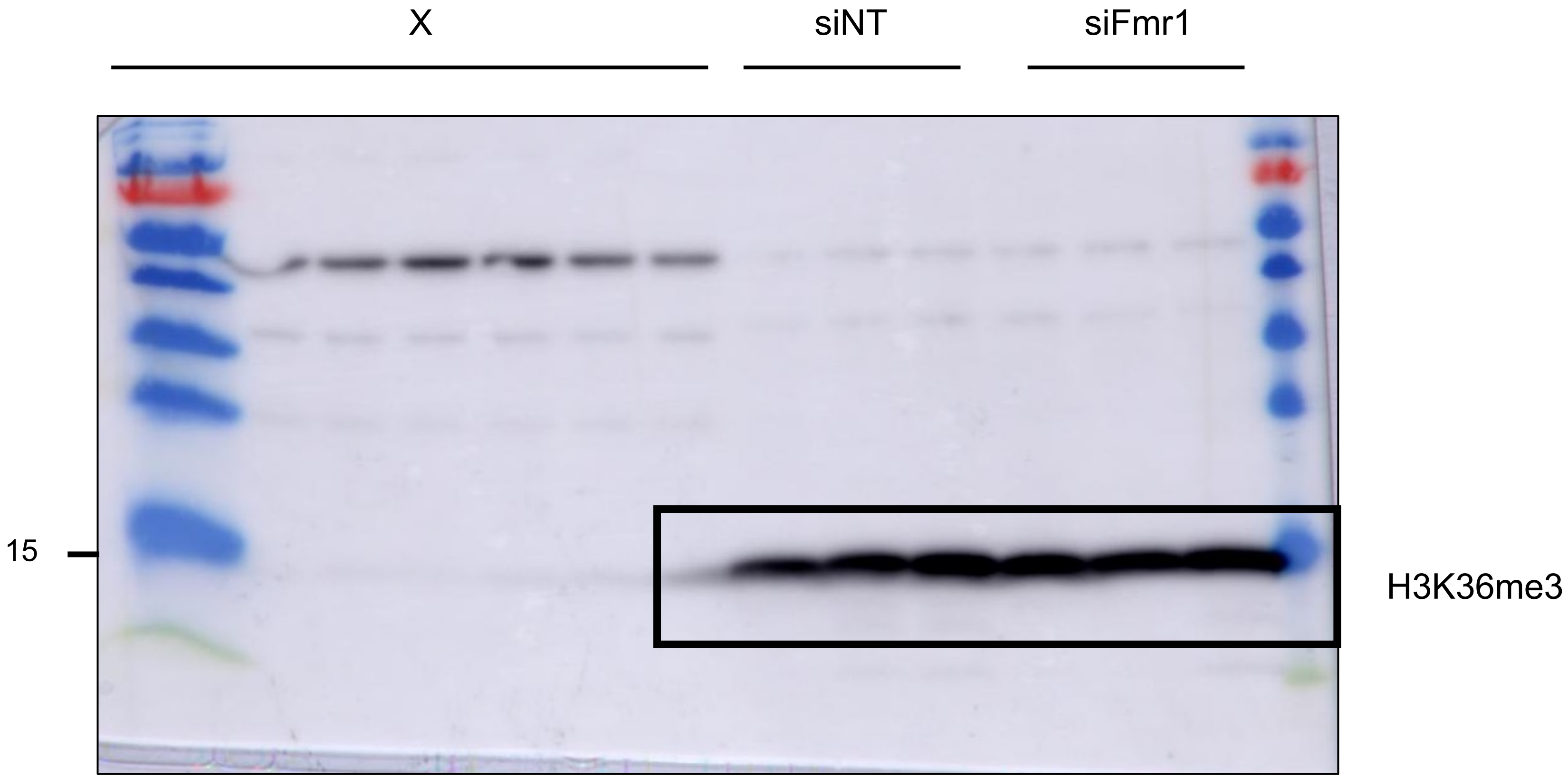

Figure S6A

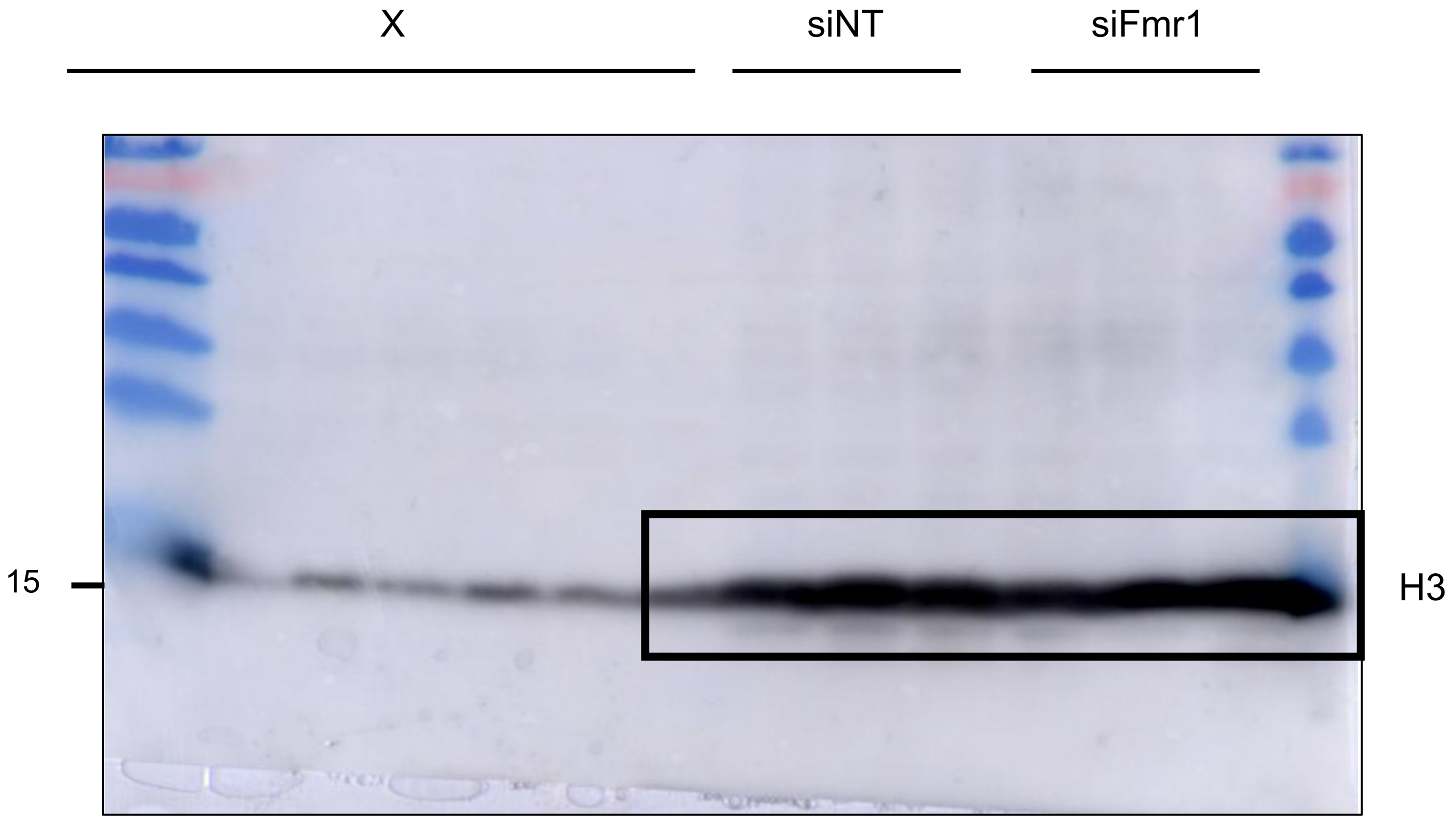

Figure S6B

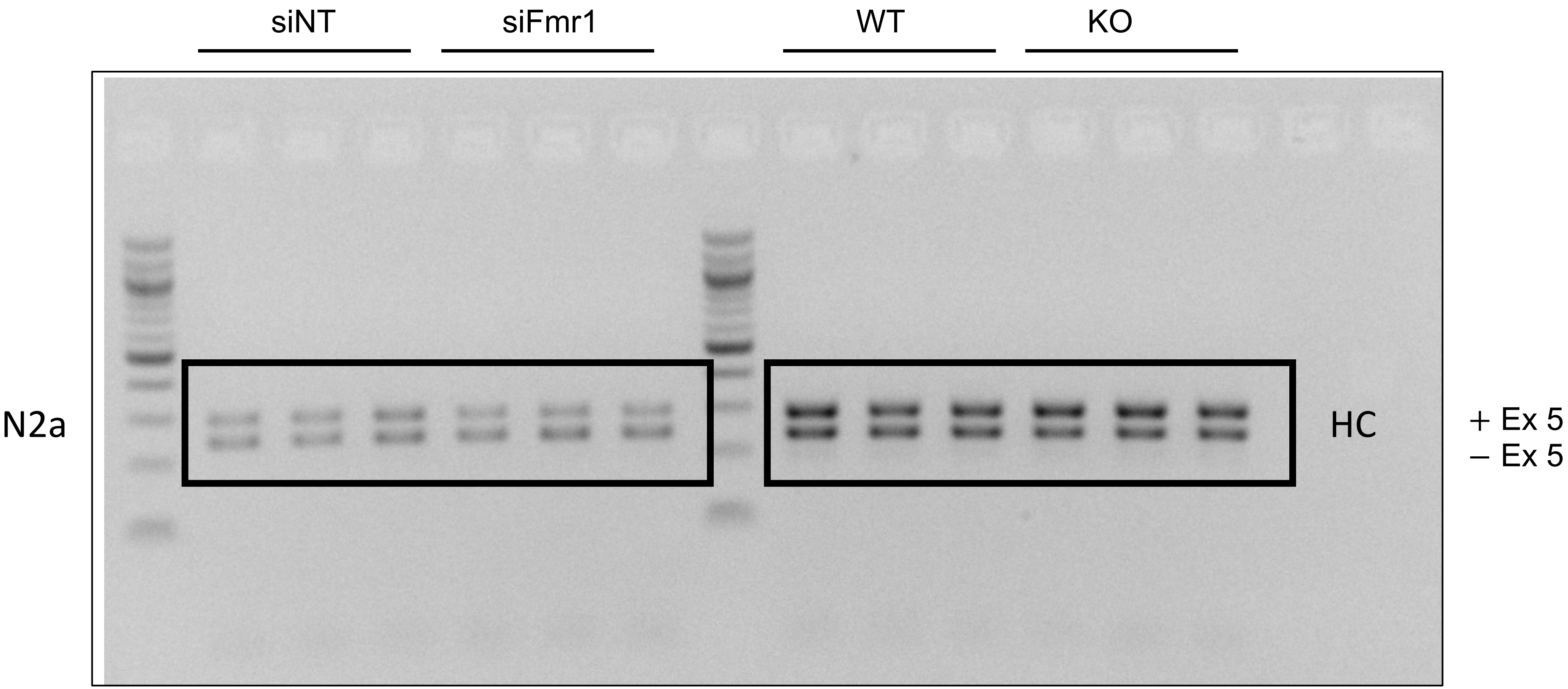

Figure S6B

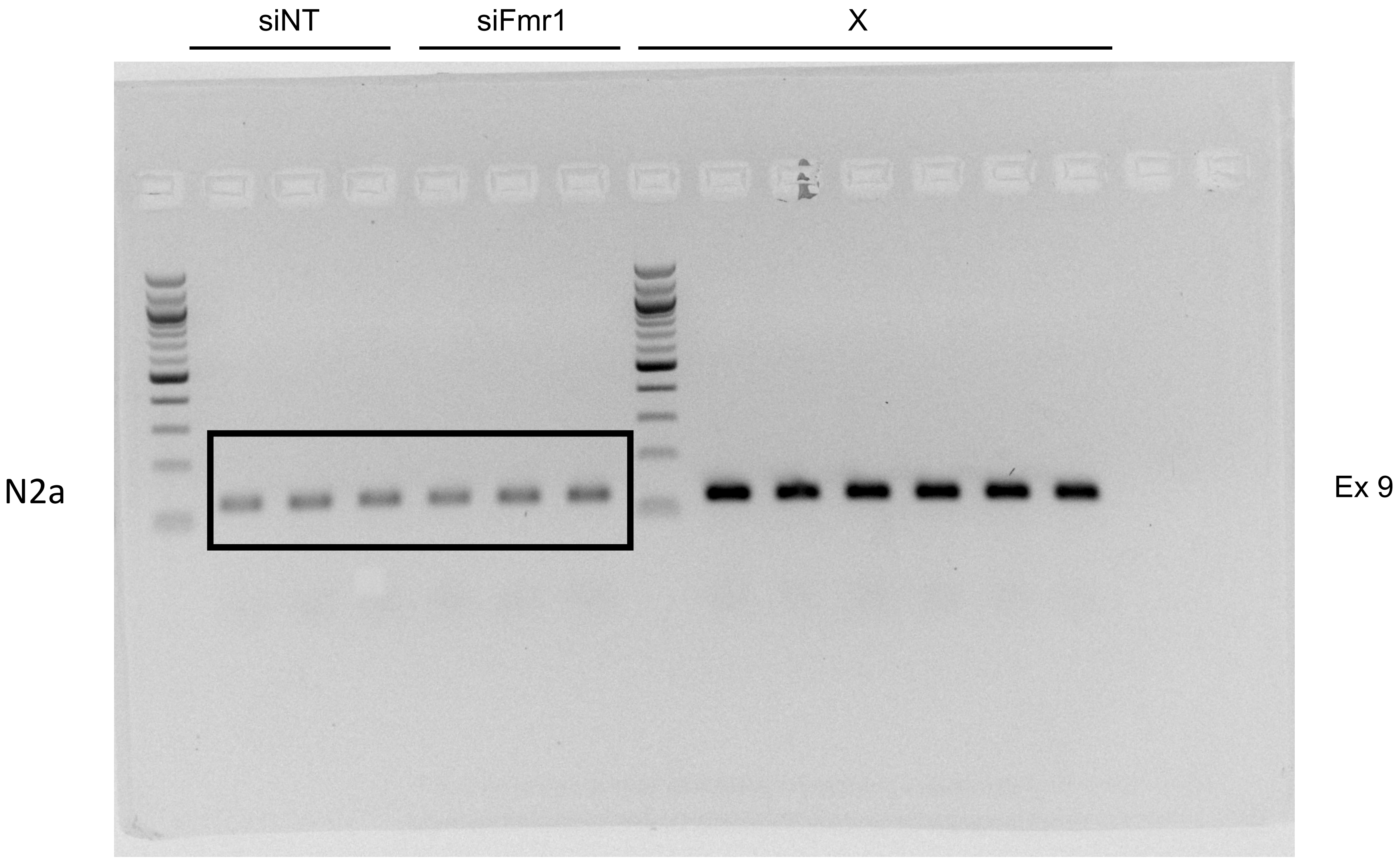

Figure S6B

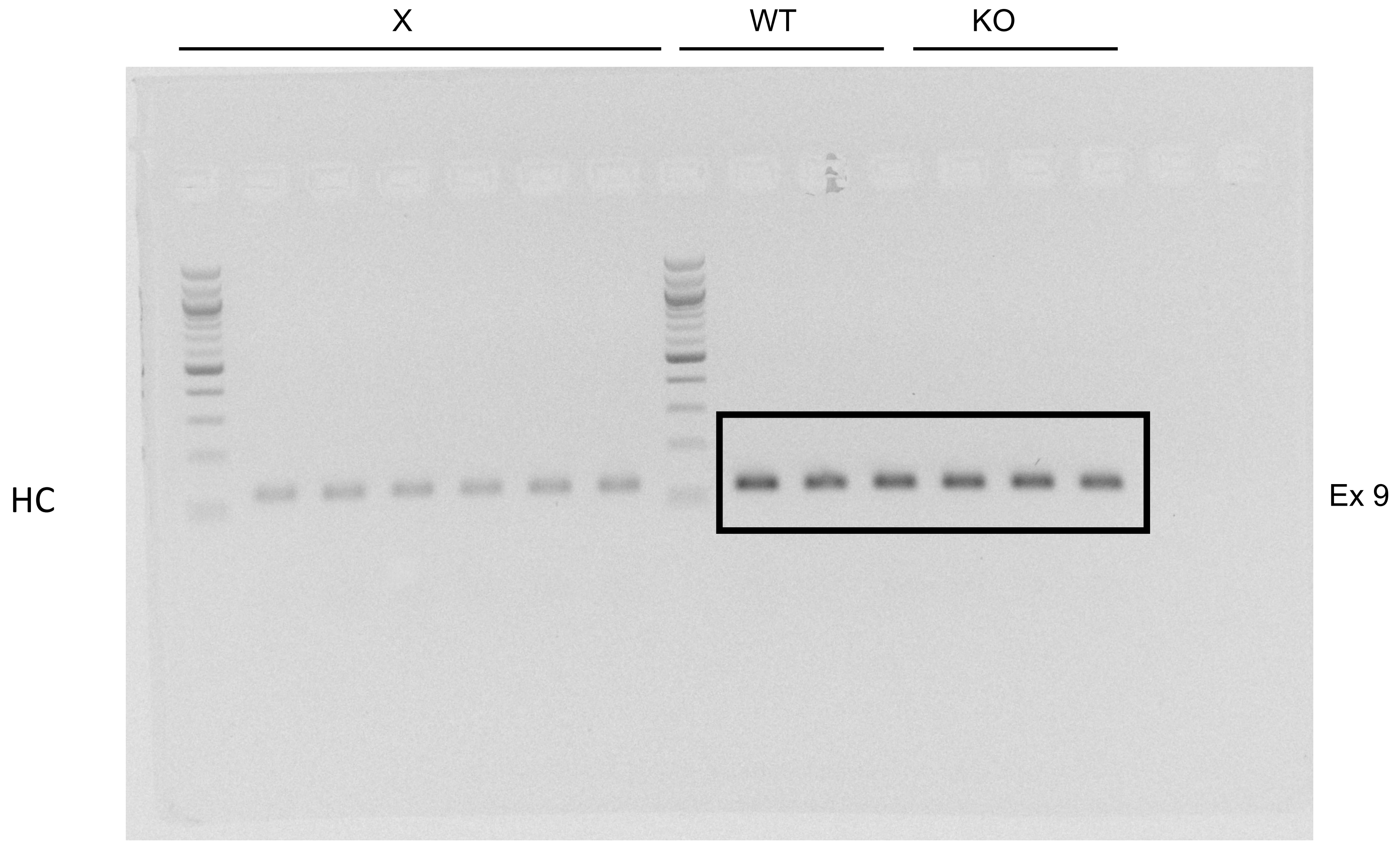

Figure S7A

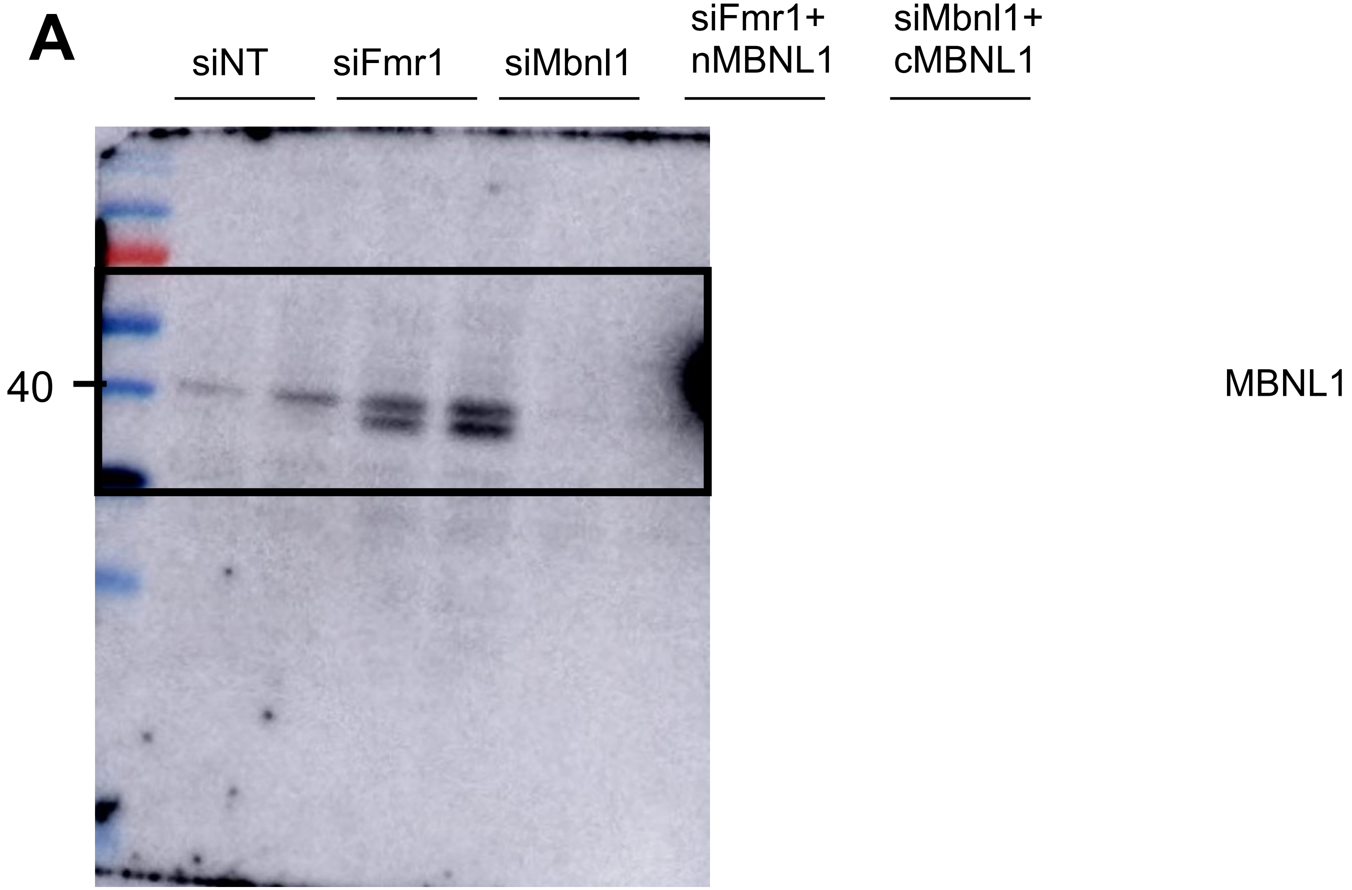
A
siNT
siFmr1
siMbnl1
siFmr1+
nMBNL1
siMbnl1+
cMBNL1
40
MBNL1

Figure S7A

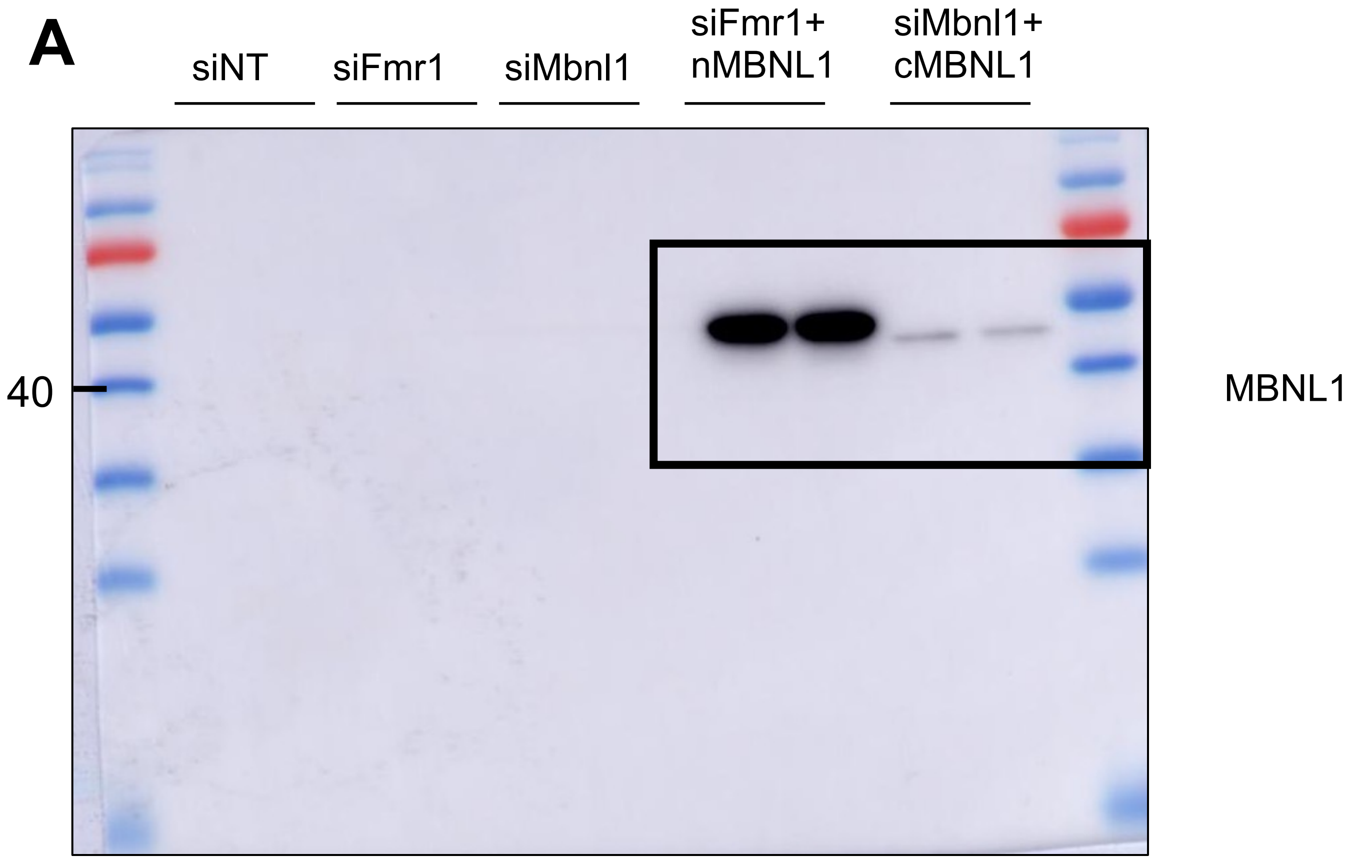
A
siNT
siFmr1
siMbnl1
siFmr1+
nMBNL1
siMbnl1+
cMBNL1
40
MBNL1

Figure S7A

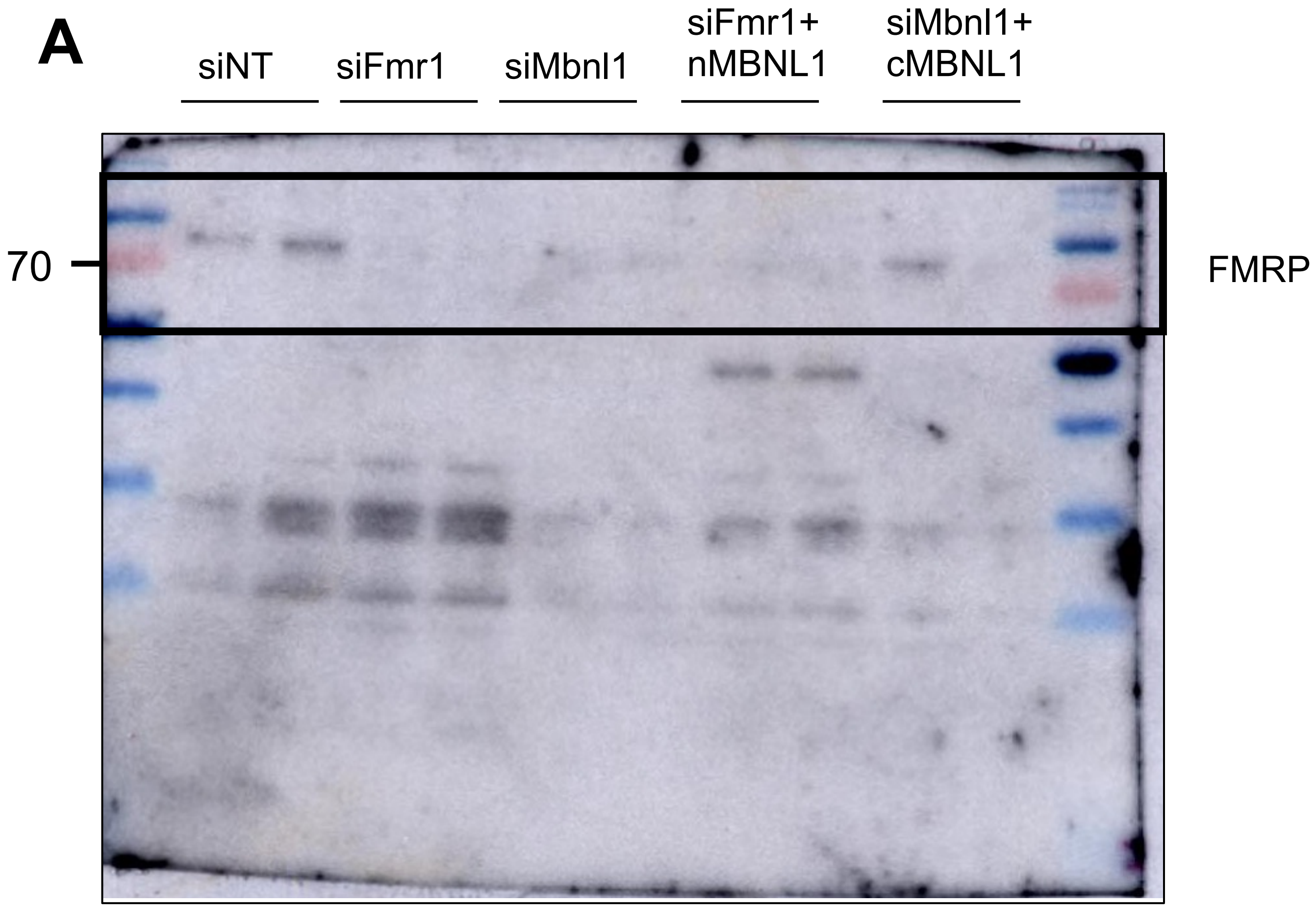

Figure S7A

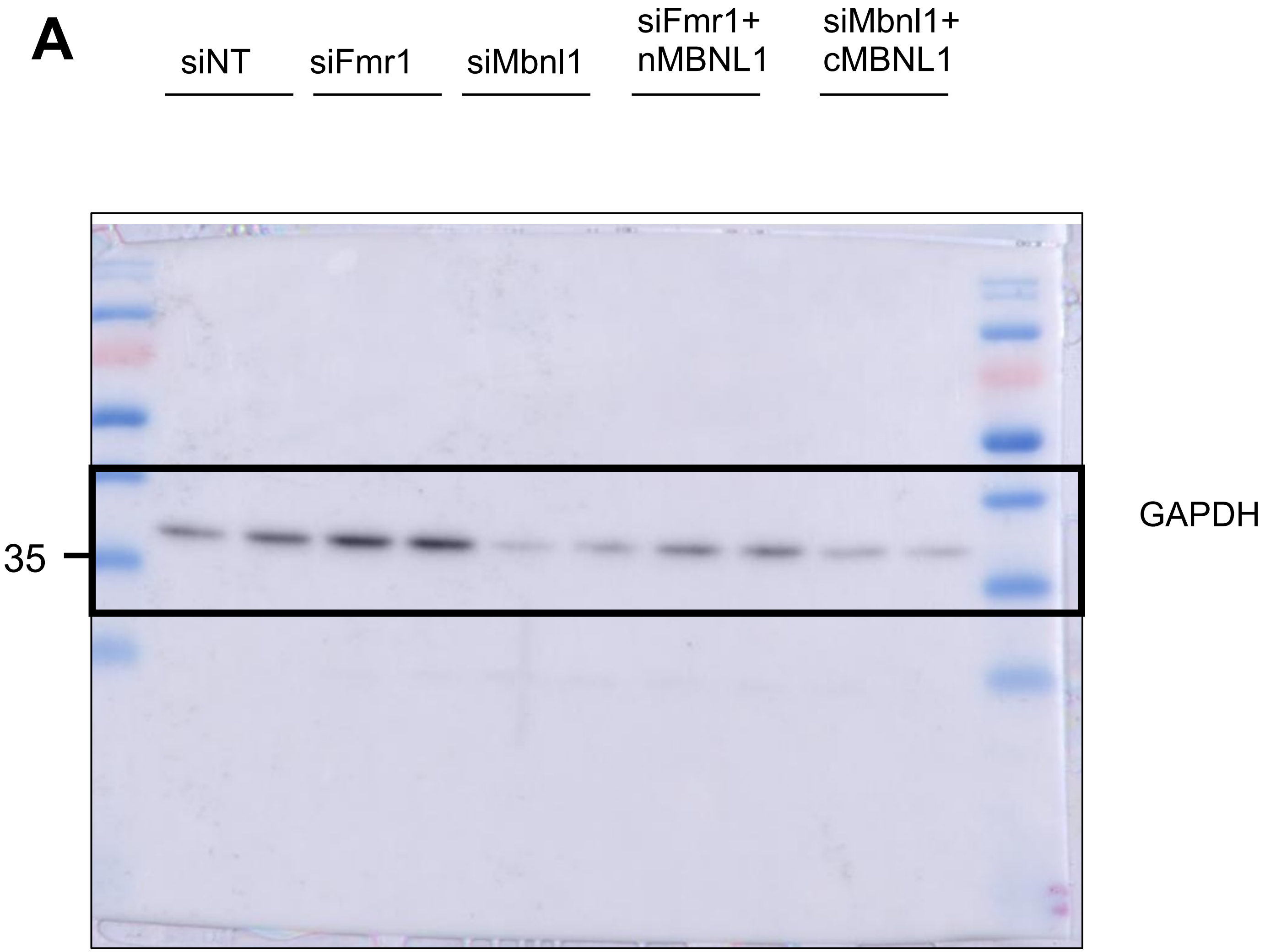
A
siNT
siFmr1
siMbnl1
siFmr1+
nMBNL1
siMbnl1+
cMBNL1
35
GAPDH

Figure S9B

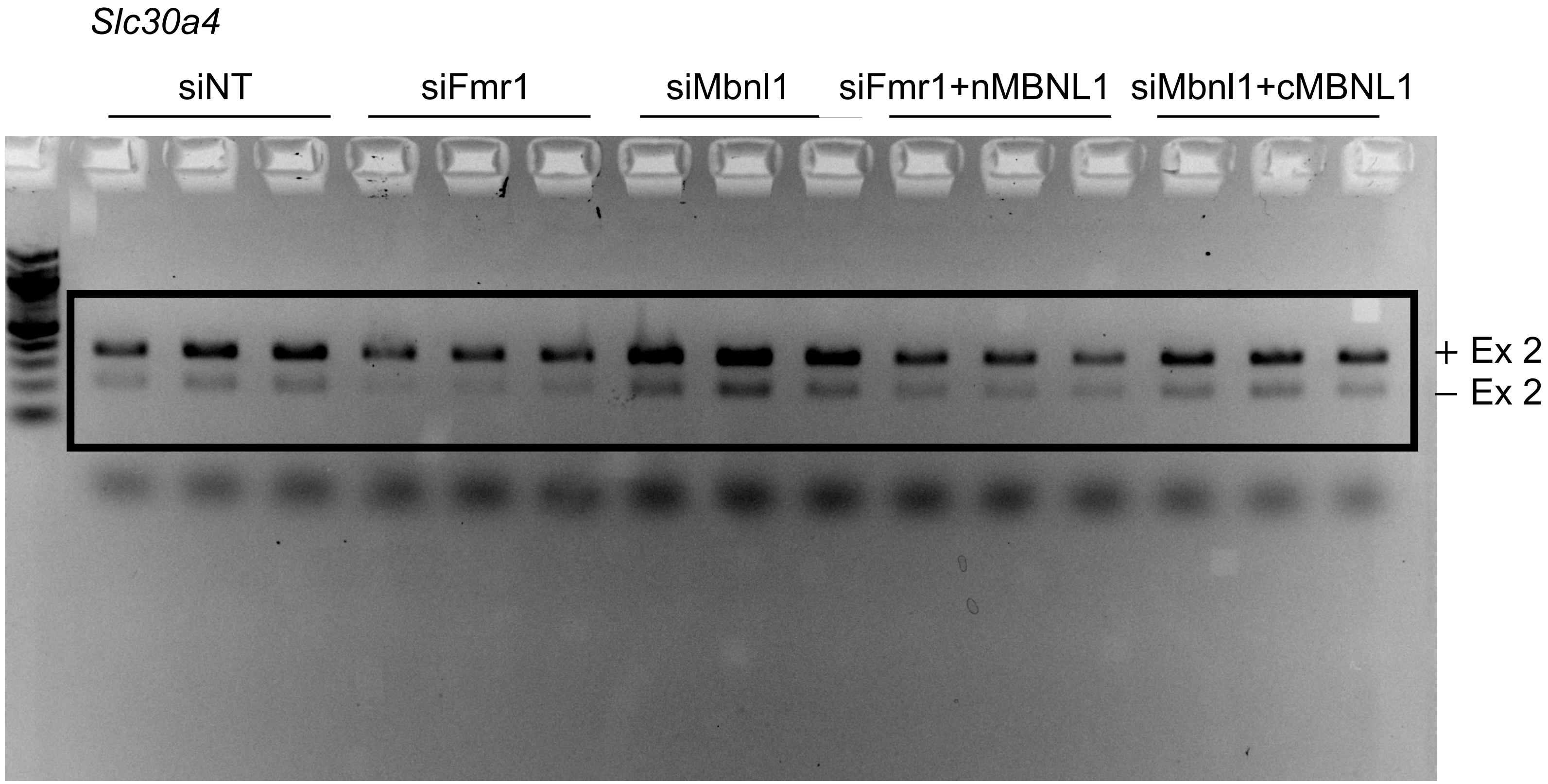

Slc30a4
siNT
siFmr1
siMbnl1
siFmr1+nMBNL1
siMbnl1+cMBNL1
+ Ex 2
– Ex 2

Figure S9B

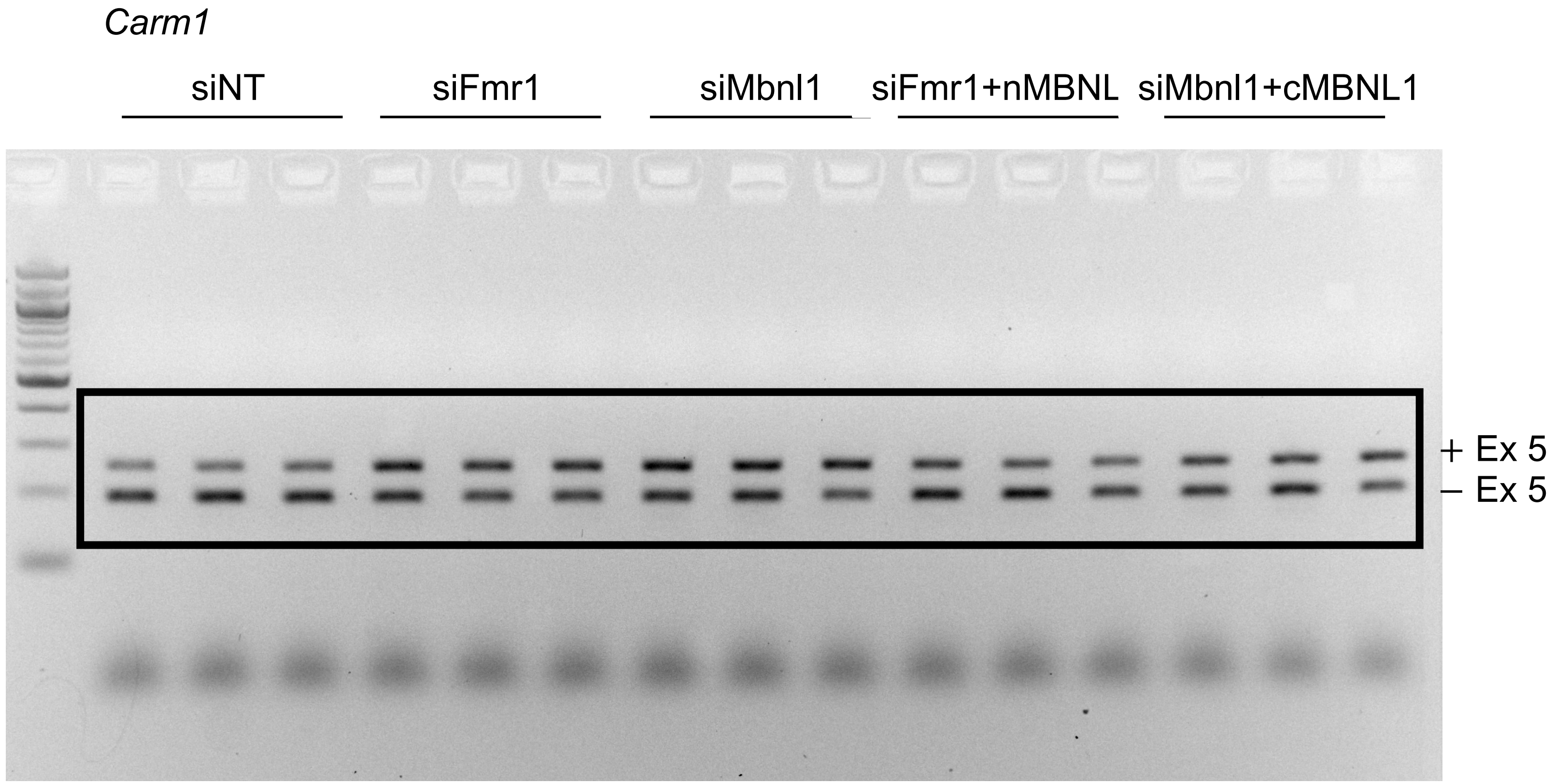

Carm1
siNT
siFmr1
siMbnl1
siFmr1+nMBNL
siMbnl1+cMBNL1
+ Ex 5
– Ex 5
