# Supplementary material for: FMRP deficiency leads to multifactorial dysregulation of splicing and mislocalization of MBNL1 to the cytoplasm
Source: PLoS Biol. 2023 Dec 4;21(12):e3002417. doi: 10.1371/journal.pbio.3002417 (PMC10721184; doi:10.1371/journal.pbio.3002417)
Supplement: Attachment — Submitted filename: ResponseToReviewers.docx [file pbio.3002417.s014.docx]

**Reviewer #1:**

In this manuscript, Jung and colleagues interrogated RNA dysregulation in FMRP-deficient mice and cultured cells, focusing on differences in alternative splicing regulation. They found that in FMRP-deficient cells, the inclusion rates of exons within transcripts encoding the splicing factors MBNL1 and PTBP1 was altered. Using this data, they postulate that this alters the activities of MBNL1 and PTBP1, leading to further downstream changes in the splicing of other transcripts. Although the data presented is interesting and suggestive, it is not enough to demonstrate that any of the changes in splicing observed are directly due to perturbations of MBNL1 or PTBP1. Further experiments are needed to justify this claim.

We are enormously appreciative of the reviewer for suggesting specific experiments/analyses to strengthen our manuscript. We have performed many new experiments as suggested by the reviewer and have answered all issues that were raised. Please see below for an item-by-item response.

MAJOR COMMENTS

1. My main concern comes from the authors' repeated ascribing of splicing regulatory effects by specific RBPs solely because (i) the inclusion of an exon changed in response to perturbation of the RBP and (ii) the exon is flanked by motifs that match the RBP's consensus binding site. As examples, this logic is laid out in figure 3A and in the text describing figure 4A. Showing that an RBP regulates the splicing of an exon requires both that the exon be differentially included upon RBP perturbation and that the effect requires binding of the RBP to the regulated transcript. Otherwise, the effect could be due to indirect effects that do not at all involve the RBP of interest.

The reviewer makes an excellent point and we have endeavored to address altered splicing events from both the RBP standpoint and from the transcript standpoint. Please see the following detailed explanations as well as new data that support our statements.

Using motif presence alone as evidence for regulation by an RBP is problematic for a few reasons. First, for any given RBP (and MBNL1 specifically, e.g. PMID 27720642), the vast majority of their consensus motifs are not actually bound by the protein in cells. This can be seen by comparing CLIP binding sites across the transcriptome to motif occurrences. The former is a small subset (often only 10-20%) of the latter.

The reviewer makes an important point and we are in complete agreement. Please see below for our experiments that address this issue.

Second, most RBP motifs are quite small. As an example, the consensus motif for MBNL1 is UGCU. As a 4mer, this motif would be expected to occur by random chance once every 256 nucleotides (using a simplistic model). In the examples presented in figure 3A, it looks like the abundances of motifs near the regulated exons may not be too different from the null expectation.

We agree, but as noted, we have mutated a putative MBNL1 binding site and indeed that alters splicing of Mapt pre-mRNA (please below).

There are a few ways the authors could bolster their claims. First, they could use published CLIP datasets for MBNL1 (e.g. PMID 27733504) and/or PTBP1 (e.g. PMID 27926877) to ask if sequences surrounding their favorite exons are actually bound by these RBPs. Expanding this, they could ask transcriptome-wide if exons that are differentially regulated upon FMRP loss are more likely to have MBNL1/PTBP1/etc CLIP peaks near them than FMRP-insensitive exons.

We have used the published CLIP datasets for MBNL1 (PMID 27733504) and PTBP1 (PMID 27926877) suggested by the reviewer. For Mapt pre-mRNA, there is no MBNL1 CLIP site of exon 4 (the exon under splicing control of FMRP). However, these authors performed a RIP-seq; they find a binding site in exon 4, but it does not contain an MBNL1 consensus site (UGCU). This might suggest that binding of MBNL1 is not always reflected by CLIP’ing.

In our RNA-seq dataset, we also found that FMRP regulates Mapt exons 2 (ΔPSI=0.09, p=0.02) and 3 (ΔPSI= -0.118, p=0.009), which both contain MBNL1 CLIP and RIP sites. When we were conducting our initial experiments, we focused on exon 4 because it appeared to have a greater ΔPSI (ΔPSI=0.134, p<0.003) upon FMRP depletion compared to exons 2 and 3.

In the PTBP1 reference (PMID 27926877), the authors listed genes with specific exons whose splicing was regulated by PTBP1. However, the CLIP sites lacked strong specificity. That is, the CLIP sites were only trinucleotides such UCU, UUC, CUU, etc, (their Fig 4B and C). The frequency of these sites makes comparison with our data very difficult because of the redundancy. Therefore, we have used another reference (Wang et al 2008, Nature 456:470) for PTBP1 binding sites. Using this reference, we find PTBP1 sites (hexanucleotides) upstream Mapt exon 4, and upstream and downstream of App exon 8, and upstream of Ski exon 2, downstream of Tnik exon 21. These data are presented in new Supplemental Figure S4 and S5).

As per assessing whether “exons that are differentially regulated upon FMRP loss are more likely to have MBNL1/PTBP1/etc CLIP” we have been unable to perform this analysis.

We have altered the text (page 12-13) to reflect the information noted above.

Similarly, if the authors wish to focus on RBP motifs rather than CLIP evidence, they could show that these motifs are actually enriched near regulated exons that simply present near them. As an example of this, when describing Data S9, the authors state that "50% of skipped or included exons in N2A cells contain binding sites for MBNL1 using RBPmap." This is fine, but how does that compare to a set of control alternative exons whose inclusion was unchanged? As stated above, given the high likelihood of these motifs to appear by chance, this comparison is a key control.

We have compared MBNL1 binding sites in FMRP target versus non-target RNAs. The FMRP targets were 50% whereas the non-targets were 36%. We have mentioned this in the text (page 13)

Finally, an alternative way to more directly show that these RBPs are regulating specific exons is to mutate the motifs they identified (e.g. those in Figure 3A) and ask if that alters the inclusion of the exon of interest, ideally in the same direction as perturbation of the cognate RBP. This could potentially be done using the splicing reporter described in Figure 2G.

In response to the reviewer’s excellent suggestion, we mutated a putative MBNL1 binding site near Mapt exon 4, the exon that is included more in FMRP knockout compared to control. New Figure 2H shows that after mutation of this site (UGCUGC), the exon splicing is statistically identical to control.

2. In figure 6, the authors analyze the inclusion of alternative exons with Mbnl1 RNA in response to FMRP perturbation from a range of different samples, arguing that because specific exons (e.g. exon 5 and exon 7) are repeatedly identified as FMRP-sensitive across a range of samples, the effect is reproducible. However, the direction of the regulation for a given exon is different between many of the samples (look at Inclusion difference [a.k.a. delta PSI] column). To me, this argues that the regulation of Mbnl1 splicing is not directly due to FMRP and that these highly regulated exons themselves are being indirectly regulated by something else.

In our dataset, we found that FMRP regulates the splicing of various other RNA-binding protein (RBP) mRNAs in a tissue specific manner. These differences could be partly responsible for changes in the direction of MBNL1 splicing. The table below illustrates this point. We now mention that specific exons could be differentially alternatively spliced in various tissues due to different amounts/activities of splicing factors (page 16).

This table shows RNAs that are differentially spliced in the different tissues in FMRP KO mice. These RNAs encode proteins involved in RNA splicing.

3. All of the RT-PCR experiments to quantify alternative splicing are nice to see, but it could be helpful to show metrics for those exons from the RNAseq data (e.g. delta PSI, FDR, etc.) Seeing that the RNAseq/rMATS analysis and RT-PCR generally agree would lend confidence to the results.

We now provide in Figure 2C the RNA seq metrics as requested by the reviewer, which are similar to the RT-qPCR data.

4. In multiple places, when the authors are describing cutoffs for identifying significant exons to focus their analysis on, the metrics presented are confusing (e.g. log2FC p < 0.05, PSI > 0.05). The software the authors used, rMATS, does not report log2FC. log2FC is very rarely used in splicing analysis. A much better metric would be "delta PSI" which compares the average PSI value for a given exon between the two tested conditions. In rMATS output, this is the IncLevelDifference column. rMATS then computes a multiple-hypothesis corrected FDR value for if the two sets of PSI values for an exon are different across conditions. This may be what the authors mean rather than log2FC p.

We inadvertently mislabeled our data as log2FC when in fact it was p<0.05. We have corrected this labeling in the figures and in the text. We also corrected PSI to delta PSI as suggested by the reviewer.

Similarly, it does not make sense to use "PSI" as a metric here since that is the value for an exon in one condition, when what you want here are exons that are differentially included between two conditions. Thus the term "delta PSI", which is the difference in PSI values between conditions, is normally used.

We corrected PSI to delta PSI as suggested by the reviewer.

Finally, in order to study both exons that are more included and more skipped, it is common to set the delta PSI cutoff at abs(0.05), i.e. > 0.05 and < -0.05.

We agree and have correct to |delta PSI|>0.05.

All of this may be what the authors actually did, but the way it is communicated is confusing and likely incorrect.

This is indeed what we did – we apologize the confusion.

5. Similar to point 4, the utility of figure 1F is unclear. It would be more useful to plot delta PSI between the wildtype and KO conditions. I know that PSI, and not delta PSI is currently being plotted because PSI ranges between 0 and 1 and delta PSI ranges between -1 and 1. Plotting delta PSI would allow the visualization of broad, coordinated changes in alternative isoforms, for example a trend to global inclusion or exclusion of alternative exons.

As per the reviewer’s suggestion, we have replotted Fig 1F to now reflect delta PSI.

6. Is there an overall correlation in gene expression and splicing changes between the N2A Fmr1 knockdown and knockout experiments? This would lend further confidence to this data.

We previously performed RNA-seq using multiple Fmr1 CRISPR KO cell lines that were low depth and have not included this dataset in the manuscript as it needs further detailed experimentation. It is important to note that the CRISPR line was used only for the reporter experiment whose readout was flow cytometry and RT-qPCR for a specific sequence. Moreover, our datasets from new Fmr1 knockdown experiments (Figures 8 and 9) show statistically significant and reproducible Mbnl1 exon 5 splicing alterations.

MINOR COMMENTS

1. In multiple places in the text, the authors use the term "Mbnl1 RNA self-splicing" or variants thereof to describe the known regulatory effect that MBNL1 protein has on the splicing of the RNA that encodes it. I understand what the authors are trying to say, but the term "RNA self-splicing" already has a very specific meaning (e.g. group II intron self-splicing) that is quite different from what is being discussed here. Rephrasing this could improve the clarity of the manuscript.

We appreciate the reviewer’s comment and now refer to this event as MBNL1 auto-splicing.

2. In the introduction, the sentence "Splicing factors MBNL1, PTBP1, and HNRNPF are responsible for altered splicing in Fmr1-deficient cells. FMRP regulates the translation of two of these factors, MBNL1 and HNRNPQ." This is confusing as HNRNPQ is not one of "these" factors mentioned in the previous sentence.

The reviewer is correct – we have altered the text accordingly (page 4).

3. When describing figure 3B, the authors state that "Because the magnitude of Mapt exon 4 inclusion was additive when both Mbnl1 and Fmr1 were depleted, we surmise that a second splicing factor under the control of FMRP is involved in this splicing event."

This may be true, but it could also be true that residual Mbnl1 RNA that survives the Mbnl1 knockdown is then further misregulated upon Fmr1 knockdown (perhaps translationally, as the authors show). This would give an additive effect without the need to invoke a second RBP.

The reviewer is correct and we have altered the text accordingly (page 11).

**Reviewer #2:**

The manuscript interrogates the factors that might mediate the changes in RNA Splicing that are observed upon deficient FMRP activity. The authors previously reported a correlation between the up-regulation of SETD2, altered H3K36me3 chromatin marks, and RNA splicing mis-regulation in Fmr1-deficient mouse brain. Now they interrogated splicing changes in more depth with Fmr1 KO N2A cells, and detected no alteration in SETD2 levels, concluding that to splicing dysregulation is more likely driven by factors other than a change in H3K36me3. The study focuses on the splicing factors hnRNPF, PTPB1 and MBNL1, which are candidate regulators of specific observed splicing changes. The study focuses mostly on MBNL1, because FMRP directly regulates the translation of Mbnl1 mRNA, which can explain a subset of downstream splicing changes including altered Mbnl1 self-splicing, and altered Mbnl1 self-splicing also occurs in human FXS post-mortem brain. This is a potentially valuable study showing how regulation of translation and RNA processing might be coupled via cross-regulation between RNA-binding proteins. However, the data supporting the conclusions is quite limited at present, and more thorough investigation with RNAseq would be needed.

We thank the reviewer for his/her comments and for indicating that the study is “potentially valuable.” We have performed many new experiments that address the stated concerns.

Major suggestions

The study finds a strong increase in the abundance of MBNL1 and hnRNPQ upon Fmr1 depletion, but MBNL1 shows a slightly decreased tendency to localise to the nucleus, possibly due to its splicing shift. For now, I have a hard time seeing much change in nuclear abundance of MBNL1, or estimating how Fmr1 should be expected to affect its splicing activity. It seems that the slight change in nuclear transport is compensated by increased abundance of MBNL1. Iit would be helpful if absolute abundance of MBNL1 in the nucleus could be quantified and presented.

The reviewer is correct in that loss of FMRP changes both splicing of Mbnl1 RNA and abundance of MBNL1 protein. However, in Figure 5C, we quantified the western blot of nuclear MBNL1 based on the amount of nuclear Lamin B. The histogram, labeled Upper MBNL1/LaminB1, shows that upon FMRP depletion, MBNL1 is indeed reduced. Thus, the amount of nuclear MBNL1 is reduced when FMRP is depleted. If the reviewer is referring to, for example, ng of protein/nucleus or cell, we are unable to provide this information.

Lack of clear effect on nuclear MBNL1 levels makes it difficult to understand how MBNL1 might mediate splicing changes caused by Fmr1 depletion. Moreover, evidence from a few chosen exons is also limited -, then co-depletion of Mbnl1 and Fmr1 only shows an additive effect for Mapt Exon 4. In addition to co-depletion, authors should also perform experiments where Fmr1 depletion is coupled with transfection of cDNA expressing one or the other MBNL1 isoform, to show that only transfection of the NLS-containing isoform can rescue Mapt Exon 4 splicing back to control levels.

Based on the quantifications in Fig 5C, nuclear MBNL1 is decreased by ~40% upon FMRP depletion. Based on the reviewer’s suggestion, we have now performed extensive experiments to assess the effect of nuclear MBNL1 on overall splicing. We transfected siNT, siFmr1, or siMbnl1 alone or siFmr1 plus a plasmid expressing full length MBNL1, which is nuclear as in Wang et al., 2018 (Cell Reports 22:2294). We also transfected siMbnl1 plus plasmid expressing MBNL1 that lacks exon 5, and thus is cytoplasmic as in Wang et al., 2018. The reason we transfected siMbnl1 was to reduce the effects of endogenous Mbnl1.

The data from these experiments are now presented in new Figures 8 and 9.

If the above is the case, the authors should perform RNAseq analysis of cells treated with various siRNA or co-transfection combinations to show more thorough evidence for the claim that loss of nuclear MBNL1 mediates a subset of splicing changes induced by Fmr1 depletion.

Please see above.

Minor suggestions

I find the statement 'FMRP-Regulated RNA Splicing' in the title potentially misleading, because such statements normally refer to direct regulation. FMRP doesn't regulate RNA  Splicing - instead it regulates translation. I'd find a title along these lines clearer: "FMRP Affects Splicing by Regulating Translation of Multiple Splicing Factors, Including Mbnl1"

Based on new data, we prefer the title:

FMRP-regulated alternative splicing is multifactorial and resembles splicing control by MBNL1.

The abstract states: When FMRP is lost, SETD2 levels are excessive, which alters the H3K36me3 landscape and secondarily, alternative pre- mRNA splicing. - however, the paper rejects this statement with the conclusion "a changed chromatin landscape and altered splicing in FMRP- deficient cells may not be linked", but one wouldn't realise this by reading the abstract. I'd find it more informative if SETD2 was mentioned at results, rather than introduction of the abstract, but stating sth along: We find that the previously reported changes in chromatin landscape may not be linked to altered splicing in FMRP-deficient cells, but instead specific RNA binding proteins…"

We did mention the SETD2 data in the results (page 16) and show the data in Supplemental Figure S6A.

The abstract also states: "Here we show that in Fmr1-deficient mice, RNA mis-splicing occurs in several brain regions and peripheral tissues." This makes an impression that we're looking at a new finding, but a similar observation was already made previously by the authors (<https://nam10.safelinks.protection.outlook.com/?url=https%3A%2F%2Fpubmed.ncbi.nlm.nih.gov%2F32234480%2F&data=05%7C01%7CJoel.Richter%40umassmed.edu%7Cf0568dfb527d40ff9a4608db692aca99%7Cee9155fe2da34378a6c44405faf57b2e%7C0%7C0%7C638219404818740819%7CUnknown%7CTWFpbGZsb3d8eyJWIjoiMC4wLjAwMDAiLCJQIjoiV2luMzIiLCJBTiI6Ik1haWwiLCJXVCI6Mn0%3D%7C3000%7C%7C%7C&sdata=3tM196R6PxW2Yy2NdYNRPOkdV7fv9hnjioTn84j7hdk%3D&reserved=0>). The abstract should make it clearer what was known and what is new in terms of discovered splicing changes.

FMRP regulation of splicing is indeed already mentioned in the abstract, but we now make it clearer that we had published previous data on mouse hippocampus.

**Reviewer #3:**

In this manuscript, Jung and colleagues study the role of FMRP, a protein that is absent in Fragile X Syndrome (FXS), in pre-mRNA splicing.

First, the authors analysed gene expression in an Fmr1 KO mouse model, focusing on brain tissues (hippocampus, cerebellum, cortex), as well as peripheral tissues (liver, muscle, and testes). This analysis revealed extensive mis-regulation of gene expression and importantly, of Alternative splicing (AS), predominantly affecting exon skipping (SE). These data are of interest, and expands vastly previous observations by the authors, published in their Cell rep paper in 2020.

This analysis was complemented by the use of mouse N2a neuroblastoma cells, that were depleted of Fmr1 using siRNA. As seen in mouse tissues, the authors again observed a role for FMRP in the regulation of AS, predominantly affecting exon inclusion-skipping mode of AS. To validate their results, the authors decreased FMRP expression using genome editing of the Fmr1 pre-mRNA, resulting an in-frame stop codon leading to NMD. Importantly, this strategy resulted in complete loss of FMRP protein. Interestingly, they observed almost identical changes in AS, as those observed following. siRNA-mediated depletion for the Mapt4 mRNA. Next, they developed a Mapt4-based splicing reporter.

Next, Jung and co-authors focused on splicing factors (SFs) that regulate AS in response to FMRP levels. For this, they use the SFMap database to identify putative SFs binding sites. This predicted binding sites for MBNL1, PTBP1, hnRNP F and hnRNP Q in the vicinity of Mapt exon 4, App exon 8 and Tnik exon 21, all of which are regulated by FMRP. They show that FMRP binds to Mbnl1 and also Ptbp1 mRNA. In a series of interesting experiments, the authors determine that FRMP has a dual role in regulating the expression of MBNL1, by affecting splicing of its pre-mRNA, as well as its mRNA translation. In particular, FMR1 promotes exon 5 inclusion in Mbnl1, which encodes an NLS. Thus, in the absence of FMR1, E5 skipping will lead to a predominantly cytoplasmic MBNL1, affecting regulation of splicing.

In summary, there is a lot of work here, the experiments have been carefully designed and most of the conclusions are well supported by the data.  In summary, this is an interesting paper, which highlights that FMRP-controlled mRNA translation and splicing leads to extensive dysregulation of gene expression in the absence of FMRP and could explain phenotypes observed in FXS.

We appreciate the reviewer’s kind comments.

Specific comments

1- The following statement in the Abstract is extremely confusing, mostly taken into account that there are no experiments on Setd2

We have altered the abstract for clarity as suggested.

"In the brain, FMRP stalls ribosomes on specific mRNAs including Setd2, whose encoded protein catalyzes the epigenetic mark H3K36me3. When FMRP is lost, SETD2 levels are excessive, which alters the H3K36me3 landscape and secondarily, alternative premRNA splicing"

This should be moved to the Introduction and the authors should explain better the discrepancy with their own previous results showing an indirect effect of FMRP in splicing via SETD2/H3K36me3

We removed this in the abstract. We have previously mentioned this in the introduction, but have now expanded on it in discussion (page 24).

2- While interesting, results presented in Fig 3 show that FMRP works together with a limited subset of SFs, but this subset was defined by a predictive tool. It still remains possible, that FMRP may work together with other SFs that have not been tested here. This should be discussed.

We have a statement in the discussion stating that FMRP may regulate many splicing factors, not just those presented in this work. Indeed, we now present new Supplemental Figure S9 showing splicing factors whose RNA levels or splicing is regulated by FMRP.

3- Have the experiments following genome editing in Figs 4E, H been extended to the analysis of other mRNAs, besides Mapt4?

We only examined Mapt RNA splicing with the splicing reporter (this is Figure 2H). We have performed a mutation analysis of an MBNL1 binding site in this figure.

We thank all of the reviewers for their very helpful comments, which have improved the manuscript.
