# Supplementary material for: FMRP deficiency leads to multifactorial dysregulation of splicing and mislocalization of MBNL1 to the cytoplasm
Source: PLoS Biol. 2023 Dec 4;21(12):e3002417. doi: 10.1371/journal.pbio.3002417 (PMC10721184; doi:10.1371/journal.pbio.3002417)
Supplement: Attachment — Submitted filename: ResposneToReviewers.docx [file pbio.3002417.s015.docx]

Richard Hodge, PhD

Senior Editor, PLOS Biology

[rhodge@plos.org](mailto:rhodge@plos.org)

Re: PLOS Biology Submission (PBIOLOGY-D-22-02911R2)

Dear Dr. Hodge:

Thank you for having our revised manuscript reviewed for PLOS Biology. We are grateful that 2 of the reviewers accepted the work as is, and the 2^nd^ reviewer had minor comments, which we have addressed in this second revision.

Regarding your focus on the comments of reviewer #2:

- Repeating the experiment in Fig 5C to ensure equal loading. This has been accomplished; please see new Fig 5C.
- Reporting a single heat map in Fig 9E. We have generated a single heatmap that is now presented in supplemental Fig S9C. We have also retained the original heatmap in Fig 9E and Fig 9F because it is much easier to read.
- Performing RT-PCR validate of RNA-seq data in Fig 9G. We have performed the validation that is now presented in Fig 9G, Fig 9H and Fig S9D.

Regarding the editorial/data-related requests.

(A) We have altered the title that is an amalgamation of the original title and your suggestion. This new title now better represents the data in the paper. The new title is:

**FMRP deficiency leads to multifactorial dysregulation of splicing and mislocalization of MBNL1 to the cytoplasm.**

(B) full name of IACUC committee and approval number is included in the Methods

(C) Regarding data availability. We have (1) ensured all data files are uploaded as ‘Supporting Information’ using the format S1_Data.xlsx, etc; (2) data accession code is provided; abetqaqkdfunnez (3) individual numerical values are provided for Figure 1F, 1G, 2C, 2F-H, 3B-D, 4A-C, 5B-D, 7B-E, 8B-C, 8H, 9B, 9D, 9G-H, S2C, S3A-T, S6B, S8A-B in S3 Data.

Raw data of the figures below is available in GEO.

| **Figure** | **Supplementary file name in GEO** |
| --- | --- |
| Fig 1B and Fig 1D | GSE207145_RNAseq.DE.log2FC_Brain.tsv.gz |
| Fig 1I | GSE207145_HC.SE.MATS.JC.txt.gz  GSE207145_CTX.SE.MATS.JC.txt.gz  GSE207145_CB.SE.MATS.JC.txt.gz  GSE207145_LV.SE.MATS.JC.txt.gz  GSE207145_MU.SE.MATS.JC.txt.gz  GSE207145_TE.SE.MATS.JC.txt.gz |
| Fig 8E and Fig 8F | GSE207145_normcnt_DESeq2_input_suna_revision_2023.txt.gz |
| Fig S1A | GSE207145_HC.SE.MATS.JC.txt.gz |
|  | GSE207145_CTX.SE.MATS.JC.txt.gz |
| Fig S1B | GSE207145_RNAseq.DE.log2FC_LV.tsv.gz |
|  | GSE207145_RNAseq.DE.log2FC_MU.tsv.gz |
|  | GSE207145_RNAseq.DE.log2FC_TE.tsv.gz |
| Fig S1C | GSE207145_RNAseq.DE.log2FC_Brain.tsv.gz |
|  | GSE207145_RNAseq.DE.log2FC_LV.tsv.gz |
|  | GSE207145_RNAseq.DE.log2FC_MU.tsv.gz |
|  | GSE207145_RNAseq.DE.log2FC_TE.tsv.gz |
| Fig S1D | GSE207145_RNAseq.DE.log2FC_LV.tsv.gz |
| Fig S1E | GSE207145_RNAseq.DE.log2FC_MU.tsv.gz |
| Fig S1F | GSE207145_RNAseq.DE.log2FC_TE.tsv.gz |
| Fig S1G | GSE207145_LV.SE.MATS.JC.txt.gz |
| Fig S2A | GSE207145_N2a.SE.MATS.JC.txt.gz |
| Fig S9A | GSE207145_normcnt_DESeq2_input_suna_revision_2023.txt.gz |
| Fig S9B | GSE207145_SE.MATS.JC_1.txt.gz |

(D) We will make the data in GEO available before publication

(E) In Fig legends, we will describe where “underlying data can be found”

(F) The S4A-B labeling has been corrected

(G) Data Statement in submission accurately described where data are found in final format.

(H) System/species mentioned in abstract

(I) The blurb:

RNA splicing is widely dysregulated in FMRP-deficient mouse tissues and cells and involves multiple splicing factors including MBNL1. In FMRP-depleted cells, Mbnl1 RNA is mis-spliced, resulting in improper cytoplasmic localization of MBNL1, which further causes impaired of many RNAs.

We hope that our revised manuscript, in which we have performed all experiments requested by reviewers, is suitable for publication in *PLOS Biology*.

Sincerely,

Joel D Richter

Reviewer #1: The authors have satisfactorily responded to my requests.

Reviewer #2 (Jernej Ule, signs review): The authors have performed all key requested experiments, especially RNAseq in combination with rescue using the nuclear or cytoplasmic variants of MBNL1. Fig. 9D shows high correlation between knockdowns of Fmr1 and Mbnl1, which is in support of the model. I congratulate the authors on this complex and interesting work, and my remaining requests are relatively minor and hopefully achievable:

1. In Figure 5C, the western blot of nuclear MBNL1 is normalised to nuclear Lamin B. However, the amount of LaminB1 signal is dramatically higher in the siFmr1 condition, and it seems that the signal might be saturated. It would be interesting to know whether the higher LaminB1 signal is due to inappropriate quantification of protein (and thus unequal loading), or does it reflect a change in LaminB1 abundance in siFmr1 condition? Either way, this experiment should be redone by ensuring that the loading marker shows similar signal in both conditions - if needed, another nuclear marker (histone?) could be used that doesn't change. For now, the nuclear MBNL1 signal seems increased by eye pre-normalisaton, and it remains possible that there is a normalisation artefacts caused by LaminB1 signal saturation in siFmr1 condition. Since the drop in nuclear Mbnl1 abundance is a key aspect of the proposed model, I believe the authors should make some more effort to present convincing evidence.

The experiment in Fig 5C has been repeated to ensure equal loading, as shown in the new Fig 5C.

2. Fig 9E shows that cotransfection of nMbnl1 can rescue many splicing defects of siFmr1, and siMbnl1 can recapitulate many of the changes. However, only those events are shown where rescue/recapitulation is observed, so the heatmap can't inform on the extent of the relationship. Instead of showing two separate heatmaps in Fig 9E/F, a single heatmap should be shown that includes all SEs that pass a certain threshold for significant change upon siFmr1, and all conditions are shown in separate columns (including siMbnl1).

A single heatmap is reported in supplemental Fig S9C. We have also retained the original heatmap in Fig 9E and Fig9F because it is much easier to read.

3. Fig 9g shows PSI values of representative spliced exons, and as I understand this is showing RNAseq data. Since these RNAseq results provide key evidence for the model, it would be helpful to validate a few exons with a different method. In particular, few exons from current Fig 9E could be randomly selected (i.e., those where Mbnl1 expression seems to rescue the change upon siFmr1) and validated with RT-PCR & electrophoresis (including siFmr1, siMbnl1, and rescue of both with nuclear Mbnl1 if possible).

We have performed the RT-PCR validation of RNA-seq data that is now presented in Fig 9G, Fig 9H, and S9D.

Reviewer #3: The authors performed a thorough revision and have addressed the minor concerns and suggestions that I raised during the first round of review. Thus, I remain enthusiastic about this study. I do recommend publication in PLoS Biol.
